# Vialess heterogeneous skin patch for multimodal monitoring and stimulation

**DOI:** 10.1038/s41467-025-55951-6

**Published:** 2025-01-14

**Authors:** Hyeokjun Lee, Soojeong Song, Junwoo Yea, Jeongdae Ha, Saehyuck Oh, Janghwan Jekal, Myung Seok Hong, Chihyeong Won, Han Hee Jung, Hohyun Keum, Sangyoon Han, Jeong Ho Cho, Taeyoon Lee, Kyung-In Jang

**Affiliations:** 1 Department of Robotics and Mechatronics Engineering, Daegu Gyeongbuk Institute of Science and Technology (DGIST), Daegu, Republic of Korea; 2 ASML Korea, Hwaseong, Republic of Korea; 3 School of Electrical and Electronic Engineering, Yonsei University, Seoul, Republic of Korea; 4 Department of Information and Communication Engineering, Hannam University, Daejeon, Republic of Korea; 5 Industrial Transformation Technology Department, Korea Institute of Industrial Technology (KITECH), Cheonan, Republic of Korea; 6 Department of Chemical and Biomolecular Engineering, Yonsei University, Seoul, Republic of Korea

**Keywords:** Health services, Biomedical engineering

## Abstract

System-level wearable electronics require to be flexible to ensure conformal contact with the skin, but they also need to integrate rigid and bulky functional components to achieve system-level functionality. As one of integration methods, folding integration offers simplified processing and enhanced functionality through rigid-soft region separation, but so far, it has mainly been applied to modality of electrical sensing and stimulation. This paper introduces a vialess heterogeneous skin patch with multi modalities that separates the soft region and strain-robust region through folded structure. Our system includes electrical and optical modalities for hemodynamic and cardiovascular monitoring, and a force-electrically driven micropump for drug delivery. Each modality is demonstrated through on-demand drug delivery, flexible waveguide-based PPG monitoring, and ECG and body movement monitoring. Wireless data transmission and real-time measurement validate the feedback operation for multi-modalities. This engineered closed-loop platform offers the possibility for broad applications, including cardiovascular monitoring and chronic disease management.

## Abstract (web summary)

For system-level integration of wearable electronics, folding method offers simplified processing and enhanced functionality through rigid-soft region separation. Here, the authors introduce a heterogeneous skin patch with multimodal biosensing and responsive drug delivery through folded structure.

## Introduction

With the advent of an aging society, the field of wearable biomedical devices has rapidly advanced^1^, highlighting personalized healthcare. For user-centered healthcare through wearable devices, many studies have been conducted in various approaches and wearable configurations such as wristbands, electronic skin, textiles, microneedle, and contact lens etc^2–8^. These studies mainly focus on effective biosensing to discover physiological factors or timely treatment through drug delivery^9^. In terms of biosensing, many physiological features such as mechanical deformations^10^, chemical analytes^11,12^, optical parameters^13,14^, and electrophysiological factors^15^ have been detected through each sensing mechanism. Moreover, multiplexed analysis expedites health monitoring and management by detecting several clinical conditions simultaneously, providing comprehensive information beyond what a single analysis can offer^16,17^. In addition to biosensing, the incorporation of a biosensing system and drug delivery system as a closed-loop system is crucial for satisfying personal healthcare requirements and overcoming the limitations of passive therapy. Extensive efforts have been made to develop the closed-loop electronic system which integrates both biosensing and stimulation through wearable or implantable approaches^18–22^. However, many challenges still remain for achieving the simultaneous functionality of biosensing and controlled drug delivery within a single device^9^. For example, automated microneedle patches have been developed to be responsive to external stimuli such as heat, mechanical strain, glucose concentration, and hypoxia^23–26^. However, implementing the sensing and drug delivery function in the microneedle itself has limitations in providing complex biometric information and ensuring continuous use. These limitations can be overcome by integrating independent multiplexed biosensing modules with a standalone controllable drug delivery module, but such a system has not yet been developed. In addition, for wearable applications, the biosensing and drug delivery devices must be combined with flexibility and stretchability, ensuring conformal contact with the skin. Although many ultrathin and flexible devices have been developed to enable conformal contact^27,28^, expanding those to a system level entails incorporating various components such as power management, communication modules, signal processing modules, and drug loading chambers. Attempting to incorporate these bulky and rigid components alongside sensors and drug delivery units into a single-layer structure on a soft substrate can lead to significant challenges. For instance, due to skin deformation, such a configuration can create stress concentration at the interfaces between rigid and flexible components, increasing the risk of device delamination. This instability negatively impacts the quality of sensed data and the effectiveness of drug delivery by disrupting the device’s intimate contact with the skin. To address these issues, a multi-layer approach is essential, as it allows for more effective integration and stress management across different layers. Multilayered structures can distribute mechanical stress more evenly, improve mechanical adaptation to dynamic skin movements, and enhance spatial efficiency rather than spreading them in a single plane. As one of the integration methods, stacking through via-hole processes has been predominantly used^29–32^. Reliable methods for via-hole fabrication and filling have been well-established for conventional electronics; however, these approaches present limitations when applied to flexible and stretchable printed electronics^33^. Via-hole structures vary in design based on their application (e.g., electrical connections, optical pathways, or fluidic channels)^34–36^, requiring precise alignment and integration with different materials, such as conductive pastes or SU-8, which increases process complexity. Furthermore, flexible materials commonly used in wearable devices, such as polyimide, Ecoflex, and PDMS, exhibit different coefficients of thermal expansion (CTE), increasing the risk of alignment mismatches^37^. During the drilling process, these materials are also prone to damage, raising concerns about fluid leakage and device integrity.

As an alternative to the via-hole process, a method has been developed that fabricates overall systems in a single layer and then folds them at once^38–40^. Despite these advantages of the folding approach in terms of integrity and functionality, the folded structure of wearable devices has so far been applied only to implementing electrical connections in a folded structure. This approach simplifies the fabrication process by removing via holes, facilitating the integration of multiple modalities. In addition, it allows for conformal contact with the skin, even with the inclusion of rigid components, thereby enabling effective multimodal biosensing and drug delivery.

Here, we introduce a wireless, folded multi-modal device (fMMD) with a mechanically isolated heterogeneous structure capable of simultaneous biosensing and drug delivery. The proposed multi-modal system includes the monitoring of quantitative information on hemodynamic and cardiovascular health states based on electrical and optical modalities and a force-electrically driven micropump-based drug delivery system as a chemical modality. We demonstrate the functionality of each modality, such as the on-demand drug delivery, photoplethysmogram (PPG) monitoring using flexible waveguides, and electrophysiological monitoring of electrocardiograms (ECG) and body movement. As a strategy for system integration, we design functionally sectioned islands on flexible printed circuit boards (FPCB), connecting each island with serpentine interconnects. This structure is robust against deformation and stretchable, making it widely used in both industrial and research fields of wearable technology^40–42^. By folding the serpentine FPCB and adding a strain-isolating layer between the upper and lower layers, we achieve strain robustness when in contact with the skin. Through wireless data transmission and real-time measurement, we demonstrate the feedback operation of the fMMD for drug delivery triggers via real-time monitoring. We establish an engineering closed-loop platform for intelligent healthcare, which can be applied not only to the cardiovascular monitoring demonstrated in this paper but also to various applications such as monitoring of blood glucose, pain, and chronic diseases^43–47^.

## Results

### Design and system architecture of fMMD

**Fig. 1:**
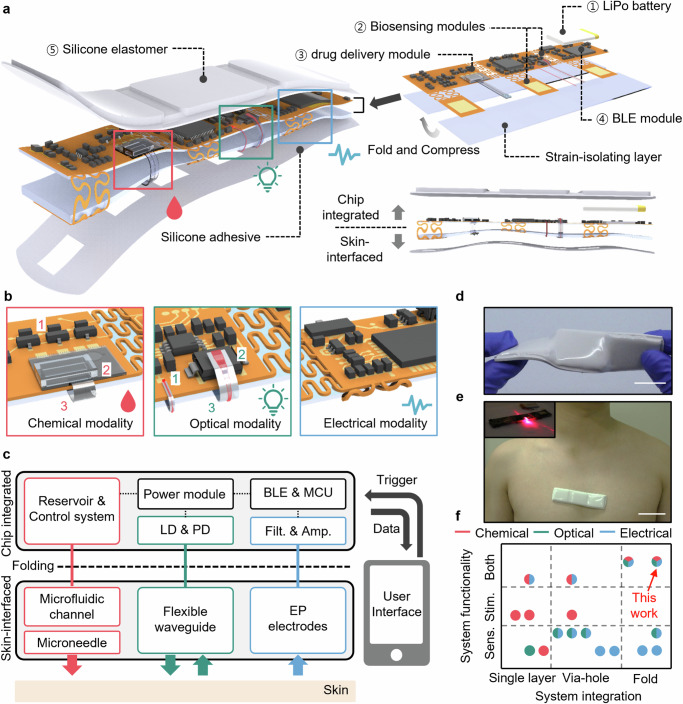
Schematic illustrations of vialess heterogeneous patch with folded structure for simultaneous monitoring and stimulation. **a** Integrating and folding process of fMMD with multilayer components of chemical, optical, and electrical modalities. Each modality is denoted by red, green, and blue colored boxes, respectively. Each component is designed to be divided into rigid and soft layers. **b** Schematic images of chemical, optical, and electrical modalities labeled in red, green, and blue colored boxes. **c** Overall system diagram of fMMD showing the functional components divided with the chip-integrated (rigid) and skin-interfaced (soft) area for intimate skin contact. **d** A Photo of encapsulated fMMD under twisting. Scale bar, 2 cm. **e** A photo of encapsulated fMMD attached on the chest. Scale bar, 5 cm. The inset indicates a photo showing light guiding with the optical waveguide in the folded state. **f** Graphically comparison of proposed fMMD and previously reported devices according to the system integration and functionality.

Figure 1a schematically depicts the integration process of the mechanically flexible and wireless fMMD along with assembling and folding functional components. The fMMD reported here is mainly composed of five constituent parts: (1) a lithium-polymer rechargeable battery with linear charge management controller of 85 mA charge current, (2) biosensing modules to record cardiac activity, movements, and skin temperature using electrical and optical modalities, (3) force-electrically responsive drug delivery module as chemical modality, (4) Bluetooth Low Energy (BLE) communication module to collect biosensing signals and trigger drug delivery wirelessly, and (5) silicone elastomer for soft encapsulation. For effective placement and adaptation on the curved skin, the overall layout is designed as an FPCB with an island-bridge structure, where the bridge enables bending and stretching through the serpentine interconnect. Moreover, the integrated fMMD goes through the folding and compressing steps, which serve not only to reduce surface area but also prevent bulky components, such as drug reservoir, battery, and mounted integrated circuit (IC) units, from interfering with conformal contact. A silicone elastomer (500 µm thick) with low Young’s modulus (50 kPa) provides a strain-isolating effect between the skin-contact side and non-contact side to mitigate the stresses at the non-contact side islands for uniaxial stretching of up to 20 %^40^. Figure 1b represents the three modalities for closed-loop systems based on simultaneous biosensing and drug delivery. In terms of biosensing, ECG, body movement, and skin temperature are acquired through folded serpentine interconnects (Fig. 1b, right). In addition to electrical signals, PDMS-based flexible optical waveguides are used to measure PPG signals. The rigid and thick components, such as (1) laser diode (LD, 650 nm wavelength) and (2) photodetector (PD), are mounted on the chip-integrated side, and (3) the two flexible waveguides are well aligned with the light source spot of LD and the light receiving spot of PD using optical epoxy, respectively (Fig. 1b, center). The folded waveguides aligned with the LD and PD serve as guiding the light to the skin and the reflected light from the skin. In terms of controlled transdermal drug delivery, the drug delivery system is composed of three functional components (Fig. 1b, left): (1) injection trigger and pumping system with the performance of steady flow rate, (2) drug storage and microfluidic channel for delivery from reservoir to skin, and (3) microneedle array for penetration into the skin. The thermo-pneumatic micropump actuation based on a microheater array provides a continuous pressure gradient by controlling a general-purpose input/output (GPIO) sequence, which can induce force-electrically driven operation. The rigid, thick components are placed on the chip-integrated side, and the soft, thin components are placed on the skin-interfaced side as shown in Fig. 1c. Each modality is integrated with a single platform (Supplementary Fig. 1a), and the folded configuration serves to define sections separating the elements that are in contact with the skin from those that are not (Supplementary Fig. 1b, c). Each functionality can be powered by a power module, which consists of a rechargeable LiPo battery, power switching circuitry, a voltage regulator, and a battery charger IC. The battery can be recharged through a USB port, ensuring continuous operation of the device. Bluetooth low energy (5.0) system on a chip with a Bluetooth radio enables wireless data transmission and real-time feedback for the collected and processed data within the operation range of Class 2. The integrated and folded electronic system is sealed with top silicone enclosure and bottom silicone adhesive layers for improving the soft mechanical properties for deformations such as twisting and bending (Fig. 1d and Supplementary Fig. 1d) and the attachment on wearer’s skin (Fig. 1e). Based on this system architecture and configuration, the proposed fMMD has better functionality than other reported flexible, stretchable standalone devices, which enable the triggering of drug delivery operation as feedback of multimodal monitoring (Fig. 1f and Supplementary Table 1).

### Mechanical properties of the fMMD

**Fig. 2:**
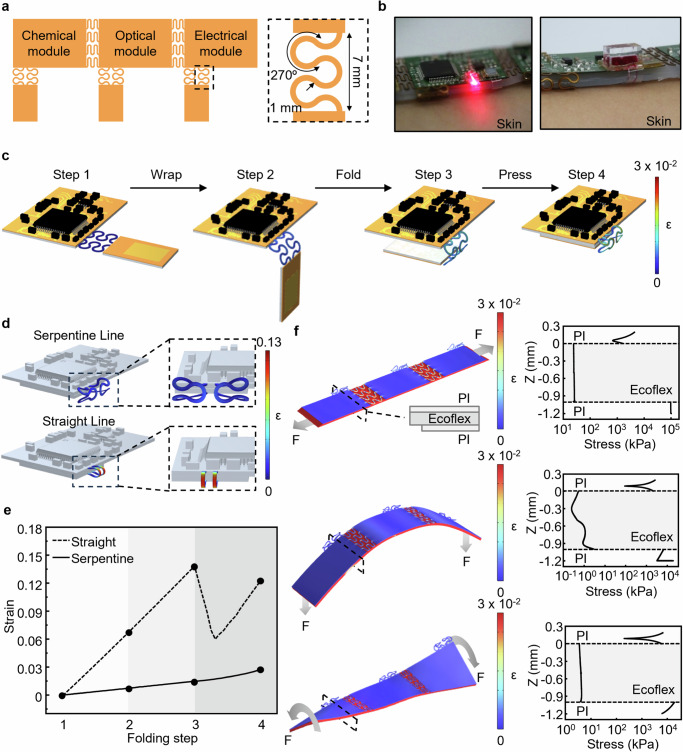
Design and Mechanical Properties of the fMMD. **a** Schematic design of the island-bridge substrate. Chemical, optical, and electrical modules are attached to the substrate, which ensures structural stability through serpentine interconnects (black dashed box). **b** Photos of the fMMD attached to the skin representing different modalities. **c** Folding process of the substrate with a module and FEA simulation results in strain on the serpentine interconnects for each step. **d** Mechanical analysis of the principal strain in a folded state for interconnects designed with serpentine lines versus straight lines. **e** Comparison of maximum strain on serpentine and straight interconnects during the folding process. **f** Strain and stress of fMMD under multi-deformation modes (stretching, bending, twisting). The strain-isolating effect was observed for the deformations applied to the skin-interfaced layer through stress analysis at a cross-sectional plane (black dashed box).

The overall layout implementing the island-bridge structure with serpentine interconnects is illustrated in Fig. 2a. The chip-integrated substrate is designed to attach sub-systems, including biosensing modules, drug delivery modules, and BLE communication modules. The optical image of the entire fMMD with these sub-systems in a folded state is shown in Fig. 2b. As previously mentioned, the layout with the sub-systems attached integrates strain-isolating layers in both the chip-integrated layer (non-skin-contact side) and the skin-interfaced layer (skin-contact side).

Serpentine interconnects effectively distribute the stress encountered during the folding process, thereby reducing the maximum strain on the interconnects and ensuring mechanical stability and connection reliability. In Fig. 2c, the strain on the serpentine interconnects during the folding process of the fMMD is analyzed through simulation. In Step 1, the initial state, the folding process begins by wrapping one side of the substrate. In this phase, the substrate starts to fold along the radius of rotation. The folding process continues until Step 3, where the ends of the chip-integrated and skin-interfaced layer align. Finally, in Step 4, the folding process is completed by pressing the substrate, ensuring that the chip-integrated and skin-interfaced layers come into contact. At each step, the strain on the serpentine interconnects remains below 0.03, indicating stable connections throughout the folding process. The strain-isolating polymer layer integrates the entire layout horizontally, ensuring that the interconnects connecting the chip-integrated and skin-interfaced layers follow the same mechanical dynamics. Therefore, the entire fMMD maintains stability during the folding process. To confirm the enhanced mechanical stability provided by the serpentine interconnect design, a finite element analysis (FEA) comparison between interconnects designed with serpentine lines and those with straight lines was conducted as shown in Fig. 2d, and the maximum strain on the interconnects during the folding process for each design is comparatively analyzed in Fig. 2e. The straight-line design exhibits a rapid strain increase to approximately 0.14 during the wrap and fold process, followed by a slight decrease and then an increase to about 0.12 during the press process. In contrast, the serpentine line design effectively disperses the stress, maintaining the maximum strain below 0.03 throughout the entire process. During the wrap and fold section, strain increases linearly, however, in the press section, the islands are pressed while the interconnects remain unfixed and deform differently. In particular, for the straight interconnect design, a temporary decrease in strain is observed in the press section. This phenomenon appears to occur due to a temporary increase in the curvature radius of the interconnect in this section. The curvature radius of the straight interconnect throughout the entire folding process is illustrated in the graph shown in Supplementary Fig. 2a.

In addition, the entire fMMD needs to be easily deformable to guarantee conformal contact with the skin, and the strain isolation between the skin-contact side and the non-skin-contact side can prevent performance degradation of the sub-systems attached to the substrate. To verify the overall deformability provided by the serpentine interconnect design and the strain isolation effect of the silicone elastomer, FEA was performed. The results when deformation is applied to the skin-interfaced layer are shown in Fig. 2f. The configuration of the fMMD, including the strain when multi-deformation modes (stretching, bending, and twisting) are applied to the skin-interfaced layer, is shown in Fig. 2f. The strain associated with multi-deformation modes is illustrated, and the stress can be observed in the graph. Similarly, strain and stress analysis for the encapsulated device was conducted under various deformation modes (stretching, bending, and twisting), confirming that the strain in the PI upper layer, where the actual chip is integrated, remains within 0.03 in both the presence and absence of the encapsulation layer (Fig. 2f and Supplementary Fig. 3). This demonstrates that the polymer layer between the two substrates effectively prevents the deformation of the skin-interfaced layer from affecting the chip-integrated layer during these deformations. This strain-isolating layer should be made from a polymer with a low Young’s modulus. When the strain-isolating layer is made from polyimide, the same as the substrate, the average strain in the chip-integrated layer, where various modules are integrated, increases to 0.04 as the strain in the fMMD’s skin-interfaced layer increases up to 15%, according to simulations. In contrast, when the strain-isolating layer is made from a polymer, Ecoflex, the strain remains below 0.001 (Supplementary Fig. 2b, c). These results confirm that the fMMD’s serpentine interconnects design and silicone elastomer layer effectively enhance deformability and provide strain isolation, ensuring stable performance during use.

### Design and operation of on-demand drug delivering modality

**Fig. 3:**
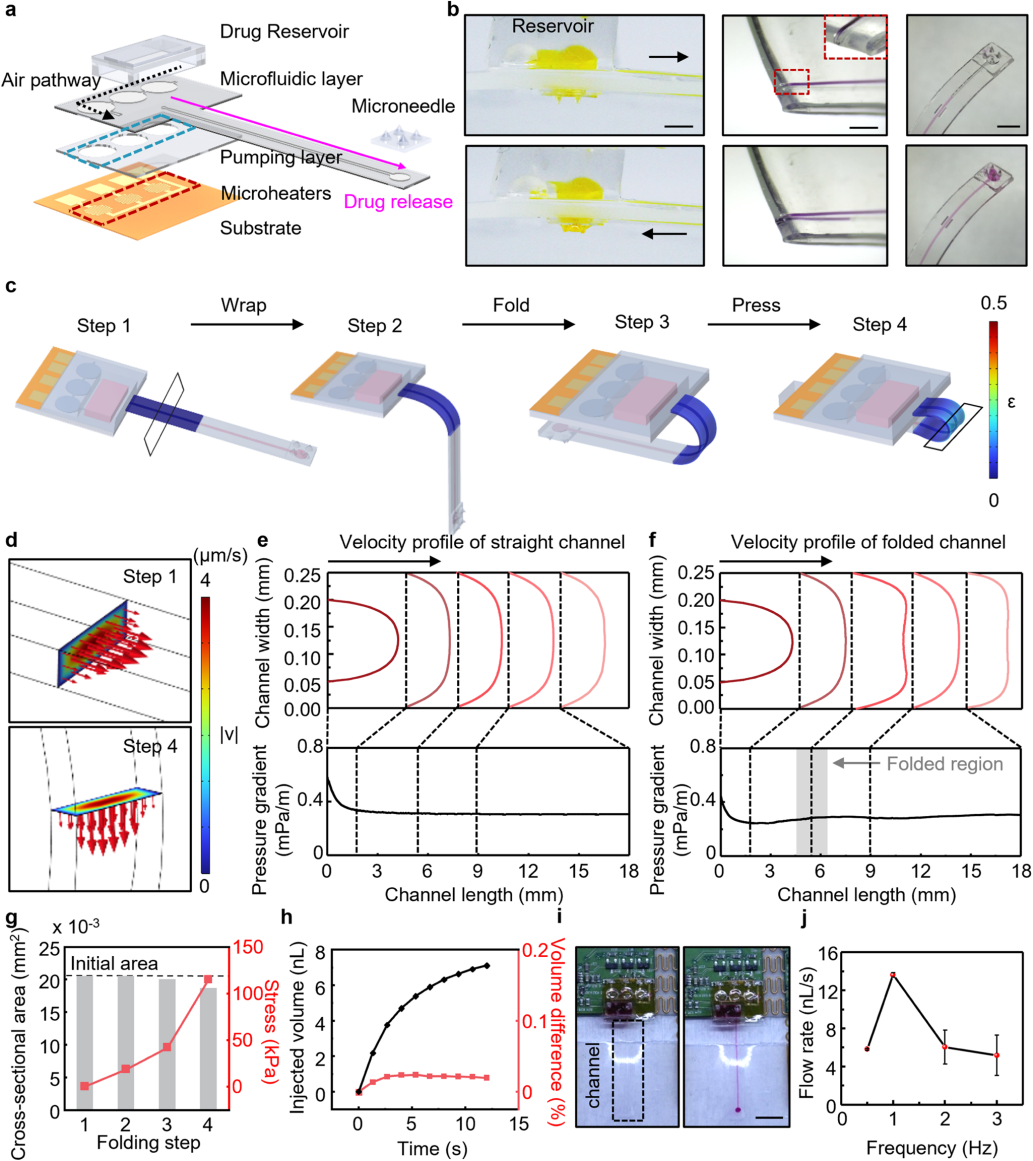
Design and operation of on-demand drug delivery device with peristaltic pumping. **a** Exploded view of the micropump-based microfluidic system, composed of a soft drug delivery channel with a drug reservoir, a pumping membrane layer, and a microheater array for thermo-pneumatically pumping actuation. **b** Optical images of drug delivery modality with folded state. The drug reservoir is located at the upper area of the fMMD, and the microneedle array is located at the end of the microfluidic channel to penetrate the skin and deliver the liquid drug percutaneously. Scale bar, 1, 5, and 2 mm, respectively. **c** FEA simulation showing the stress distribution on the microfluidic channel of DDD during the folding process. **d** Velocity magnitude analysis in the channel at the same location for unfolded versus folded states. Velocity profile and pressure gradient along the microfluidic channel (**e**) before and (**f**) after folding. **g** The change of cross-sectional area and stress simulation result for the microfluidic channel during the folding process. **h** Total delivered fluid volume at the folded state and the delivered volume difference between the folded and unfolded states. **i** Demonstration of peristaltic pumping operation (Supplementary Movie 1). Scale bar, 1 cm. **j** The flow rate of DDD depends on the switching frequency of the pumping mechanism. Error bars represent the standard deviation for flow rate according to each pumping frequency (*n* = 3, independent measurements).

Figure 3a, b presents the schematic diagram and optical images of the folded drug delivery device (DDD) with peristaltic micropump, which highlights the pump architecture to take the external air inside and push loaded drug out to the transdermal skin through the 2 × 2 microneedle array. This drug delivery system is composed of five functional components: (1) drug reservoir, which stores and refills the drug up to tens of μL, (2) microfluidic layer with channel (100 × 100 μm^2^) for drug delivery, (3) pumping layer for peristaltic operation via sequentially thermal expansion of three actuation chambers, (4) flexible substrate patterned with planar electro-resistive microheaters (Cr/Au; 7 nm/200 nm in thickness), and (5) microneedle array with hole, enabling spontaneous flow of delivered liquid driven by capillary force and surface tension^48,49^. Further details of the fabrication of the microneedle can be found in the method section, and Supplementary Fig. 4. The peristaltic operation in the air pathway above the micropump induces the delivered air into the reservoir to push out the drug^50,51^. After the integration process, functional parts for peristaltic pumping such as microheaters, peristaltic micropump, and reservoir are located on the chip-integrated side, and the microfluidic channel (2.5 mm width, 20 mm length), and microneedle part (~ 700 μm height) can be in intimate contact with the skin through the folded state (Fig. 3b and Supplementary Fig. 5). The PEGDA microneedle array located on the skin-interfaced side is designed to have a fracture force of 0.6 N per needle for skin penetration (Supplementary Fig. 6a–c). The pumping layer consists of three actuation chambers with deformable diaphragm membranes, which are deterministically aligned with microheaters to activate each diaphragm membrane independently. When an actuation chamber is inflated by Joule heating, the elevation of the diaphragm membrane (~ 30 μm) induces positive air pressure inside the microfluidic layer. Conversely, recovery of the diaphragm membrane due to cooling induces negative air pressure inside the microfluidic layer, which retakes the external air in to balance out the pressure difference. The sequence of actions of pumping and recovery of the three diaphragm membranes causes peristaltic actuation force-electrically. The cyclic operation of the GPIO sequence enables continuous drug delivery until the drug is fully consumed from the reservoir. Supplementary Fig. 7 presents the temperature of the microheater array during micropump operation. The temperature difference caused by turning the heater on/off causes pressure changes, which converge to about 3 degrees over time.

During the folding process, although the microfluidic channel is subjected to stress by deformation at the folding site, the buckling of the microfluidic channel accommodates the excessive load associated with compressing (Fig. 3c). Despite this deformation, the change of velocity magnitude remains negligible between the unfolded and folded states as shown in Fig. 3d. Velocity profiles are examined at the microfluidic channel’s inlet point, three bending points, and the outlet point, and an analysis of the pressure gradient throughout the channel confirmed that the folded configuration exerts impact on the pressure gradient below 9% at the maximum bending point (Fig. 3e, f). Moreover, as shown in Fig. 3g, while the stress on the channel increases, the change in the microfluidic channel area remains below 3%. Based on analysis of design parameters^52,53^, FEA results shown in Fig. 3h show the injection volume of the drug during the peristaltic pumping process with design parameters (R, 0.8 mm; W, 100 μm; θ, 10°). Per each pumping cycle, induced positive air pressure starts to release the drug. As the cycle progresses, temperature oscillation stabilizes due to the residual temperature, which means that the injection volume per cycle becomes saturated. Also, the net flow difference before and after folding is less than 0.1% due to the negligible area change of the microfluidic channel. For a demonstration of DDD after integration with the fMMD, a peristaltic micropump-based drug delivery module as a chemical modality is mounted on the designed FPCB and is electrically connected with FPCB using a conductive paste. Figure 3i and Supplementary Movie 1 show the drug delivery operation which is triggered wirelessly after module integration. The sequential expansion and contraction of the pumping membrane, which is aligned over the microheater array, causes the dyed water in the drug storage chamber to be transferred to the outlet of the microfluidic channel under pressure. In addition, control parameters, such as the switching frequency, can adjust the flow rate due to the control of the amount and velocity of volume expansion at each actuation chamber. About the drug delivery system for fMMD with a fixed applied voltage of 3.3 V and diaphragm radius of 1.2 mm, the flow rate can be controlled by changing the switching frequency. The switching frequency of 1 Hz provides the maximum flow rate above 12 nL/s (Fig. 3j), while the higher switching frequency shows a reduced flow rate due to decreasing applied pressure caused by insufficient cooling of the microheater. Further details of the flow rate measurement can be found in the experimental section and Supplementary Fig. 8.

### PPG monitoring based on flexible PDMS waveguides

**Fig. 4:**
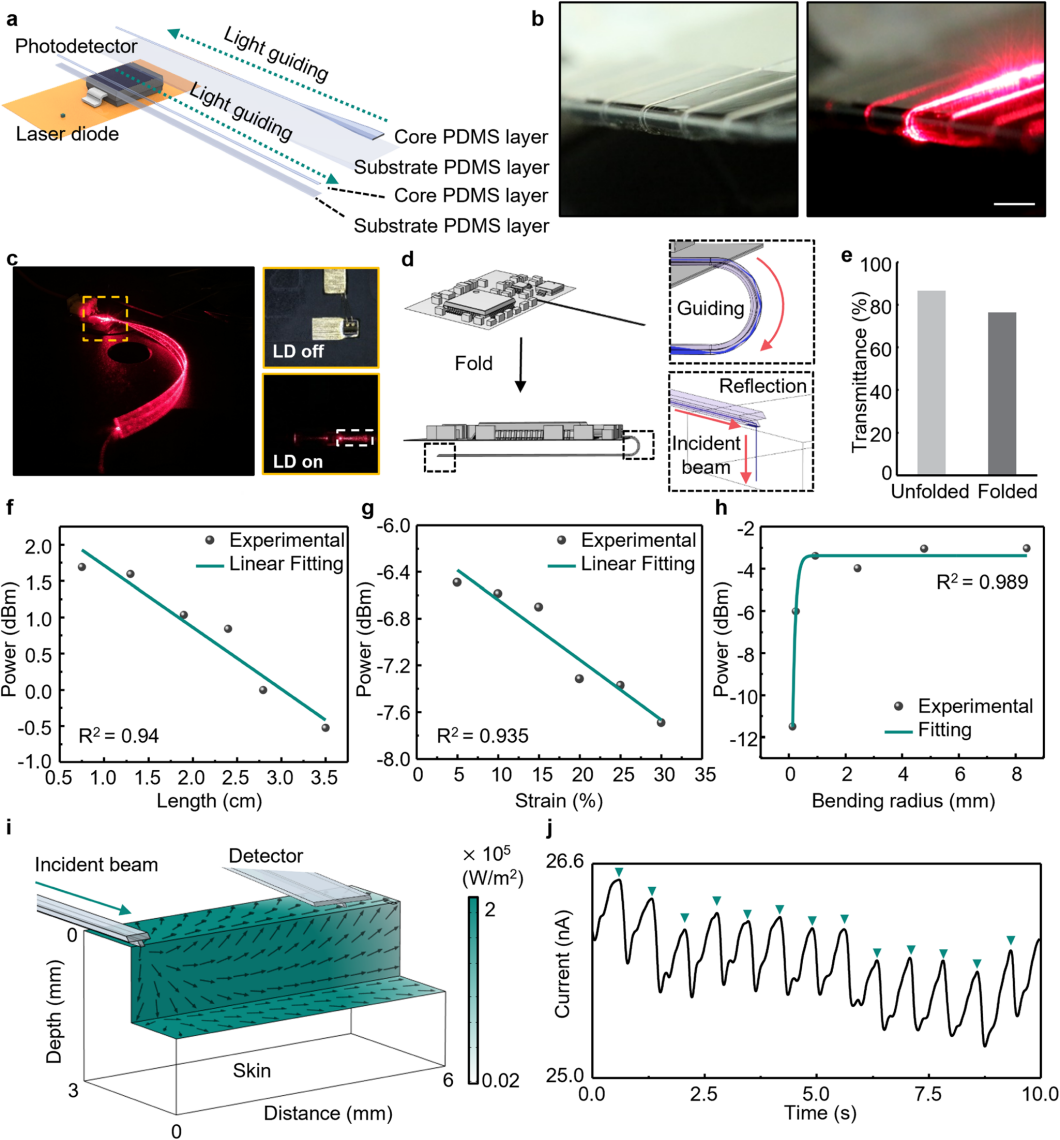
Optical characterization and biosensing based on flexible monolithic PDMS waveguides. **a** Exploded view of the optical biosensing modality with a pair of flexible PDMS waveguides, aligned with a laser diode and a photodetector. **b** Optical image of the light guiding effect of a PDMS waveguide in the folded state aligned with the optical fiber (bending radius, 250 µm). Scale bar, 500 µm. **c** Optical image of the light transmitting system composed of LD and monolithic PDMS waveguide (wavelength, 650 nm). **d** Ray Optics simulation model showing the change in optical guiding performance of a flexible waveguide by folding. The light is guided within the folded waveguide, composed of two PDMS materials with different refractive indices, as indicated by the upper dashed box. The end of the waveguide is cut for out-of-plane reflection, which allows the light to be guided perpendicular to the skin as shown as a lower dashed box. **e** The comparison for transmittance of flexible PDMS waveguide with and without folding. Optical characterization according to the (**f**) propagation loss, (**g**) stretching loss, and (**h**) bending loss along the PDMS waveguide at 650 nm. **i** FEA simulation showing the diffuse distribution of light irradiated from 45° edge-cut surface of a waveguide in skin tissue (**j**) Real-time PPG monitoring using light transmitting and receiving system based on PDMS waveguides.

**Fig. 5:**
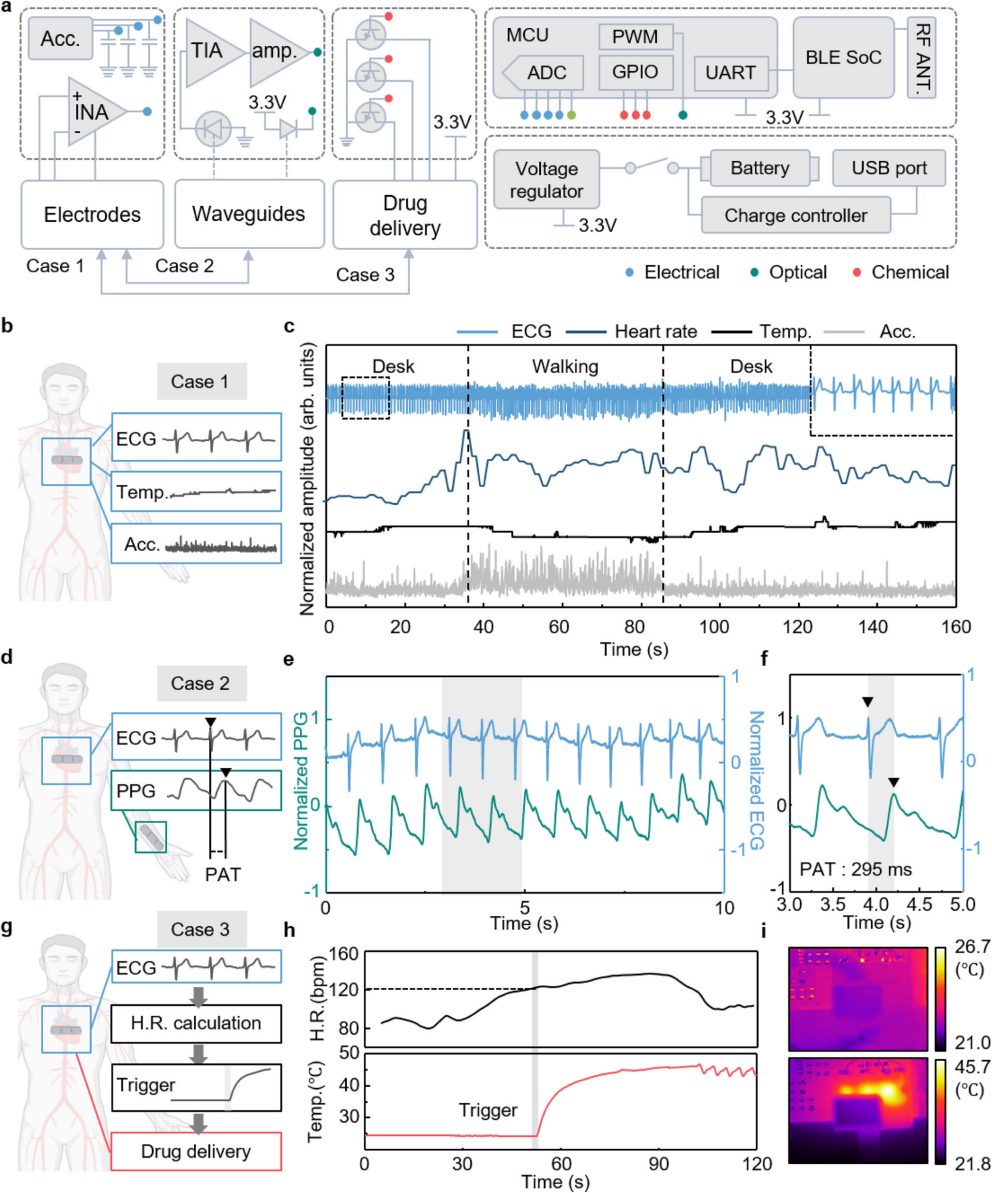
Demonstration of the fMMD for real-time multiplexed monitoring and closed-loop operation. **a** Circuit diagram showing the overall functionalities of fMMD. **b** Schematic showing fMMD attachment locations for multi-electrophysiological signals monitoring (case 1. multi-modal biosensing at single spot). Created in BioRender. Lee, H. (2025) https://BioRender.com/j29r450. **c** Demonstration of the fMMD application as an electrophysiological monitoring tool of ECG, HR, skin temperature and body movement during desk working and walking. **d** Schematic showing fMMD attachment locations for pulse arrival time monitoring. Each dotted box represents an MMD attached to the wearer’s chest and arm (case 2. single modal biosensing at multi-spots). Created in BioRender. Lee, H. (2025) https://BioRender.com/j29r450. **e** Measurement results of ECG and PPG for electrical-optical biosensing. **f** Calculation of PAT from the time interval between peaks of each waveform highlighted in the black dashed box. **g** Schematic showing the principle of feedback operation of fMMD through ECG monitoring and drug delivery triggering (case 3. multi-modal feedback operation at single spot). Created in BioRender. Lee, H. (2025) https://BioRender.com/j29r450. **h** Real-time measurement of HR and temperature of microheater indicating initiation of drug delivery operation. Drug delivery is automatically triggered when the heart rate exceeds 120 bpm (Supplementary Movie 2). **i** IR thermal images of the fMMD before and after sham drug delivery trigger.

Figure 4a presents the exploded schematic view of the optical system based on a pair of flexible PDMS waveguides. For the PPG monitoring, one waveguide is aligned to the beam spot of a laser diode (LD) with a wavelength of 650 nm, which is responsible for transmitting the red light from the LD located on the top surface of the fMMD to the skin. The other waveguide is aligned with the photodiode (PD) to collect and guide light reflected from the skin. To build an entire system, a number of factors are required, such as low optical attenuation for bending or stretching, simplicity of fabrication, and coupling with the commercial diode. PDMS can provide suitable attributes based on the low Young’s modulus and easy fabrication of a variety of patterns (Fig. 4b and Supplementary Fig. 9). In addition, the refractive index can be adjusted by changing fabrication conditions such as the mixing proportions of prepolymer-curing agent and the curing temperature^54,55^_._ These properties make it possible to fabricate monolithic PDMS waveguides in which both the core material and the cladding or substrate material are PDMS, greatly simplifying the fabrication process. The ridge PDMS waveguide with the core-substrate interface is fabricated by the micro-molding process with patterned SU-8 mold (Supplementary Fig. 10). For waveguiding effect in core PDMS, core PDMS (mixing ratio of 2.5:1) and substrate PDMS (mixing ratio of 20:1) have a refractive index of about 1.42 and 1.41 for the wavelength of 650 nm, respectively^55^. Furthermore, the size of the cross-sectional area of the core PDMS can affect the number of modes that the core PDMS can carry, which is critical for the intensity of light delivered to the PD. For PPG monitoring, the size of the cross-sectional area of core PDMS is fixed at 100 μm × 100 μm to align with the LD. For transmitting red light to the skin, a fabricated PDMS waveguide (2.5 cm length) is well-aligned with wire-bonded LD using a 3-axis stage (Fig. 4c), and LD is controlled by pulse width modulation (PWM) with frequency of 200 kHz and duty cycle of 1% to generate the maximum intensity of light (5 mW). The aligned waveguide is folded to transmit the light in contact with the skin. The design of the ridge and core PDMS with differing refractive indices ensures that the light is effectively guided to the terminus of the waveguide, even in a folded state. In addition, the end of waveguides is cut off at a 45-degree angle with a laser blade to ensure the incident beam direction from horizontal to perpendicular to the skin, as shown in Fig. 5d^56^. Although there is some loss of guiding light due to folding, the transmittance remains above 70%, which is sufficient to monitor the PPG signal (Fig. 5e). In addition, supplementary Fig. 11 examined the effects of potential shape deformations on transmittance when the device is attached to the skin during movement. The FEA analyzed transmittance variations resulting from changes in bending radius (± 4 mm from the initial radius) and lateral displacements (0.5, 1.0, and 1.5 mm) between the skin-adhered lower waveguide channel and the laser-coupled upper channel. Under these mechanical deformations, the normalized transmittance decreases by less than 15% compared to the undeformed state when the device is attached to the skin.

The stretchable and bendable optical waveguiding module is characterized in terms of propagation losses, stretching losses, and out-of-plane bending losses. Further details of the measurement setup can be found in Supplementary Fig. 12. To determine the propagation loss per unit length of the waveguide, a cutback process is performed from 3.5 cm length to 0.75 cm length as shown in Fig. 4f. Silicon PD (0.32 mA/W sensitivity; Thorlabs) measures the emission light intensity of the waveguide using a calibration curve and converts to resulting power in decibel-milliwatts (dBm), thereby the proposed optical module shows the propagation losses of − 0.22 dBm/cm. Figure 4g shows the optical losses for stretching the waveguide from 5 % to 30 %, which is the maximum strain that can occur in biosensing. The optical adhesive minimizes the change of coupling conditions at the interface with LD and waveguide during the elongation. We observe the linear response curve of power loss with − 0.056 dBm/% using a waveguide with a 3.5 cm length. Figure 4h presents the optical losses for mechanically induced out-of-plane bending, which occur in the integration process of MDD. As shown in Fig. 4h, in the range of radius of curvature up to 8 mm, bending has a negligible effect on bending radius greater than 1 mm due to the ultrathin and flexible properties of the PDMS waveguide itself. When the bending radius is smaller than 1 mm, the losses increase dramatically due to the change of multimode conditions at the folding region.

Incident beams diffuse in the skin tissue, with photons reaching as far as up to 6 mm from the source (Fig. 4i). Reflected light is guided to the waveguide with a tapered structure. Tapered waveguide facilitates adiabatic mode transformation through their gradual structural changes, minimizing signal attenuation and enhancing transmission efficiency. Their wide input section effectively collects scattered light and directs it into a narrow output, providing high coupling efficiency. These characteristics contribute to the efficient guiding of light from broad sources and reduce losses in tapered waveguides. For the PPG monitoring using the proposed optical module based on PDMS waveguides, two waveguides are aligned with LD and PD, respectively, and the end of the waveguides is cut off at a 45-degree angle with laser blade to ensure that the light is transmitted perpendicular to the skin and is designed with tapered edge to enhance the coupling efficiency. Photocurrent measured from the PD shows the pulse signal with a non-pulsatile level of 25 ~ 26 nA and a pulsatile level of about 1 nA as shown in Fig. 4j. It demonstrates that the proposed optical modality can monitor the parameter related to blood flow, which can be a potential tool for multiplexed analysis with electrophysiological monitoring.

### Real-time multiplexed monitoring and closed-loop operation

To validate the system-level performance of the fMMD as a biomedical application, FPCB is designed to allow the integration of power management, wireless data communication, signal processing, and individual modalities including electrical, optical, and chemical modules as shown in Fig. 5a. The integrated fMMDs are respectively mounted on the chest and wrist for the real-time measurement of electrophysiological and multiplexed cardiovascular signals. Light guided through a flexible waveguide and fluid delivered through a microfluidic channel are delivered to the skin along a folded curve. As a demonstration of electrical modality during daily activity, the fMMD is attached to the wearer’s chest while the multi-electrophysiological signals are acquired through the fMMD wirelessly as shown in Fig. 5b (Case 1, multi-modal biosensing at a single spot). Figure 5c shows the various electrophysiological signals, including ECG, skin temperature, and body movement, through an electrical modality of the chest-mounted fMMD during desk work and walking. The folded structure and strain-isolating elastomer allow the fMMD to reliable measurements by ensuring intimate contact with the skin and reducing mechanical stress, allowing stable acquisition of signals such as ECG and 3-axis accelerometer data and tracking three-dimensional reconfigured motion even during activities like sitting-to-standing transitions, and walking (Supplementary Fig. 13). Supplementary Fig. 14 demonstrates the capability of Case 1 measurement to perform heart rate and heart rate variability (HRV) analysis during post-workout recovery. The gradual decrease in heart rate and the corresponding increase in HRV and the rate of HRV changes reflect the body’s shift to a relaxed state, indicating effective monitoring of autonomic function. This setup can be applied to cardiovascular health assessment and stress recovery monitoring in daily life. In addition, the wrist-mounted fMMD begins with setting the PWM to maximize the light intensity of the LD up to 5 mW aligned with a flexible waveguide, and the PD aligned with another waveguide measures the reflected light. The photocurrent is filtered and amplified for the output voltage to be within the optimal range for the analog to digital converter (ADC) of a microcontroller system. A measured PPG signal is transmitted to a personalized computer through a wireless communication module with a sampling frequency of 500 Hz. The synchronized ECG and PPG from the fMMDs on the chest and wrist enable the monitoring of pulse arrival time (PAT) as shown in Fig. 5d (Case 2, single modal biosensing at multi-spots), which can be used as a medical indicator for sleep quality, arterial stiffness, and myocardial ischemia^57–59^. Calculation of the time interval between peaks of ECG and PPG enable to acquire the PAT, as shown in Fig. 5e, f. Supplementary Fig. 15a, b show the capability of Case 2 measurement to monitor PAT changes before and after exercise, using synchronized ECG and PPG signals from the fMMDs on the chest and wrist. The average PAT decreases following exercise (239 ms **±** 2.9 ms) compared to desk work (291 ms **±** 4.7 ms), which aligns with increased cardiovascular activity and elevated blood pressure after physical exertion. This real-time monitoring of PAT highlights the device’s potential for blood pressure-related assessments and applications. Figure 5g presents the schematic showing a demonstration of real-time feedback operation based on electrical and chemical modalities (Case 3, multi-modal feedback operation at a single spot). To verify the feedback system, the fMMD is designed to transmit collected ECG signals to an external PC, where HR is calculated in real-time. When the HR exceeds 120 bpm, the system initiates a feedback algorithm that sends a packet to trigger drug delivery, ensuring the start of micropump operation (Fig. 5h and Supplementary Movie 2). It should be noted that in the demonstration shown in Supplementary Movie 2, no actual drug is being injected, and the video is intended to illustrate the system’s functionality. The feedback operation is verified through the infrared (IR) imaging of the microheater array as shown in Fig. 5i. The thermal images confirm that when drug delivery is triggered, the local heat generation does not affect the drug reservoir. Consequently, the proposed wearable platform, which enables simultaneous multiplexed biosensing and drug delivery as feedback can be utilized as a potential tool for automated cardiovascular monitoring.

## Discussion

Intelligent wearable medical devices for physiological biosensing and self-initiating therapy delivery are considered the ultimate goal for modern personal healthcare. However, challenges exist from various aspects, including a system-level configuration, drug dose management, and integration into a single device without compromising flexibility or stretchability^9^. In terms of device fabrication, both via and folding methods can be considered for implementing double-sided and multilayer structures. The via method facilitates vertical stacking, which can be advantageous for system integration and miniaturization; however, the structural variation required for different applications increases process complexity and can lead to alignment mismatches and potential damage when integrated with flexible components^37,60^. In contrast, the folding method may result in a slightly thicker device but is advantageous for integrating multiple modalities due to its lower process complexity. In addition, by incorporating a strain-isolating layer, the folding approach can prevent delamination from the skin caused by rigid components, making it more suitable for system-level multimodal devices. Accordingly, in this study, we introduce a unified platform by incorporating multiple modalities – electrical, optical, and chemical – with a folded structure, which enables multiplexed biosensing and on-demand drug delivery. We have designed functionally sectioned islands on an FPCB, connected by serpentine interconnects. This configuration not only ensures robustness against deformation but also allows for the integration of various components such as power management, communication modules, signal processing units, and drug loading chambers. By folding the serpentine FPCB and introducing a strain-isolating layer between the upper and lower layers, we have achieved strain robustness crucial for maintaining device integrity and performance during skin deformation. Drug delivery module based on the force-electrically driven micropump enables sustainable and controllable delivery with a flow rate of 4 ~ 12 nL/s. Theoretical and experimental analysis for design parameters and applied voltage have proved adjustable flow rates. The biosensing functionality of the fMMD has been demonstrated through real-time monitoring of hemodynamic and cardiovascular health states, using flexible waveguides for PPG monitoring and sensors for ECG monitoring. In addition, the proposed fMMD has proven its capability to derive a multiplexed factor, such as real-time PAT and HRV, in diverse physiological states and trigger the self-directed drug delivery based on the collected signal as a closed-loop system. In conclusion, our study represents a significant advancement in wearable healthcare technology, offering a closed-loop platform capable of intelligent monitoring and intervention. Leveraging multiplexed biosensing feedback, the drug delivery platform could be adapted for targeted blood pressure regulation through the administration of antihypertensive agents, such as sodium nitroprusside (SNP), enhancing its utility in managing cardiovascular conditions. Its potential applications extend beyond cardiovascular monitoring to include the management of blood glucose, pain, and various chronic diseases.

## Methods

### Circuit design for the fMMD

Overall FPCB designs using Altium Designer, for the fMMD with island-bridge structure. Electronic system involves A summary of the bill of materials for the fMMD involves power-management units for charging (MCP73832T-2ACI/OT, Microchip) and regulating (NCV8161ASN330T1G, Onsemi), Bluetooth module (FB300, Firmtech), microcontroller unit (STM32F405RGT6, STMicroelectronics), electrical modality for ECG sensing (AD8293G160ARJZ-R7, Analog Devices), accelerometer (ADXL337, Analog Devices) and skin temperature sensing (TMP6131QDECRQ1, Texas Instruments), optical modality for PPG monitoring (LD; chip-650-p5, Roithner LaserTechnik GmbH, PD; TEMD5010X01, Vishay, Signal processing; OPA2381AIDGKT, Texas Instruments), transistors (MMBT2222ALT1G, Onsemi) for microheater control of chemical modality, and passive components (capacitors, resistors; footprint in inch from 0402 to 0603). For the strain-isolating layer, a soft silicone elastomer (Ecoflex 10) was cast on a 3D printed mold, and the fMMD was fixed through thermal curing (70 °C in an oven for 30 min). bonding with the upper side and lower side of the fMMD using silicone adhesive finalized the folding process.

### Fabrication process of chemical module with peristaltic pump

Prepare four individual constituent parts: the substrate layer with a microheater, the pumping layer, the microfluidic layer; and the drug reservoir as follows. Depositing thin layers of metal by electron beam evaporation (Cr/Au; 7 nm/200 nm in thickness), and performing photolithography followed by wet etching defined serpentine traces on the polyimide substrate (100 μm in thickness) to prepare the substrate layer with the microheater. For preparing the pumping layer, photo-curable epoxy (SU-8 2150; Kayaku Advanced Materials, USA) was patterned on a silicon wafer to achieve mold-defining geometries through soft-lithography by allowing space (150 μm in height) for cavity volume expansion by the microheater. To facilitate the release of patterned PDMS from the mold during the soft-lithography process, the SU-8 mold was treated with the evaporated anti-stiction agent (trichloro(1H,1H,2H,-perfluorooctyl) silane, Sigma-Aldrich, USA) for 2 h at room temperature. Casting degassed 10:1 PDMS (base: curing agent) on the mold, followed by clamping with a glass slide (76 × 52 mm^2^) that was treated with Pt inhibitor solution composed of 5% AEAPS (3-(2-Aminoethylamino) Propylmethydimethoxysilane, Sigma-Aldrich, USA) and 95% methanol for 1 h. The PDMS pressed by the glass slide was cured for an hour at 70 °C in the oven and was delaminated from SU-8 mold after curing. For the microfluidic layer, processes are similar to those described in the fabrication of the pumping layer. Briefly explain, SU-8 photoresist was patterned on a silicon wafer to define micropump and microfluidic channel geometries of the microfluidic layer, followed by anti-stiction treatment as described above. Casting degassed 10:1 PDMS on the prepared mold and clamping the mold with prepared glass substrate treated with Pt inhibitor solution, followed by curing PDMS for an hour at 70 °C in the oven. The glass slide with the patterned microfluidic layer was detached from the mold. A puncher (1.2 mm ID) was used to make holes at the inlet and outlet of the micropump channel and microfluidic channel, respectively. Spin-casting and curing of flat, thin 10:1 PDMS layer prepared on a PI film (125 μm in thickness) to bond with patterned microfluidic layer via oxygen plasma treatment (100 W/20 mtorr/32 s/22 sccm O_2_ in O_2_ plasma system, Femto Science, Korea), which allow strong covalent bonding between microfluidic layer and prepared thin PDMS by activating their surfaces. For the drug reservoir, casting degassed 5:1 PDMS (base: curing agent) onto the 3D printed mold (Project 3500; 3D Systems, USA), followed by curing at 70 °C in the oven for about an hour. After completing the fabrication of the four individual parts for the microfluidic probe, strong siloxane bonding via oxygen plasma treatment (100 W/20 mtorr/32 s/22 sccm O_2_) allows the integration of micropump layer and microfluidic layer. The patterned drug reservoir was attached to the top part of the microfluidic layer where, covering the outlet of the micropump channel and inlet of microfluidic channel using silicone adhesive to avoid any leakages. The completed drug delivery module was attached to the MDD substrate using double-sided adhesive tape and electrically connected using conductive silver paste.

### Fabrication and assembly of microneedle array

The microneedle array was composed of a needle part and a base part. The needle part was formed on the thin base part, which is a PEG-DA film containing a microhole. To fabricate PEG-DA films with a microhole, PDMS pillar arrays (pillar diameter: 400 μm; height: 100 μm) were first prepared using the SU-8 mold fabrication and soft lithography process described previously in the fabrication of DDD. PEG-DA solution (Mn = 250; Sigma-Aldrich), mixed with 0.5% by weight of 2-hydroxy-2-methylpropiophenone (Sigma-Aldrich), a water-soluble photo-initiator, was then cast onto the fabricated PDMS pillar array. After covering it with glass, the mixture was exposed to UV (intensity: 20 mJ s^−1^; exposure time: 30 s). The crosslinked PEG-DA membrane was peeled off the PDMS pillar array to create a porous PEG-DA membrane. To fabricate an MN array on a PEG-DA membrane, a CNC mold with an MN structure (diameter: 300 μm; height: 700 μm) was fabricated, followed by creating a PDMS mold with an engraved needle pattern using the CNC mold. After precured PEG-DA was poured into the PDMS mold, the PEG-DA membrane was well aligned with the pattern and covered the PDMS mold. Excess PEG-DA solution was absorbed using a texwipe. The covered mixture was subjected to UV exposure, resulting in the microneedle array with a center microhole. The cured microneedle arrays were rinsed several times with ethanol and deionized water and then dried with a nitrogen gun to remove residual uncured PEG-DA.

The fabricated microneedle array was assembled with the microfluidic channel by surface functionalization. (3-aminopropyl)triethoxysilane (APTES) (Sigma-Aldrich) solution (5 volume % of APTES and 95 volume % of DI water) preheated at 80 °C in an oven. The backside of PEG-DA microneedle array was treated with oxygen plasma for 32 s (100 W/22 sccm). A droplet of APTES was uniformly applied to a microneedle array for 2 min, then dried with a nitrogen gun and dehydrated at 70 °C on the hotplate. Both of APTES-treated PEG-DA microneedle array and microfluidic channel were treated with oxygen plasma for 32 s (100 W/22 sccm) and then were bonded irreversibly at 70 °C on the hotplate for 30 min.

### Fluid flow rate measurement

The flow rate was measured by tracking diluted to distilled water. The fluid flowing inside the microfluidic channel was captured by a high-speed camera (Chronos 1.4, KRON Technologies) with a microscope lens at the frame rate of 100 frames per second. The video was analyzed by MATLAB with an open source code for HSV histogram to acquire the displacement in pixels in which the volumetric flow rate can be calculated by multiplying the cross-section area of the microfluidic channel with the fluid flow speed.

### Fabrication and assembly of optical module for PPG monitoring

For preparing the ridge-shaped monolithic PDMS waveguide, SU-8 2100 (Kayaku Advanced Materials, USA) was patterned on a silicon wafer to achieve engraved core geometries through soft-lithography. The patterned SU-8 mold was treated with an evaporated anti-stiction agent (trichloro(1H,1H,2H,-perfluorooctyl) silane, Sigma-Aldrich, USA) to facilitate the release of the patterned core structure from the mold for 2 h at room temperature. The surface of the glass slide was functionalized with Pt inhibitor solution composed of 5% AEAPS (3-(2-Aminoethylamino) Propylmethydimethoxy-silane, Sigma-Aldrich, USA) and 95% methanol for 1 h and then degassed 20:1 PDMS (base: curing agent) was spin-coated onto the modified glass slide at 300 rpm followed by curing for 30 min at 70 °C in the oven. Degassed 2.5:1 PDMS (base: curing agent) was poured onto the SU-8 mold, and then the mold was pressed by clamping with a PDMS-coated glass slide. The fabricated waveguide was cut into the desired shape using a laser blade, and the edge of the waveguide was cut at a 45-degree angle for perpendicular irradiation of light. The core of the waveguide and the beam spot of LD were aligned using a precision 3-axis stage and fixed with optical adhesive (NOA 61, Norland).

### Finite element analysis of fMMD’s mechanical stability

The strain during the folding and deformation processes of the fMMD was calculated using the numerical simulation tool COMSOL. As shown in Fig. 2a, the overall layout consists of an FPCB (Polyimide/Au/Polyimide; 100μm/15μm/100μm in thickness) connected by serpentine interconnects, modules, and a strain-isolating layer (Ecoflex; thickness 100 μm in thickness). To perform the finite element analysis (FEA) of mechanical stability, it was necessary to calculate the folding process of the fMMD and various deformation modes of the fMMD. The folding process was conducted by wrapping and pressing the substrate into a fully folded state, as depicted in Fig. 2c. Strain was analyzed at each stage of the folding process and various deformation modes. The devices with and without an encapsulation layer were laminated onto the skin and subjected to various deformations. The strain and stress of the devices were then analyzed under these skin deformations (stretching, bending, and twisting). After the analysis, the skin topography was post-processed to ensure it was not visible. The mesh of the interconnect and the strain-isolating layer was formed with high density to ensure calculation accuracy. The material parameters used for FEA (density, Young’s modulus, Poisson’s ratio) are listed in Supplementary Table 2.

### Finite element analysis of fMMD’s fluidic stability

The folded configuration, pressure, and injection volume of the micropump in the foldable fMMD were also calculated using the numerical simulation tool COMSOL. As shown in Fig. 3a, the micropump consists of a heater, pump, and microchannel. For the folded configuration of the micropump, calculations were performed as depicted in Fig. 3c. Subsequently, to calculate the pressure, both the individual operation of components and their interactions need to be considered. The voltage sequentially applied to the heaters (Cr/Au; thickness 7 nm/200 nm) induced temperature variations in each heater due to Joule heating. Convective heat flux occurred between the device and the ambient air (20 °C), resulting in a temperature decrease in the device. The heat generated by the micro heater was conducted to the air inside the cavity. The air pressure inside the cavity was applied to the walls of the internal boundary containing the peristaltic membrane. The pressure was calculated using the ideal gas law. The calculated pressure was then applied to the microchannel of both the initial and folded configurations to determine the injection volume. To enhance calculation accuracy, the mesh at the interface between the channel and the fluid was formed with high density. The material parameters used for FEA (density, Young’s modulus, Poisson’s ratio) are listed in Supplementary Table 2.

### Finite element analysis of foldable fMMD’s waveguiding

To analyze the guided intensity of light through the waveguide and the scattering of the incident beam on the skin, numerical simulations were performed using COMSOL. As shown in Fig. 4d, a Gaussian-distributed laser beam (5 mW) was incident perpendicularly onto the waveguide in both folded and unfolded configurations. The total internal reflection within the waveguide and the light distribution reaching the upper surface of the skin was calculated using the Ray Optics module. Additionally, the Heat Transfer module was utilized to compute the scattering distribution of the light that reached the upper surface of a segment of skin tissue (6.4 mm × 3.1 mm × 3 mm) at the end of the waveguide. The material parameters used for FEA (refractive index, Absorption coefficient, reduced scattering coefficient of skin tissue, anisotropy Factor) are listed in Supplementary Table 2.

### Data analysis and algorithms for multiplexed measurement and closed loop operation

For real-time measurement, the fMMDs were attached to the human subject’s chest and wrist, respectively. The chest unit measured the ECG, skin temperature, and acceleration at a sampling rate of 500 Hz, and the wrist unit operated the PWM to trigger LD and measured the PPG signal through a series of analog signal processing, including analog filters inverting amplifiers. The collected signals were digitized with 32-bit ADC and transmitted to the personal laptop. Signal processing used a detrend algorithm to remove baseline wander using analysis software (MATLAB R2023b). The peak detection algorithm acquired peak-to-peak time intervals for ECG and PPG signals. PAT was calculated from the time intervals between simultaneously obtained ECG and PPG signal waveforms. In addition, the collected ECG signal from the chest unit was used to calculate heart rate in real-time, utilizing a window size of 5 s. For HRV calculation, a sliding window with a size of 5 s and an overlap of 1 s was applied to the ECG signal, using the intervals between peaks to compute HRV. The Root Mean Square of Successive Differences (RMSSD) was employed as the HRV metric, as it is particularly sensitive to high-frequency variations and provides an effective measure of parasympathetic nervous system activity, making it well-suited for assessing short-term changes in autonomic function. The data packet to initiate drug delivery was transmitted wirelessly from MATLAB to the fMMD when the calculated heart rate exceeded 120 bpm as a way to show a closed-loop system. The feedback-actuated drug delivery module delivered a sham drug in a reservoir with a switching frequency of 1 Hz, and the micropump behavior was filmed using an IR thermal camera.

### Ethics information for human subject research

The human subject volunteer was informed of the on-body experiment details and asked to sign the consent. A participant was male and age was 29 years old. The authors affirm that human research participants provided informed consent for the publication of the images in Figs. 1e, 2b, and Supplementary Movie 2.

### Reporting summary

Further information on research design is available in the Nature Portfolio Reporting Summary linked to this article.

## Supplementary information

Supplementary Information

Description of Additional Supplementary Files

Supplementary Movie 1

Supplementary Movie 2

Reporting Summary

Transparent Peer Review file

## Source data

Source Data

## Data Availability

All data supporting the findings of this study are available within the article and its supplementary files. Any additional requests for information can be directed to and will be fulfilled by, the corresponding authors. Source data are provided in this paper.

## Code availability

The custom codes used in this study for wireless electrophysiology monitoring and signal analysis are available from the corresponding author upon request.
