# Supplementary material for: Vialess heterogeneous skin patch for multimodal monitoring and stimulation
Source: Nat Commun. 2025 Jan 14;16:650. doi: 10.1038/s41467-025-55951-6 (PMC11733152; doi:10.1038/s41467-025-55951-6)
Supplement: Supplementary file 1 — Supplementary Information [file 41467_2025_55951_MOESM1_ESM.pdf]

Supplementary Information

Vialess heterogeneous skin patch for multimodal monitoring and stimulation

Hyeokjun Lee[1,7], Soojeong Song[1,7], Junwoo Yea[1], Jeongdae Ha[1], Saehyuck Oh[1], Janghwan Jekal[1], Myung Seok Hong[2], Chihyeong Won[3], Han Hee Jung[4], Hohyun Keum[5], Sangyoon Han[1], Jeong Ho Cho[6], Taeyoon Lee[3], and Kyung-In Jang[1,*]

Affiliations

[1]Department of Robotics and Mechatronics Engineering, Daegu Gyeongbuk Institute of Science and Technology (*DGIST*), Daegu 42988, Republic of Korea

[2]ASML Korea, Hwaseong, 18449, Republic of Korea.

[3]School of Electrical and Electronic Engineering, Yonsei University, Seoul, 03722, Republic of Korea.

[4]Department of Information and Communication Engineering, Hannam University, Daejeon, 34430, Republic of Korea.

[5]Industrial Transformation Technology Department, Korea Institute of Industrial Technology (KITECH), Cheonan, 31056, Republic of Korea.

[6]Department of Chemical and Biomolecular Engineering, Yonsei University, Seoul, 03722, Republic of Korea.

[7]These authors contributed equally to this work.

[*]Corresponding author. E-mail: kijang@dgist.ac.kr (K.-I.J.)

Table of Contents

Supplementary Table 1 | Comparative analysis of fMMD and previously reported wearable devices ··· 2

Supplementary Table 2 | Simulation Parameters for mechanical, fluidic, and optical analysis ··· 3

Supplementary Fig. 1 | Photos of the fabricated fMMD ··· 4

Supplementary Fig. 2 | Mechanical analysis for design optimization of the folded MMD ··· 5

Supplementary Fig. 3 | Mechanical Properties of the fMMD with encapsulation layer ··· 6

Supplementary Fig. 4 | Fabrication process of PEGDA microneedle array ··· 7

Supplementary Fig. 5 | Optical and SEM images of PEGDA based microneedle ··· 8

Supplementary Fig. 6 | Validation of microneedle array for skin penetration ··· 9

Supplementary Fig. 7 | The temperature change of microheater array during pneumatic actuation 10

Supplementary Fig. 8 | Experimental set-up for flow rate measurement of drug delivery module · 11

Supplementary Fig. 9 | Various patterns of flexible monolithic PDMS waveguide ··· 12

Supplementary Fig. 10 | Fabrication process of PDMS waveguides with ridge structure ··· 13

Supplementary Fig. 11 | Transmittance comparison of flexible PDMS waveguide under mechanical deformation using finite element analysis (FEA) ··· 14

Supplementary Fig. 12 | Characterization of optical losses for flexible waveguides in terms of propagation, stretching, and out-of-plane bending ··· 15

Supplementary Fig. 13 | Electrical reliability of biosensing under diverse motion states ··· 16

Supplementary Fig. 14 | Real-time measurement of heart rate and resulting heart rate variability during post-workout recovery ··· 17

Supplementary Fig. 15 | Comparison of changes in pulse arrival time over time before and after exercise ··· 18

Supplementary References ··· 19

Type	Biosensing modality	Stimulation modality	Multi-layer method	System-level	Ref
Stretchable	Electrical, Optical	Chemical	Fold	Standalone	This work
Stretchable	Optical	X	X	Patch type (connected to an evaluation module)	1
Flexible	Chemical	X	X	Standalone	2
Stretchable	Electrical, Chemical	Chemical	X	Patch modules (connected to a portable analyzer)	3
Stretchable	X	Chemical	X	Stretch-responsive patch module	4
Flexible	X	Chemical	X	Microneedle patch	5
Rigid	Electrical, Optical	X	Via-hole (Wire soldering)	Standalone	6
Flexible	Electrical, Optical	X	Via-hole (Conductive tape)	Patch type (connected to a rigid PCB)	7
Stretchable	Electrical, Optical	X	Via-hole (Conductive tape)	External reader (NFC)	8
Stretchable	Electrical	X	Via-hole (Conductive Ag paste)	Standalone	9
Stretchable	Electrical	X	Via-hole (Conductive filling)	Standalone	10
Flexible	X	Chemical	Via-hole (drug delivery port)	External source to trigger drug delivery	11
Flexible	Electrical	Chemical	Via-hole (drug delivery port)	External reader, External force to trigger drug delivery	12
Flexible	Electrical, Chemical	Electrical, Optical	Fold (Electrical connector)	Standalone	13
Stretchable	Electrical	X	Fold (Serpentine interconnect)	Standalone	14
Stretchable	Electrical	X	Fold (Electrical connector)	Standalone	15
Stretchable	Electrical, Optical	X	Fold (Serpentine interconnect)	Standalone	16

Supplementary Table 1 | Comparative analysis of fMMD and previously reported wearable devices.

Parameters	Units	Value
Density of Ecoflex	kg/m^3	1040
Young's modulus of Ecoflex	kPa	50
Poisson ratio of Ecoflex		0.49
Density of polyimide	kg/m^3	1300
Young's modulus of polyimide	kPa	3.1e6
Poisson ratio of polyimide		0.34
Density of Au	kg/m^3	19300
Young's modulus of Au	kPa	70e6
Poisson ratio of Au		1
Refractive index of Core PDMS		1.42
Refractive index of Cladding PDMS		1.405
Refractive index of air		1
Refractive index of skin tissue (650 nm)		1.5
Absorption coefficient of skin tissue (650 nm)	mm^{-1}	0.066
Reduced scattering coefficient of skin tissue (650 nm)	mm^{-1}	1.68
Anisotropy Factor of skin tissue (650 nm)		0.8

Supplementary Table 2 | Simulation Parameters for mechanical, fluidic, and optical analysis.

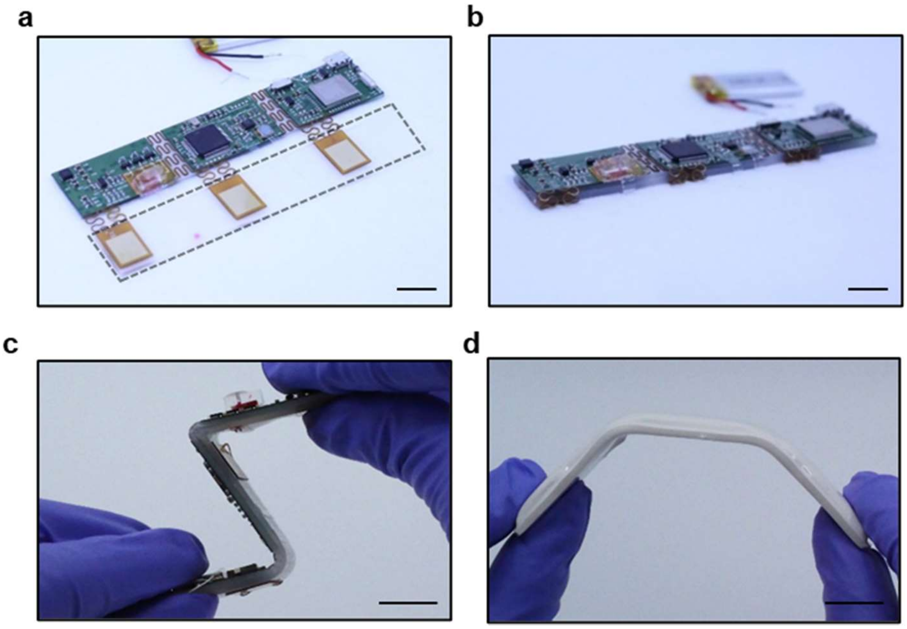

Supplementary Fig. 1 | Photos of the fabricated fMMD. (a, b) A photo of integrated fMMD mounted on strain-isolating elastomer before folding. Scale bar, 1 cm. **(c)** A photo of integrated fMMD with z-shape induced by island-bridge structure. Scale bar, 1 cm. **(d)** A photo of bent state of fully encapsulated fMMD. Scale bar, 1 cm.

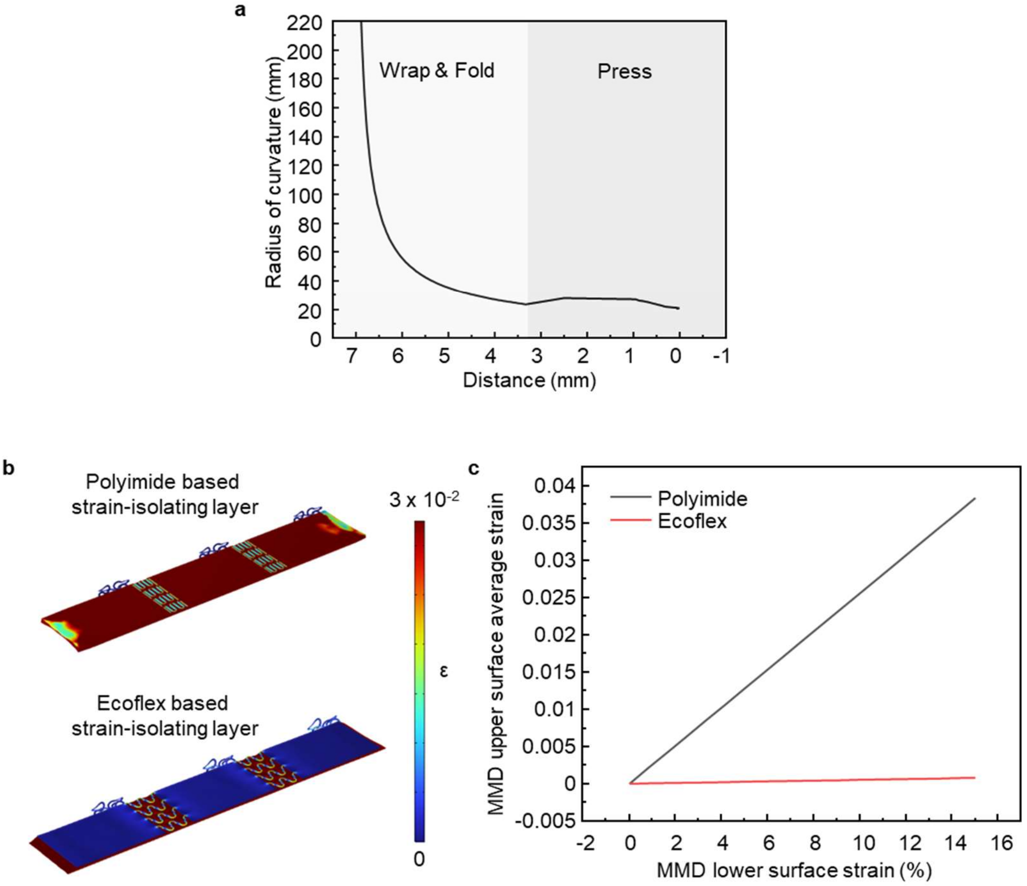

Supplementary Fig. 2 | Mechanical analysis for design optimization of the folded MMD. (a) Graph of radius of curvature versus distance during the folding process of the substrate for the straight line interconnect design. The x-axis represents the distance (mm) between the skin contact side and the non-contact side, while the y-axis indicates the radius of curvature (mm) of the straight interconnect. **(b)** Mechanical analysis of the principal strain under 15% stretching for entire MMD with polyimide-based versus ecoflex-based strain-isolating layers. **(c)** Analysis of the average strain applied to the non-contact side of the substrate based on the strain at the MMD lower surface, from a simulation study comparing the strain isolating effects of materials with different Young's modulus (Polyimide and Ecoflex).

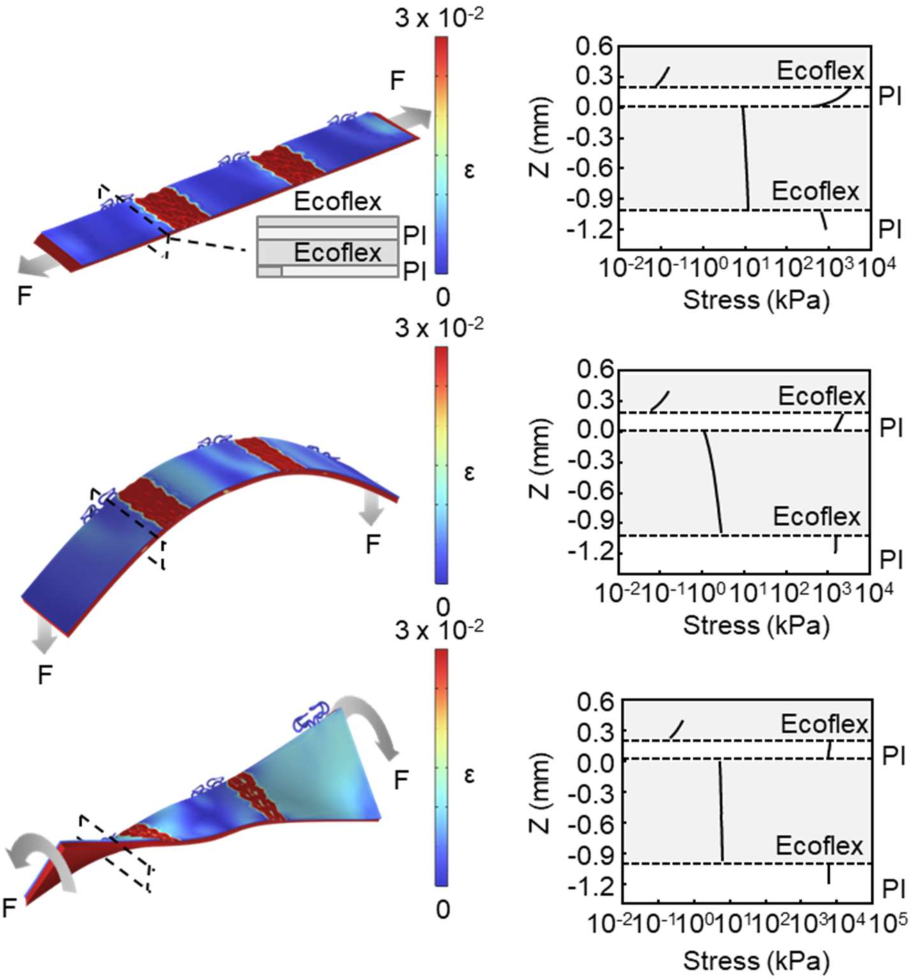

Supplementary Fig. 3 | Mechanical Properties of the fMMD with encapsulation layer. Strain and stress of fMMD with encapsulation layer under multi-deformation modes (stretching, bending, twisting). The strain-isolating effect is demonstrated for deformations applied to the skin-interfaced layer, as shown by stress analysis on a cross-sectional plane (indicated by the black dashed box).

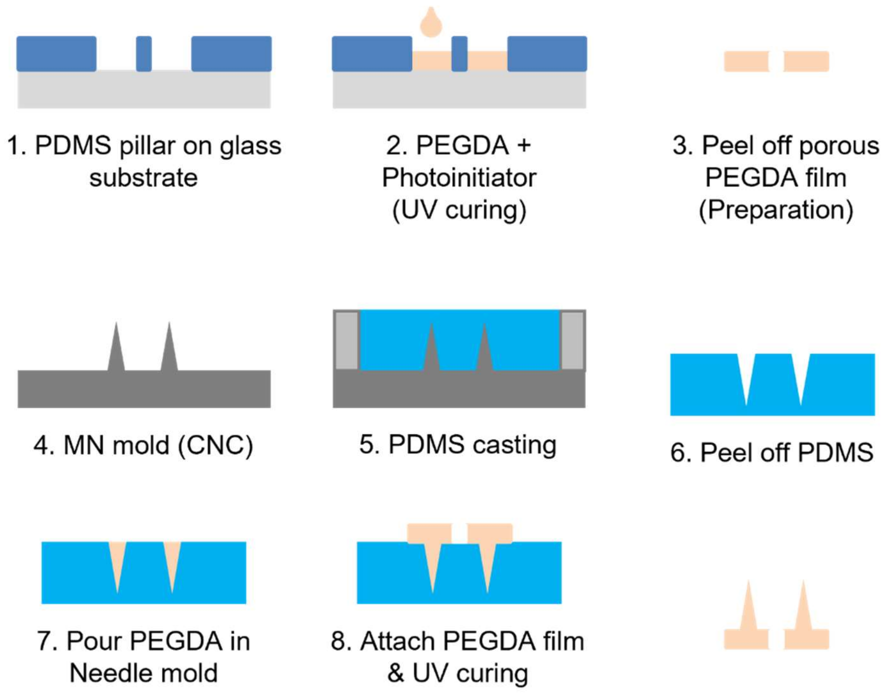

Supplementary Fig. 4 | Fabrication process of PEGDA microneedle array.

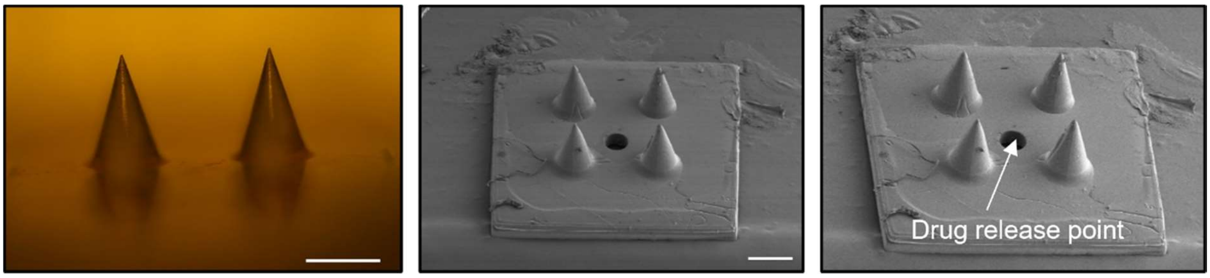

Supplementary Fig. 5 | Optical and SEM images of PEGDA based microneedle. Scale bar, 500 μm.

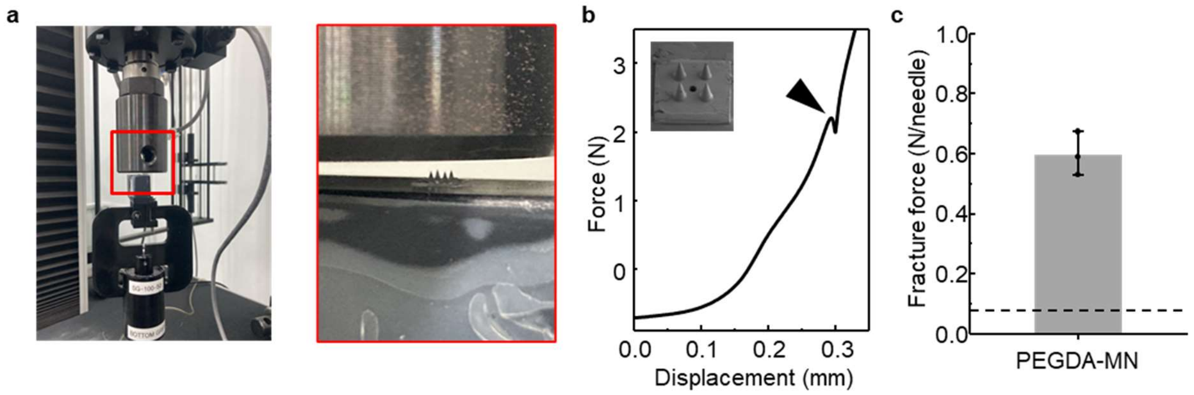

Supplementary Fig. 6 | Validation of microneedle array for skin penetration. (a) Experimental setup for measurement of fracture force. **(b)** Experimental result for measurement of force required to fracture the 2 x 2 microneedle array. Fracture point shows the fluctuation of force applied to load cell. **(c)** Analysis of axial load fracture force per needle. The measured force is 0.60 N (n = 3 samples), which exceeds the minimum requirement for skin insertion. Error bars represent the standard deviation

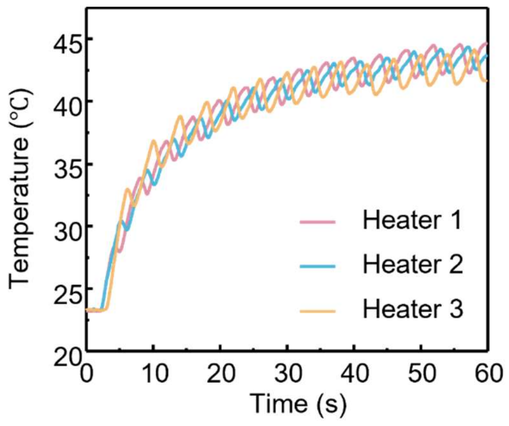

Supplementary Fig. 7 | The temperature change of microheater array during pneumatic actuation.

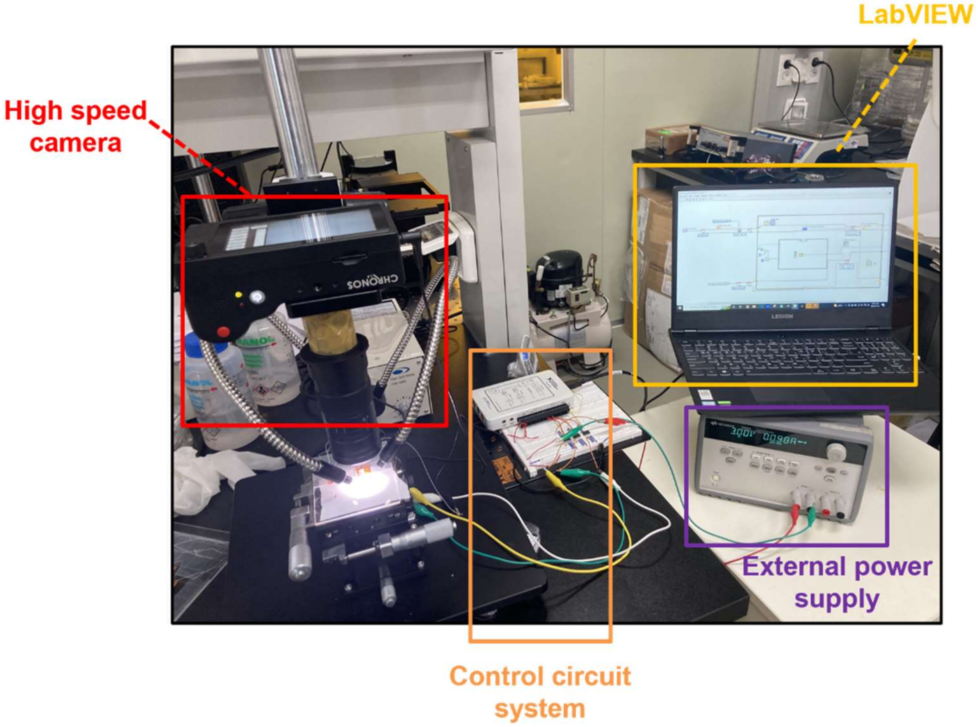

Supplementary Fig. 8 | Experimental set-up for flow rate measurement of drug delivery module.

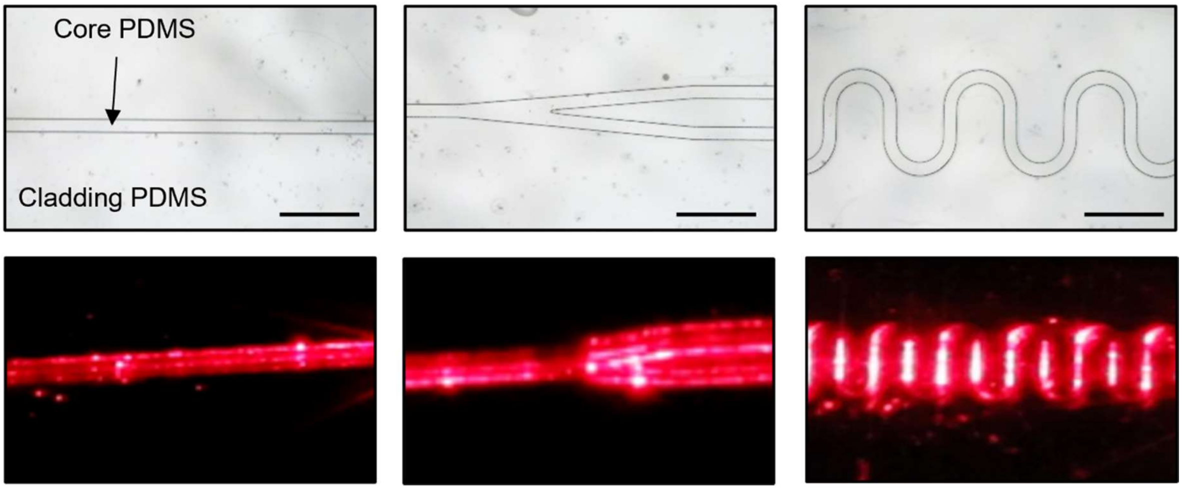

Supplementary Fig. 9 | Various patterns of flexible monolithic PDMS waveguide (straight, split, and serpentine line, Scale bar, 500 μm).

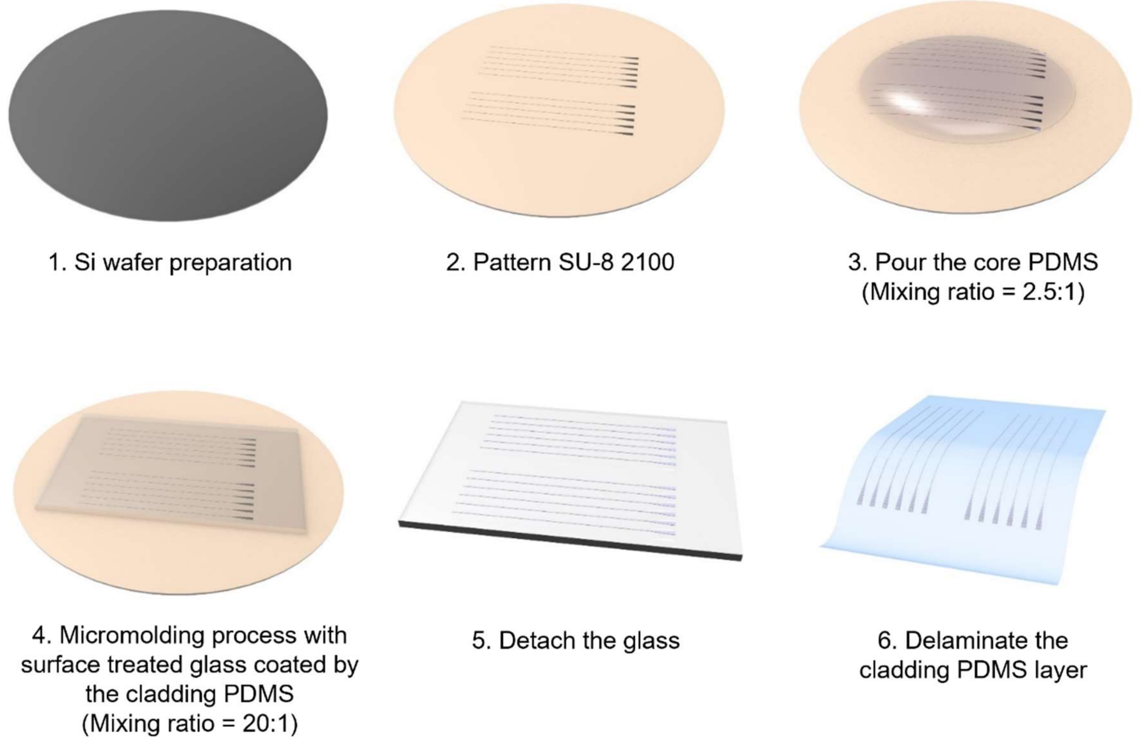

Supplementary Fig. 10 | Fabrication process of PDMS waveguides with ridge structure.

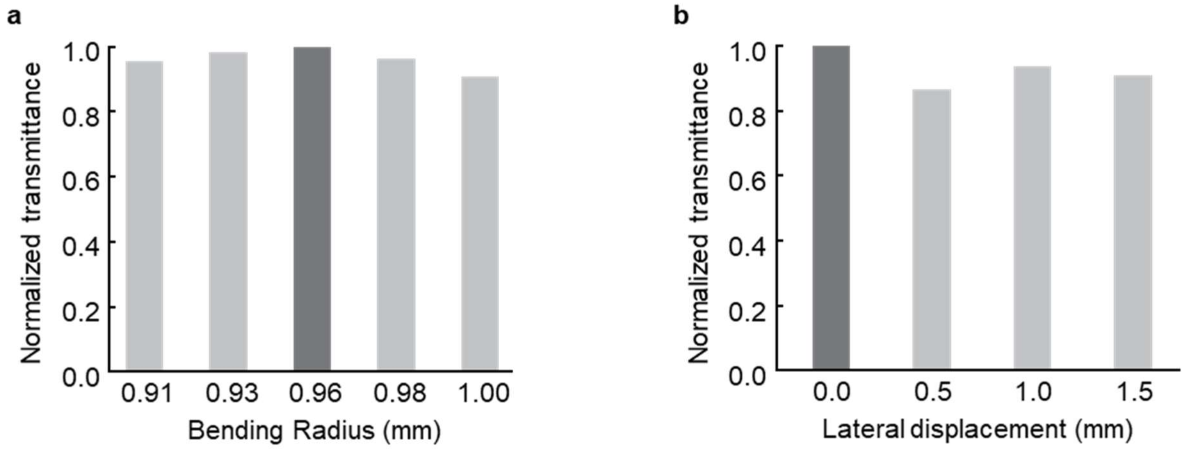

Supplementary Fig. 11 | Transmittance comparison of flexible PDMS waveguide under mechanical deformation using finite element analysis (FEA). (a) Normalized transmittance versus bending radius of flexible PDMS waveguide. A dark gray box represents the undeformed state of the folded device. **(b)** Normalized transmittance versus lateral displacement of flexible PDMS waveguide due to shearing deformation. The waveguide attached to the skin is laterally displaced relative to the waveguide connected to the laser. A dark gray box represents the undeformed state of the folded device.

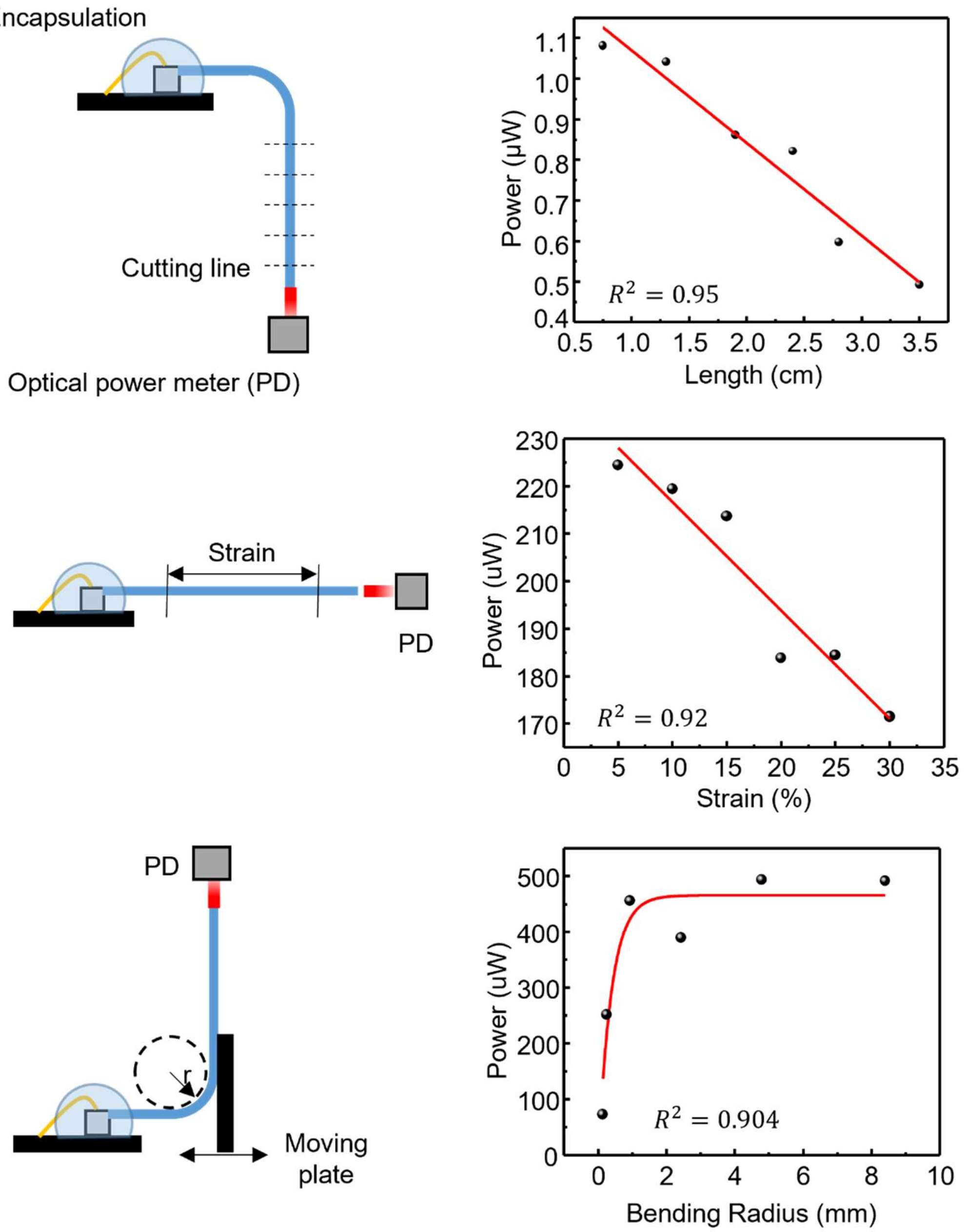

Supplementary Fig. 12 | Characterization of optical losses for flexible waveguides in terms of propagation, stretching, and out-of-plane bending.

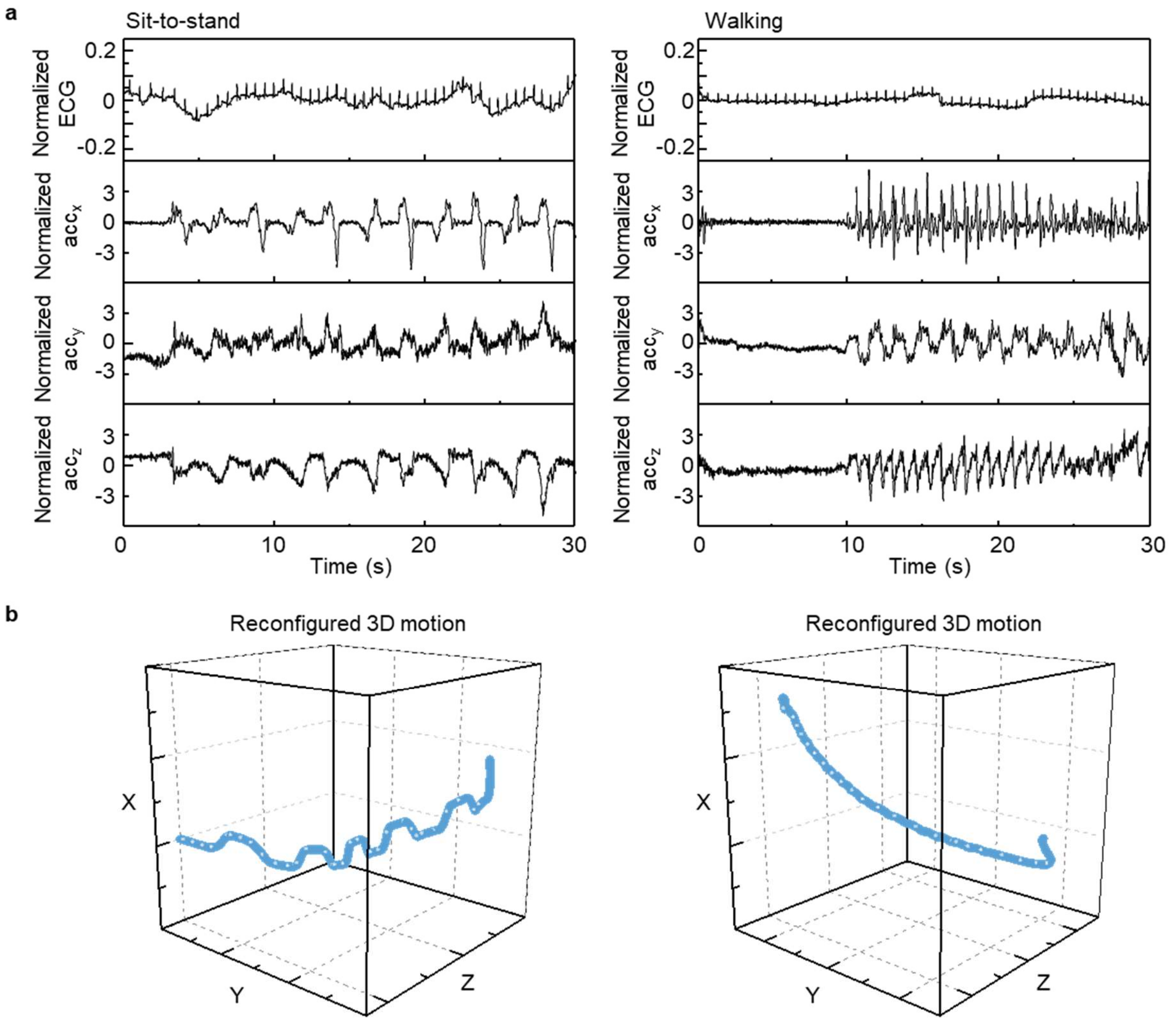

Supplementary Fig. 13 | Electrical reliability of biosensing under diverse motion states. (a) Representative recording of ECG and three-axis acceleration from fMMD attached on the wearer's chest for cases of sit-to-stand and walking. **(b)** Inferred 3D patterns of motion according to measured accelerations.

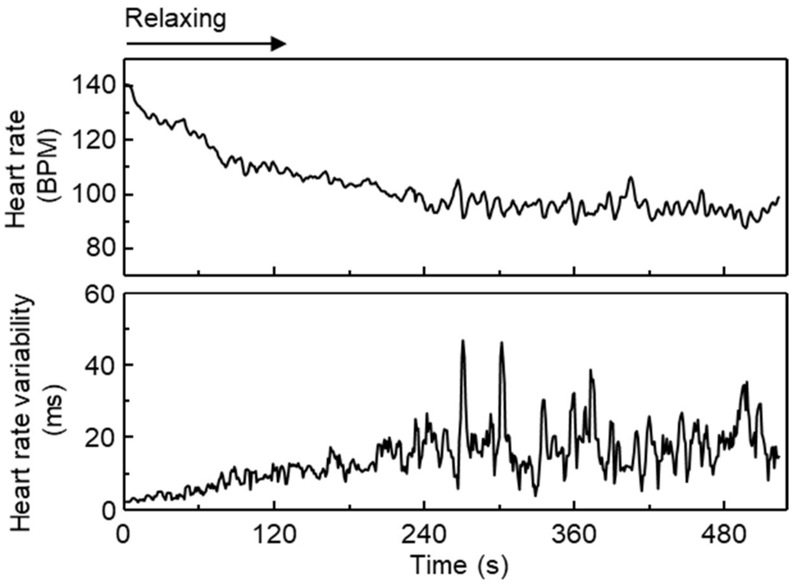

Supplementary Fig. 14 | Real-time measurement of heart rate and resulting heart rate variability during post-workout recovery.

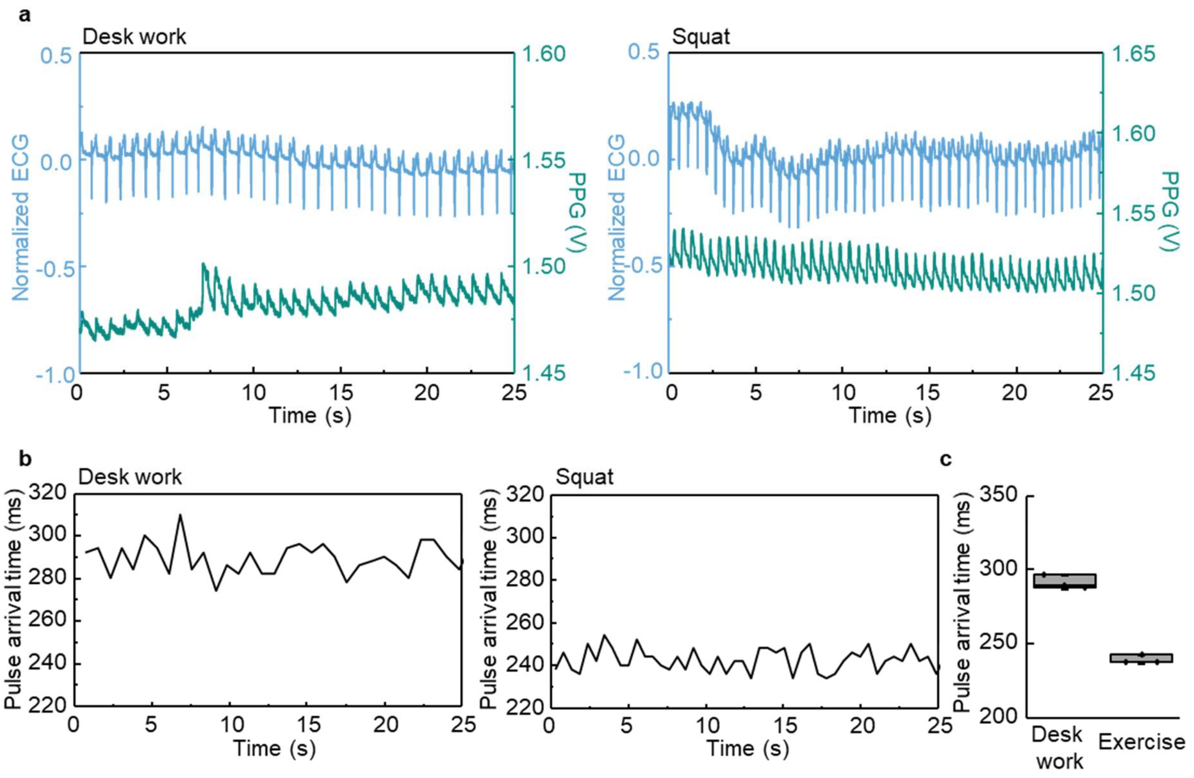

Supplementary Fig. 15 | Comparison of changes in pulse arrival time over time before and after exercise. (a) Real-time measurement with optical and electrical modalities during desk work and after squat workout. **(b)** Calculation of PAT over time for two activities (desk work and exercise). **(c)** Comparison of average PAT for measuring time (n = 3 measurements). Error bars represent the standard deviation.

Supplementary References

1. Lee, G. H., et al. Stretchable PPG sensor with light polarization for physical activity–permissible monitoring. *Science Advances*. **8**, eabm3622. (2022)
2. Gao, W., et al. Fully integrated wearable sensor arrays for multiplexed in situ perspiration analysis. *Nature*. **529**, 509-514. (2016)
3. Lee, H., et al. Wearable/disposable sweat-based glucose monitoring device with multistage transdermal drug delivery module. *Science advances*. **3**, e1601314. (2017)
4. Di, J., et al. Stretch-triggered drug delivery from wearable elastomer films containing therapeutic depots. *ACS nano*. **9**, 9407-9415. (2015)
5. Bae, W. G., et al. Snake fang–inspired stamping patch for transdermal delivery of liquid formulations. *Science Translational Medicine*. **11**, eaaw3329. (2019)
6. Choi, M., et al. Wearable device-based system to monitor a driver's stress, fatigue, and drowsiness. *IEEE Transactions on Instrumentation and Measurement*. **67**, 634-645. (2017)
7. Bartlett, M. D., et al. Rapid fabrication of soft, multilayered electronics for wearable biomonitoring. *Advanced Functional Materials*. **26**, 8496-8504. (2016)
8. Jeong, H., et al. Modular and reconfigurable wireless e-tattoos for personalized sensing. *Advanced Materials Technologies*. **4**, 1900117. (2019)
9. Zhao, D., et al. Flexible hybrid integration enabled xsOn-skin electronics for wireless monitoring of electrophysiology and motion. *IEEE Transactions on Biomedical Engineering*. **69**, 1340-1348. (2021)
10. Huang, Z., et al. Three-dimensional integrated stretchable electronics. *Nature electronics*. **1**, 473-480. (2018)
11. Lee, S. Y., et al. Combinatorial wound healing therapy using adhesive nanofibrous membrane equipped with wearable LED patches for photobiomodulation. *Science advances*. **8**, eabn1646. (2022)
12. Honda, W., et al. Wearable, human-interactive, health-monitoring, wireless devices fabricated by macroscale printing techniques. *Advanced Functional Materials*. **24**, 3299-3304. (2014)
13. Yang, S. M., et al. Soft, wireless electronic dressing system for wound analysis and biophysical therapy. *Nano Today*. **47**, 101685. (2022)
14. Jeong, H., et al. Differential cardiopulmonary monitoring system for artifact-canceled physiological tracking of athletes, workers, and COVID-19 patients. *Science advances*. **7**, eabg3092. (2021)
15. Kim, H., et al. Fully integrated, stretchable, wireless skin-conformal bioelectronics for continuous stress monitoring in daily life. *Advanced Science*. **7**, 2000810. (2020)
16. Chung, H. U., et al. Skin-interfaced biosensors for advanced wireless physiological monitoring in neonatal and pediatric intensive-care units. *Nature medicine*. **26**, 418-429. (2020)
