# Supplementary material for: Vialess heterogeneous skin patch for multimodal monitoring and stimulation
Source: Nat Commun. 2025 Jan 14;16:650. doi: 10.1038/s41467-025-55951-6 (PMC11733152; doi:10.1038/s41467-025-55951-6)
Supplement: Supplementary file 2 — Description of Additional Supplementary Files [file 41467_2025_55951_MOESM2_ESM.pdf]

<u>Description of Additional Supplementary Files</u>

Supplementary Movie 1. Thermo-pneumatic micropumpbased drug delivery module demonstration.

Supplementary Movie 2. Closed loop system demonstration.
