# Supplementary material for: Vialess heterogeneous skin patch for multimodal monitoring and stimulation
Source: Nat Commun. 2025 Jan 14;16:650. doi: 10.1038/s41467-025-55951-6 (PMC11733152; doi:10.1038/s41467-025-55951-6)
Supplement: Supplementary file 6 — Transparent Peer Review file [file 41467_2025_55951_MOESM6_ESM.pdf]

nature portfolio

Peer Review File

Vialess heterogeneous skin patch for multimodal monitoring and stimulation

Corresponding Author: Professor Kyung-In Jang

This file contains all reviewer reports in order by version, followed by all author rebuttals in order by version.

Version 0:

Reviewer comments:

Reviewer #2

(Remarks to the Author)
This authors present a method for multimodal skin patch integration, which is folding to connect the rigid electronic components and the soft skin. The method is simple but effective, and the study of the effect of folding is comprehensive from both simulation and experiment on mechanical, electrical, optical, and microfluidic aspects. The sensing-stimulated drug delivery device is well-designed and has large application potential. The manuscript is well organized and the expression in both text and figures are clear. However, some of the application demonstrations still require more in-depth and detailed discussion and explanation, and the following points should be addressed before being considered for publication.

1. Page 4 line 83, the authors stated that the via-hole process has been predominantly used. A few references on the via-hole connected biomedical devices should be provided.
2. What is the necessity of the folding? A single layer with a soft substrate without folding can also realize the functions. It is important to clarify this point in the manuscript to strengthen the creative point.
3. This work demonstrates the superiority of folded structures for integrating multifunctional components from a mechanical stability perspective using finite element analysis, but does not discuss the electrical reliability of biosensing and signal feedback under diverse motion states, more electrical characterization (sensitivity and operation stability for practical applications, etc.) should be added.
4. It is suggested to briefly discuss whether the via-hole process is applied for electrical connection, mechanical alignment, optical pathway, or fluid channel. It is confusing to merge all these functions, since in Line 86, the conductive paste is applied in the via-hole, and line 87 states the leakage of drug fluid due to misalignment.
5. In Figure 1a, the electrical modules cannot match the 5 modules in the main content description. For example, we cannot spot the battery and BLE module in the figure. Similar problem is observed in Figure 1b, where the position LD, waveguide, and photodetector are not clear. Maybe the author can label ① ② ③ according to the description in the main content if the space not enough to label the full names in the figure.
6. In Figure S2a, the unit seems to be wrong. Curvature radius equals to the reciprocal of curvature, where the unit for curvature radius is mm, and the unit for curvature is mm-1.
7. The word “polymer gel” is not correct to describe the ecoFlex in the strain-isolating layer. Polymer gel usually indicates a semisolid system in a liquid medium (such as swollen by a solvent).
8. The heterogeneous integration is important, in which the bonding between soft and rigid surfaces should be better studied. The FEA study in Figure 2f shows the function of the strain isolating layer, but I feel it is not enough. Could the author add a FEA study to show the strain in the heterogeneous device when attached to the skin? e.g. including rigid layer, strain isolating layer, skin, and probably the encapsulation layer. Displacement might be assigned from the skin side, and study the stress and strain on each layer of the device.
9. Since the whole device is encapsulated with a white color polymer, how can we determine when the drug has been used up? And how to top up the drug?
10. The authors utilize a folded structure with serpentine interconnections to guarantee seamless integration of multiple sensing modalities for real multi-pathway biosensing and on-demand drug delivery. However, a great deal of real-time monitoring of biosignals and qualitative analysis is absent in practical applications. Possible solutions to achieve a better design of the sensing-delivery-feedback platform to tackle the point should be provided and discussed.

Reviewer #3

(Remarks to the Author)
In this manuscript, the authors describe a multilayered, flexible, skin-mounted device made by folding an electronic circuit board. In addition to electrical circuits, they show that folding can be applied to chemical and optical modalities, and demonstrate a functional device by acquiring PPG, ECG, and temperature on a single subject. While the idea of folding multilayered circuit boards is simple and has been used in prior work, the extension to other modalities in a functional device is interesting and useful. I found the manuscript to be written and presented clearly. My specific comments are below:

1. Introduction: "However, this method requires complex procedures which are individually fabricating each single layer with via-holes, precisely aligning them, and connecting with conductive tape or paste."

a. Vias are a well developed technology in the PCB industry and can be reliably mass produced even on very thin PCBs. While I understand that standard industry processes cannot be used to produce stacked circuit layers that retain the flexibility of a single layer, it seems that they could be used to produce the double-sided circuit boards considered here. Could the authors clarify this statement, or focus more on the need for multimodal connectivity between layers rather than on the disadvantages of vias?

b. Following the above, the relative advantages and disadvantages of folding and vias should be discussed in a more nuanced manner in the Discussion section. I do not see folding as the superior choice to vias in many situations, including when many inter-layer connections are needed, the total thickness needs to be small, and there are limited placement options near the edges.

2. Can the authors provide actual images of the folded regions in Fig. 1 and 2 in addition to the illustrations?

3. It is unclear from Fig. 1 how the device is powered as the block diagram simply indicates "power" and the LiPo battery is not shown in the illustration.

4. Could the authors add references at the end of this sentence? "As an alternative to via-hole process, a method has been developed that 89 fabricates overall systems in a single layer and then folds them at once." (Line 88)

5. For the PPG sensor design in Fig. 4, there is potential concern that movement changes in the bending radius and introduces artifacts in the amplitude profile of the signal.

6. Fig. 5. Was a pharmaceutically active drug actually delivered to the subject? If so, is there a measured effect? If not, it should be clear in the caption/Methods section that this is a sham drug.

Version 1:

Reviewer comments:

Reviewer #2

(Remarks to the Author)
The authors have well addressed my comments, the revised manuscript is improved significantly. It is acceptable for publication.

Reviewer #3

(Remarks to the Author)
The authors have satisfactorily addressed all of my questions.

Open Access This Peer Review File is licensed under a Creative Commons Attribution 4.0 International License, which permits use, sharing, adaptation, distribution and reproduction in any medium or format, as long as you give appropriate credit to the original author(s) and the source, provide a link to the Creative Commons license, and indicate if changes were made.
In cases where reviewers are anonymous, credit should be given to 'Anonymous Referee' and the source.
The images or other third party material in this Peer Review File are included in the article's Creative Commons license, unless indicated otherwise in a credit line to the material. If material is not included in the article's Creative Commons license and your intended use is not permitted by statutory regulation or exceeds the permitted use, you will need to obtain permission directly from the copyright holder.
To view a copy of this license, visit https://creativecommons.org/licenses/by/4.0/

Reviewer# 2

This authors present a method for multimodal skin patch integration, which is folding to connect the rigid electronic components and the soft skin. The method is simple but effective, and the study of the effect of folding is comprehensive from both simulation and experiment on mechanical, electrical, optical, and microfluidic aspects. The sensing-stimulated drug delivery device is well-designed and has large application potential. The manuscript is well organized and the expression in both text and figures are clear. However, some of the application demonstrations still require more in-depth and detailed discussion and explanation, and the following points should be addressed before being considered for publication.

Our response:

We sincerely appreciate the reviewer's careful evaluation of our manuscript. Your recognition of the significance of our research and your willingness to provide constructive feedback are highly valued. We have thought to do our best to address the questions you have raised and make the necessary revisions to ensure the quality and completeness of our work before resubmitting the manuscript. Your insights will undoubtedly contribute to further improving and strengthening our research. Thank you for your time and attention to our work, and the technical questions have been addressed in the following discussions.

Comment #1. Page 4 line 83, the authors stated that the via-hole process has been predominantly used. A few references on the via-hole connected biomedical devices should be provided.

Our Response:

We are grateful for the suggestion you have provided to enhance the quality of the manuscript. The via-hole process has been widely used not only in single-module devices, such as displays and epidermal sensors, but also in system-level circuits to create double-sided or multilayered structures. This process involves several techniques, including manually drilling holes and printing conductive materials around them, or layering dielectric materials or substrates, covering conductive traces while leaving small holes, and then printing additional conductive layers around the via-holes. In particular, the stacking of system-level circuits through via-holes or vertical interconnect access (VIA) has been extensively reported in the past five years. In response to your suggestion, we have added the recent works as references related with via-hole process for double-sided or multilayered structure as follows:

Related references

1. Huang, Z. et al. Three-dimensional integrated stretchable electronics. *Nat. Electron.* **1**, 473-480 (2018)
2. Song, H. et al. Highly-integrated, miniaturized, stretchable electronic systems based on stacked multilayer network materials. *Sci. Adv.* **8**, eabm3785 (2022)
3. Li, J. et al. Vertically stacked skin-like active-matrix display with ultrahigh aperture ratio. *Light. Sci. Appl.* **13**, 177 (2024)
4. Zhang, T. et al. A new printed electronics approach eliminating redundant fabrication process of vertical interconnect accesses: building multilayered circuits in porous materials. *Adv. Mater. Technol.* **3**, 4 (2018)

Our modification to the manuscript:

In Page 4, we have updated the text:

As one of the integration methods, stacking through via-hole processes have been predominantly used[29-32]. Reliable methods for via-hole fabrication and filling have been well-established for conventional electronics;

In Page 29, we have added the references:

29. Huang, Z. et al. Three-dimensional integrated stretchable electronics. *Nat. Electron.* **1**, 473-480 (2018)

30. Song, H. et al. Highly-integrated, miniaturized, stretchable electronic systems based on stacked multilayer network materials. *Sci. Adv.* **8**, eabm3785 (2022)
31. Li, J. et al. Vertically stacked skin-like active-matrix display with ultrahigh aperture ratio. *Light. Sci. Appl.* **13**, 177 (2024)
32. Zhang, T. et al. A new printed electronics approach eliminating redundant fabrication process of vertical interconnect accesses: building multilayered circuits in porous materials. *Adv. Mater. Technol.* **3**, 4 (2018)

<u>Comment #2.</u> What is the necessity of the folding? A single layer with a soft substrate without folding can also realize the functions. It is important to clarify this point in the manuscript to strengthen the creative point.

Our Response:

We sincerely appreciate the reviewer's insightful comment on the necessity of the folding structure, and we agree that clarifying this aspect will strengthen the creative point of our research. As the reviewer pointed out, it is possible to design a system-level multimodal device by placing rigid components, such as IC chips and battery on an FPCB, directly onto a soft substrate, and positioning skin-interfaced sensors or microneedle alongside them. Due to the necessity of the skin attachment of sensing electrodes or transdermal drug delivery modules, entire system components can't mount on a soft substrate if it has a single layer configuration. Without folding process, these two separate components can be electrically connected using methods such as anisotropic conductive films, magnets, or conductive epoxy, and many studies using single-layer approaches have been reported[1-3]. These approaches may address some of the issues related to mechanical strain on the rigid components, but it would still have some limitations compared to a folded structure.

First, stress distribution is key challenges in a single-layer design. Placing soft substrate under rigid IC chips can reduce the direct impact of skin movements on these components, offering some mechanical cushioning. However, this approach does not entirely mitigate the stress concentration problem, especially at the interfaces between the FPCB and the flexible parts of the device (e.g., sensors or drug delivery modules). The transition region where the rigid and flexible sections meet induces strain concentrations, which can lead to cracking, delamination, or failure in those areas. This remains a concern because the entire device will still undergo uniform bending or stretching, and the soft substrate alone cannot fully distribute the stresses across the system, thereby increasing the likelihood of potential electrical disconnection or performance degradation. In contrast, the folded structure allows for better distribution of mechanical stress across different functional layers, with the folds acting as buffers to absorb strain and protect the rigid components.

Second, mechanical adaptation to skin movement is limited in a single-layer design. Due to the non-uniform stress distribution, rigid components may resist skin deformations, causing discomfort and reducing the device's mechanical reliability over time. A folded structure, however, isolates the rigid components within the folds, allowing only the flexible components to interact with the skin. This results in improved adaptability to dynamic movements and better long-term wearability.

Lastly, the spatial efficiency provided by the folding structure is another advantage. the area required for each additional modality increases linearly within the same plane. As more functions—such as different sensor modalities or drug delivery mechanisms—are added, the device grows larger, making it less compact and more challenging to wear comfortably. In contrast, the folding structure offers a significant advantage by enabling the integration of multiple components within a smaller, more compact area. By utilizing vertical space, the

folding structure allows the various sensor and drug delivery modules to be distributed across different folds, effectively minimizing the device's footprint without sacrificing functionality. This design ensures that all necessary functions can be implemented efficiently while maintaining a small form factor.

In summary, while single-layer approaches are feasible, the folded structure offers superior stress management, mechanical adaptability, electrical reliability, and spatial efficiency, all of which are crucial for the wearable performance and multifunctionality of the device. Thus, based on the reviewer's comment, we have revised the manuscript to clarify these points and emphasize the creative significance of the folding structure.

Related references

1. Ciui, B. et al. Wearable wireless tyrosinase bandage and microneedle sensors: toward melanoma screening. *Adv. Healthc. Mater*. **7**, 1701264 (2018)
2. Kim, J. et al. Simultaneous monitoring of sweat and interstitial fluid using a single wearable biosensor platform. *Adv. Sci.* **5**, 1800880 (2018)
3. WANG, Y. et al. Wearable plasmonic-metasurface sensor for noninvasive and universal molecular fingerprint detection on biointerfaces. *Sci. Adv.*, **7**, eabe4553 (2021)

Our modification to the manuscript:

In Page 4, we have added the text:

Although many ultrathin and flexible devices have been developed to enable conformal contact[27,28], expanding those to a system-level entails incorporating various components such as power management, communication modules, signal processing modules, and drug loading chambers. Attempting to incorporate these bulky and rigid components alongside sensors and drug delivery units into a single-layer structure on a soft substrate can lead to significant challenges. For instance, due to skin deformation, such a configuration can create stress concentration at the interfaces between rigid and flexible components, increasing the risk of device delamination. This instability negatively impacts the quality of sensed data and the effectiveness of drug delivery by disrupting the device's intimate contact with the skin. To address these issues, a multi-layer approach is essential, as it allows for more effective integration and stress management across different layers. Multilayered structures can distribute mechanical stress more evenly, improve mechanical adaptation to dynamic skin movements, and enhance spatial efficiency rather than spreading them in a single plane. As one of the integration methods, stacking through via-hole processes have been predominantly used[29-32].

Comment #3. This work demonstrates the superiority of folded structures for integrating multifunctional components from a mechanical stability perspective using finite element analysis, but does not discuss the electrical reliability of biosensing and signal feedback under diverse motion states, more electrical characterization (sensitivity and operation stability for practical applications, etc.) should be added.

Our Response:

Thank you for this valuable comment. In response, we conducted additional electrical characterization to evaluate the biosensing reliability and signal feedback under diverse motion states. Specifically, we collected ECG signals and 3-axis accelerometer data while the wearer performed various movements, including sitting-to-standing transitions and walking. As shown in the attached figure, the data confirms successful acquisition of ECG signals and distinguishable 3D reconfigured motion tracking across different activities. This demonstrates that the device maintains stable electrical performance and reliable biosensing even under dynamic conditions. Thus, based on the reviewer's comment, we have revised the manuscript and added the experimental results in supplementary figure.

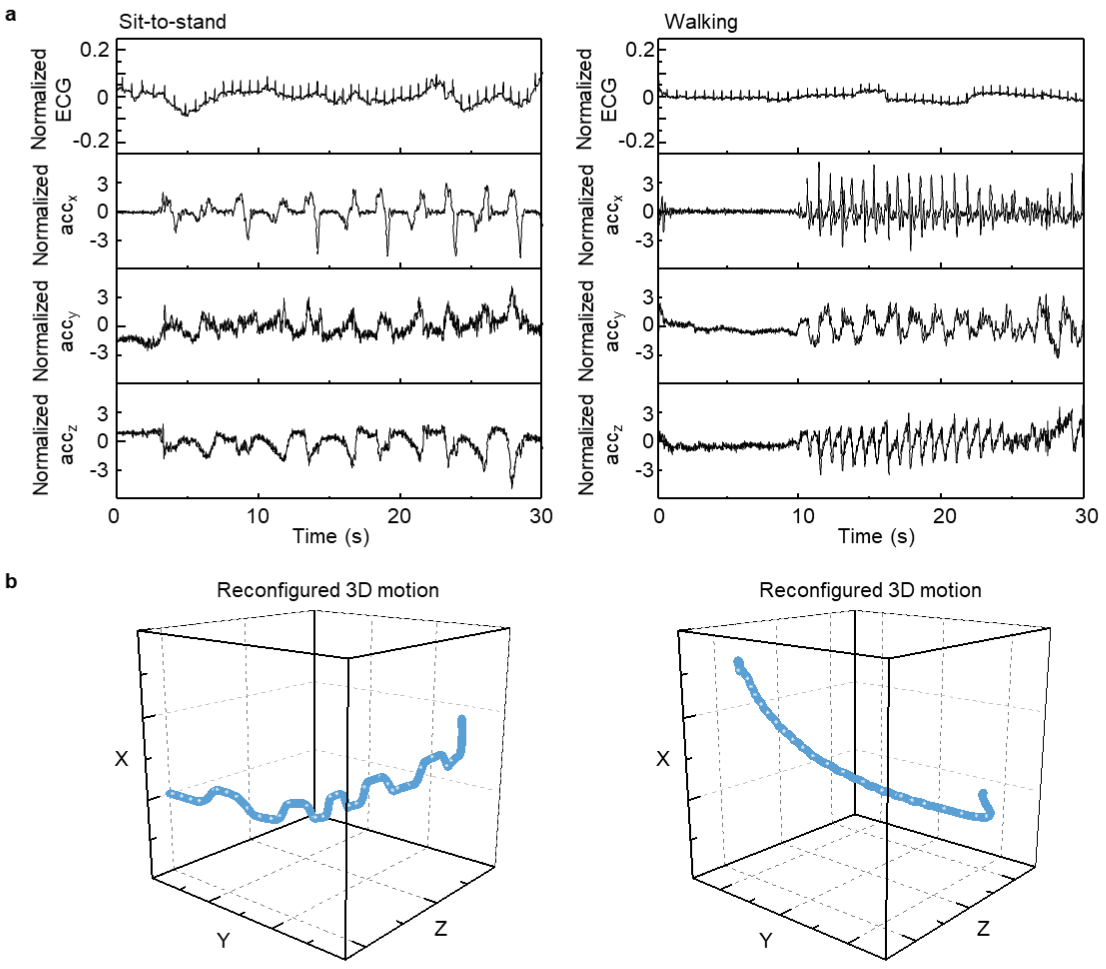

Our modification to the manuscript:

In Page 17, we have revised the manuscript as follows:

Figure 5c shows the various electrophysiological signals, including ECG, skin temperature, and body movement, through an electrical modality of the chest mounted fMMD during the desk working and walking. The folded structure and strain-isolating elastomer allow the fMMD to reliable measurements by ensuring intimate contact with the skin and reducing mechanical stress, allowing stable acquisition of signals such as ECG and 3-axis accelerometer data and tracking three-dimensional reconfigured motion even during activities like sitting-to-standing transitions, and walking (Supplementary Fig. 13).

We have added the supplementary Fig. 13:

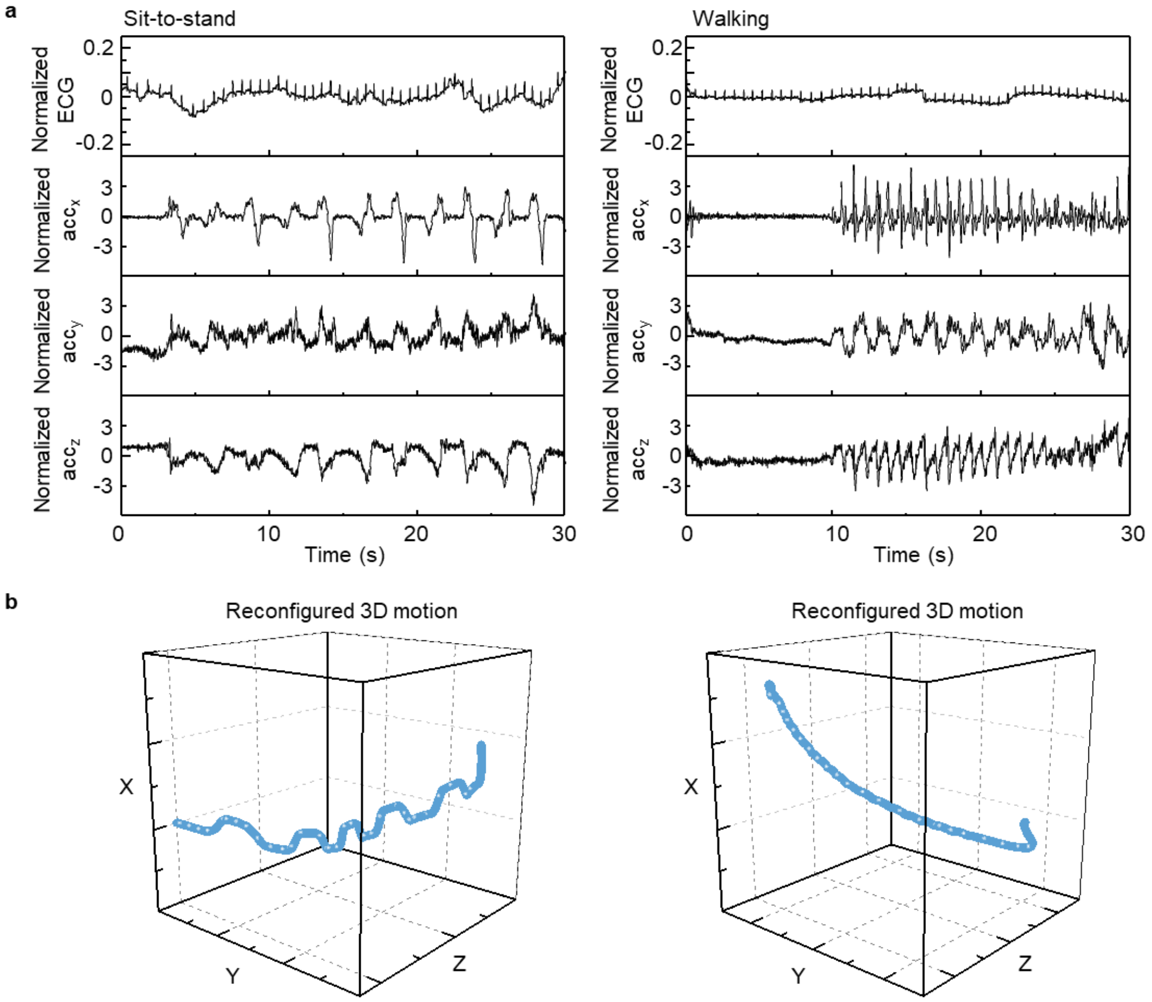

Supplementary Fig. 13 | Electrical reliability of biosensing under diverse motion states. (a) Representative recording of ECG and three-axis acceleration from fMMD attached on the wearer's chest for cases of sit-to-stand and walking. **(b)** Inferred 3D patterns of motion according to measured accelerations.

Comment #4. It is suggested to briefly discuss whether the via-hole process is applied for electrical connection, mechanical alignment, optical pathway, or fluid channel. It is confusing to merge all these functions, since in Line 86, the conductive paste is applied in the via-hole, and line 87 states the leakage of drug fluid due to misalignment.

Our Response:

We sincerely appreciate the reviewer's constructive feedback. Following your suggestion, we have expanded the discussion to further explore research involving the application of via-hole structures. Currently, via-hole structures in wearable devices are primarily focused on electrical connections[1-3]. While conventional electronic devices have established reliable methods for via-hole fabrication and filling, such methods are limited when applied to flexible and stretchable printed electronics[2]. This limitation arises from the increased process complexity, as well as the challenges posed by alignment mismatches and material damage during fabrication[4]. The conductive paste mentioned in line 86 refers to the high complexity of electrical interconnection processes, while line 87 discusses issues related to fluid leakage that can occur due to alignment mismatches and material damage.

Via holes vary structurally depending on their application, which significantly increases process complexity. For example, Through Silicon Via (TSV) is used for electrical connections[5-7], optical pathways[8, 9], and fluid channels[10, 11]. In one instance, TSV-based stacked SiP (System-in-Package) technology was utilized to fabricate a 10-layer stacked chip[6], demonstrating a liquid cooling solution incorporating both Cu TSVs and fluidic TSVs[7]. Furthermore, a 3D packaging solution integrating electrical, optical, and fluidic interconnects has been demonstrated to address challenges in power delivery, high-bandwidth communication, and cooling [8]. As these studies illustrate, vias for electrical, optical, and fluidic interconnects require different fabrication methods. Electrical vias are filled with metal, optical vias use transparent materials such as SU-8, and fluidic vias are left hollow to enable fluid circulation. This diverse approach across modalities contributes to the increased complexity of the fabrication process.

Additionally, via-hole fabrication processes face significant challenges related to alignment mismatches and material damage. Alignment issues are particularly likely when materials with different coefficients of thermal expansion (CTE) are used. Materials commonly employed in wearable devices, such as polyimide, Ecoflex, and PDMS, exhibit differing CTE values, leading to varying degrees of expansion or contraction with temperature changes, which complicates precise alignment during fabrication. These flexible materials may not remain flat during drilling, causing the laser focus to shift, potentially resulting in material damage. In laser drilling-based via-hole processes, the elevated substrate temperature may damage heat-sensitive materials, while etching-based via-hole processes may cause material damage during exposure to solvent-based developers or plasma. Such misalignment and material damage can lead to performance degradation or even fluid leakage in drug delivery systems.

Considering these factors, folding may be a more suitable option than via-hole processes for the production of multimodal devices. The folding process is less complex, allowing for easier

integration of multiple modalities while reducing the risks of alignment errors and material damage. Based on these discussions, we have revised the text to clarify concerns about the via-hole process and avoid confusion regarding its various applications in electrical connections, optical pathways, and fluid channels.

Related references

1. Joo, H. et al. Soft implantable drug delivery device integrated wirelessly with wearable devices to treat fatal seizures. *Sci. Adv.* **7**, eabd4639 (2021).
2. Jansson, E. et al. Development of a full roll-to-roll manufacturing process of through-substrate vias with stretchable substrates enabling double-sided wearable electronics. *Int. J. Adv. Manuf. Technol.* **111**, 3017–3027 (2020).
3. Poliks, M. et al. A wearable flexible hybrid electronics ECG monitor. In *2016 IEEE 66th Electronic Components and Technology Conference (ECTC)* 1623–1631 (IEEE, 2016).
4. Ramesham, R. et al. Challenges in interconnection and packaging of microelectromechanical systems (MEMS). In *2000 Proceedings. 50th Electronic Components and Technology Conference (Cat. No. 00CH37070)* 666–675 (IEEE, 2000).
5. Andry, P. et al. A CMOS-compatible process for fabricating electrical through-vias in silicon. In *56th Electronic Components and Technology Conference* (ECTC) (IEEE, 2006).
6. Jang, D. M. et al. Development and evaluation of 3-D SiP with vertically interconnected through silicon vias (TSV). In *57th Electronic Components and Technology Conference* (ECTC) (IEEE, 2007).
7. Khan, N. et al. 3-D packaging with through-silicon via (TSV) for electrical and fluidic interconnections. *IEEE Trans. Compon. Packag. Manuf. Technol.* **3**, 221–228 (2013).
8. Parekh, M. S. et al. High-performance interconnects for 3D ICs using through-silicon vias. In *2011 IEEE 61st Electronic Components and Technology Conference* (ECTC) 1992–1998 (IEEE, 2011).
9. Karppinen, M. et al. Fabrication and characterization of electrical through-vias in flexible silicon substrates. In *56th Electronic Components and Technology Conference* (ECTC) 7 pp. (IEEE, 2006).
10. Zheng, L. et al. Novel electrical and fluidic microbumps for silicon interposer and 3-D ICs. *IEEE Trans. Compon. Packag. Manuf. Technol.* **4**, 777–785 (2014).
11. Kartalov, E. P. et al. Microfluidic vias enable nested bioarrays and autoregulatory devices in Newtonian fluids. *Proc. Natl Acad. Sci. USA* **103**, 12280–12284 (2006).

Our modification to the manuscript:

In Page 4, we have updated the text:

As one of the integration methods, stacking through via-hole processes have been predominantly used[29-32]. Reliable methods for via-hole fabrication and filling have been well-established for conventional electronics; however, these approaches present limitations when applied to flexible and stretchable printed electronics[33]. Via-hole structures vary in design based on their application (e.g., electrical connections, optical pathways, or fluidic channels), [34-36] requiring precise alignment and integration with different materials, such as conductive pastes or SU-8, which increases process complexity. Furthermore, flexible materials commonly used in wearable devices, such as polyimide, Ecoflex, and PDMS, exhibit different coefficients of thermal expansion (CTE), increasing the risk of alignment mismatches[37]. During the drilling process, these materials are also prone to damage, raising concerns about fluid leakage and device integrity.

In Page 29, we have added the references:

33. Jansson, E. et al. Development of a full roll-to-roll manufacturing process of through-substrate vias with stretchable substrates enabling double-sided wearable electronics. Int. J. Adv. Manuf. Technol. 111, 3017–3027 (2020).
34. Khan, N. et al. 3-D packaging with through-silicon via (TSV) for electrical and fluidic interconnections. *IEEE Trans. Compon. Packag. Manuf. Technol.* **3**, 221–228 (2013).
35. Parekh, M. S. et al. High-performance interconnects for 3D ICs using through-silicon vias. In *2011 IEEE 61st Electronic Components and Technology Conference* (ECTC) 1992–1998 (IEEE, 2011).
36. Zheng, L. et al. Novel electrical and fluidic microbumps for silicon interposer and 3-D ICs. *IEEE Trans. Compon. Packag. Manuf. Technol.* **4**, 777–785 (2014).
37. Ramesham, R. et al. Challenges in interconnection and packaging of microelectromechanical systems (MEMS). In *2000 Proceedings. 50th Electronic Components and Technology Conference (Cat. No. 00CH37070)* 666–675 (IEEE, 2000).

Comment #5. In Figure 1a, the electrical modules cannot match the 5 modules in the main content description. For example, we cannot spot the battery and BLE module in the figure. Similar problem is observed in Figure 1b, where the position LD, waveguide, and photodetector are not clear. Maybe the author can label ① ② ③ according to the description in the main content if the space not enough to label the full names in the figure.

Our Response:

We sincerely appreciate the reviewer's constructive comment to improve the clarity and understanding of the structure of the fMMD. Since the fMMD is a standalone system capable of multimodal biosensing and drug delivery feedback, it contains a wide variety of components. Additionally, as many sub-components are integrated within the major modules, we recognize the importance of providing detailed labels and explanations for each of these elements. Matching the components described in the manuscript with corresponding numbers in Fig. 1a, b will significantly enhance the reader's understanding of the device structure. Therefore, we have labeled the relevant components in Figure 1a with matching numbers and full names, allowing users to easily identify each element and comprehend the overall structure through the figure itself. In conclusion, in response to your helpful comments, we have revised the manuscript and figures accordingly to improve clarity and ensure the correct labeling of all components.

Our modification to the manuscript:

In Page 6-7, we have updated the text:

Figure 1b represents the three modalities for closed-loop system based on simultaneous biosensing and drug delivery. In terms of biosensing, ECG, body movement, and skin temperature are acquired through folded serpentine interconnects (Fig. 1b, right). In addition to electrical signals, PDMS-based flexible optical waveguides are used to measure PPG signal. The rigid and thick components, such as (1) laser diode (LD, 650nm wavelength) and (2) photodetector (PD), are mounted on chip-integrated side, and (3) the two flexible waveguides are well aligned with the light source spot of LD and the light receiving spot of PD using optical epoxy, respectively (Fig. 1b, center). The folded waveguides aligned with the LD and PD serve as guiding the light to the skin and the reflected light from the skin. In terms of controlled transdermal drug delivery, drug delivery system is composed of three functional components (Fig. 1b, left): (1) injection trigger and pumping system with the performance of steady flow rate, (2) drug storage and microfluidic channel for delivery from reservoir to skin, and (3) microneedle array for penetration into the skin.

And, we have modified Figure 1a as follows:

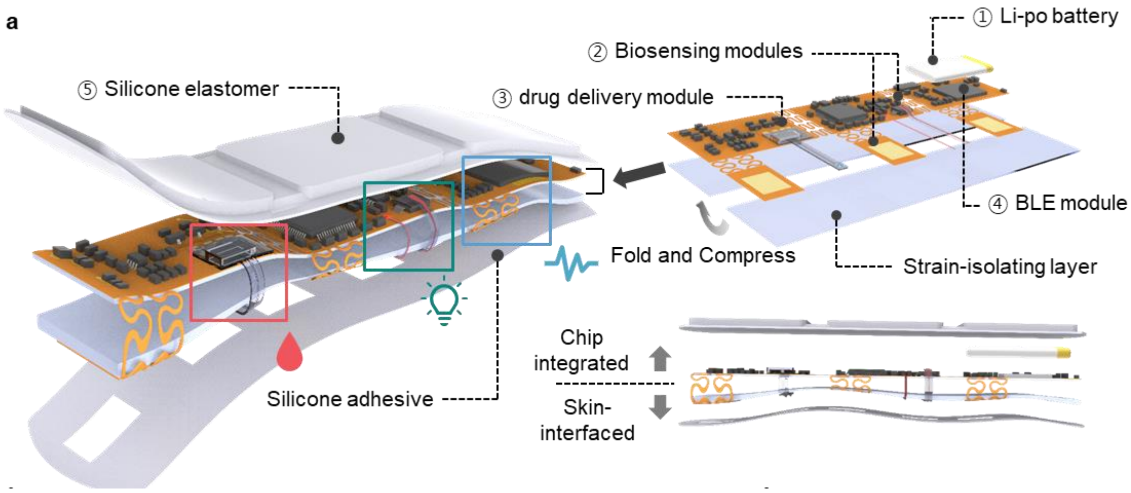

We have modified Figure 1b as follows:

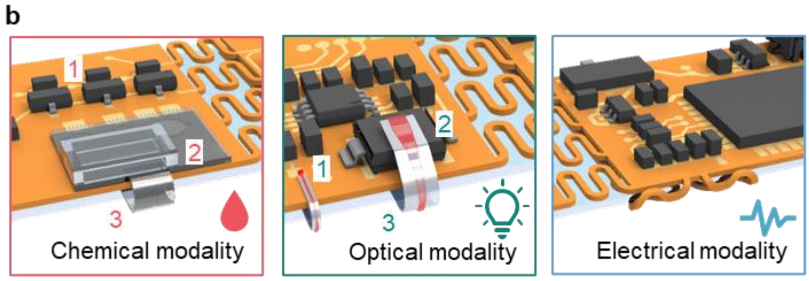

<u>Comment #6.</u> In Figure S2a, the unit seems to be wrong. Curvature radius equals to the reciprocal of curvature, where the unit for curvature radius is mm, and the unit for curvature is mm-1.

Our Response:

We would like to thank the reviewer for this observation regarding to the unit in Supplementary Fig. 2a. The relevant expression has been corrected to mm in Supplementary Fig. 2a.

Our modification to the manuscript:

In Supplementary Fig. 2a, we modify the figure and caption in terms of the expression of unit as follows:

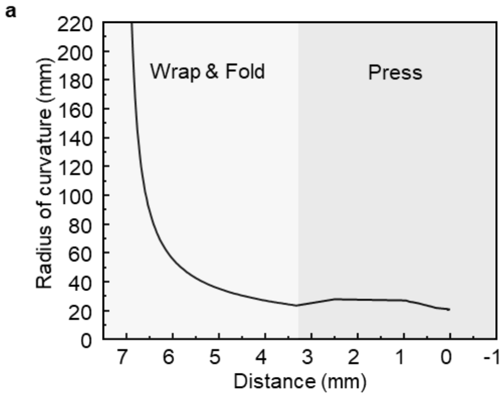

Supplementary Fig. 2 | Mechanical analysis for design optimization of the folded MMD. (a) Graph of radius of curvature versus distance during the folding process of the substrate for the straight line interconnect design. The x-axis represents the distance (mm) between the skin contact side and the non-contact side, while the y-axis indicates the radius of curvature (mm) of the straight interconnect.

<u>Comment #7.</u> The word "polymer gel" is not correct to describe the ecoFlex in the strain-isolating layer. Polymer gel usually indicates a semisolid system in a liquid medium (such as swollen by a solvent).

Our Response:

Thank you for your constructive comment. As the reviewer mentions, we recognize that "polymer gel' is not appropriate, as it typically refers to a semisolid system in a liquid medium. To represent the properties of Ecoflex, the term "polymer gel" has been replaced with "silicone elastomer" in the manuscript.

Our modification to the manuscript:

In Page 6, we modify the term as follows:

A silicone elastomer (500 μm thick) with low Young's modulus (50 kPa) provides strain-isolating effect between skin-contact side and non-contact side to mitigate the stresses at the non-contact side islands for uniaxial stretching of up to 20 %[29].

In Page 9, we modify the term of Ecoflex as follows:

To verify the overall deformability provided by the serpentine interconnect design and the strain isolation effect of the silicone elastomer, FEA was performed.

Also, in Page 10, we modify the term of Ecoflex as follows:

These results confirm that the fMMD's serpentine interconnect design and silicone elastomer layer effectively enhance deformability and provide strain isolation, ensuring stable performance during use.

<u>Comment #8.</u> The heterogeneous integration is important, in which the bonding between soft and rigid surfaces should be better studied. The FEA study in Figure 2f shows the function of the strain isolating layer, but I feel it is not enough. Could the author add a FEA study to show the strain in the heterogeneous device when attached to the skin? e.g. including rigid layer, strain isolating layer, skin, and probably the encapsulation layer. Displacement might be assigned from the skin side, and study the stress and strain on each layer of the device.

Our Response:

We sincerely appreciate the reviewer's insightful feedback. The FEA results presented in Figure 2f analyze the strain and stress on the device when attached to the skin and subjected to deformation, without the inclusion of an encapsulation layer. To focus on the specific area of interest, we post-processed the skin geometry to exclude it from the figure. During analysis, the device was attached to the skin, and then post-processing was applied to display the device as separated from the skin layer in the final visualization.

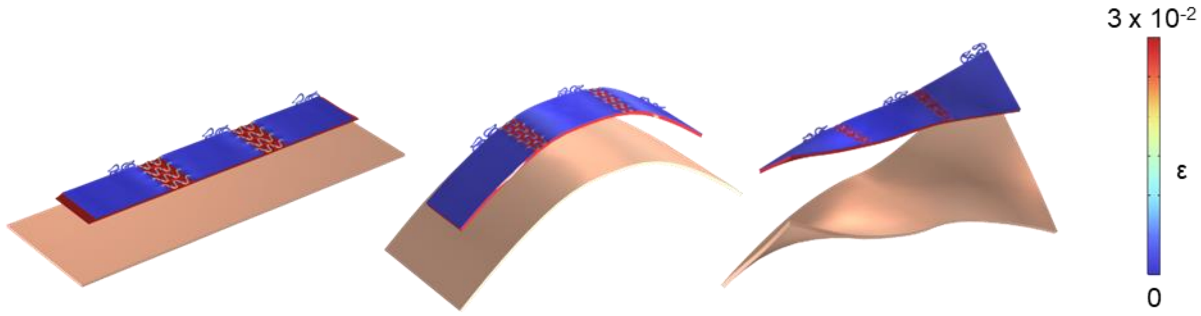

Following the reviewer's suggestion, we conducted additional analysis by adding an encapsulation layer as it would be applied to the actual device, attaching it to the skin, and assessing the strain and stress under these conditions.

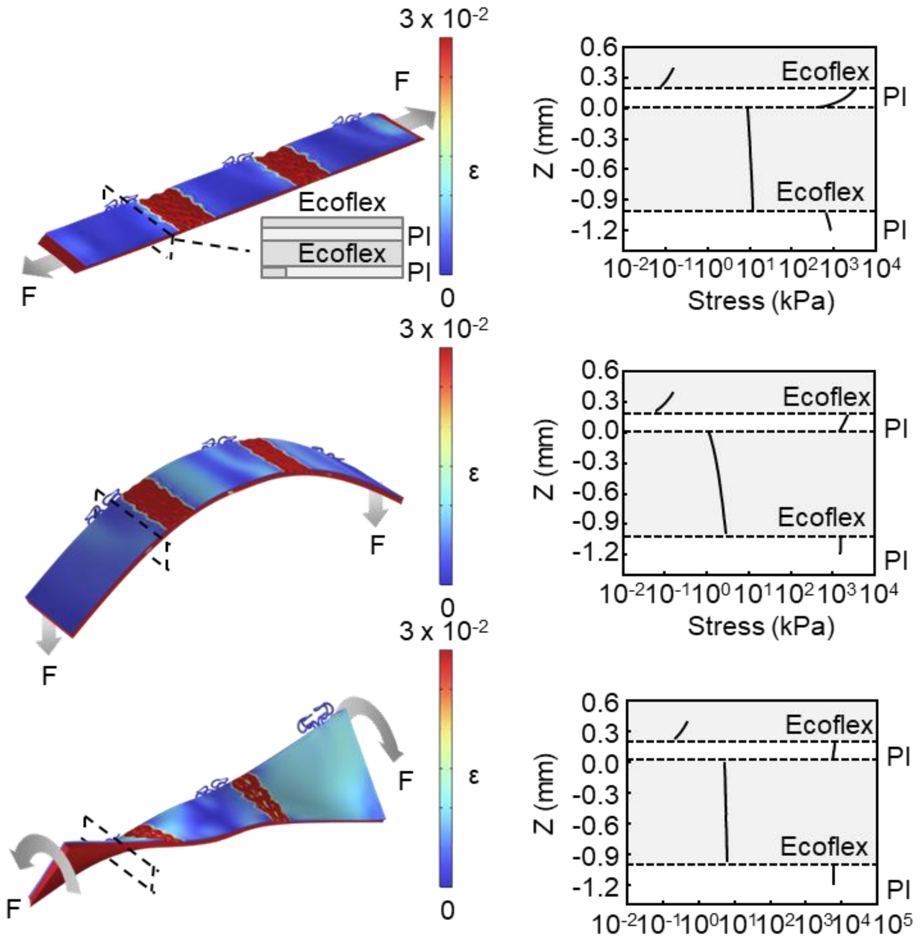

Additionally, we included the FEA analysis without the encapsulation layer in Figure 2 to illustrate that the area where the rigid component is attached experiences no strain when deformation occurs, confirming that there is no performance degradation. The analysis results with the encapsulation layer have been provided as Supplementary Fig. 3. Also, the text has been refined to clarify the analysis method for deformation applied to the skin.

Our modification to the manuscript:

We have added the Supplementary Fig. 3:

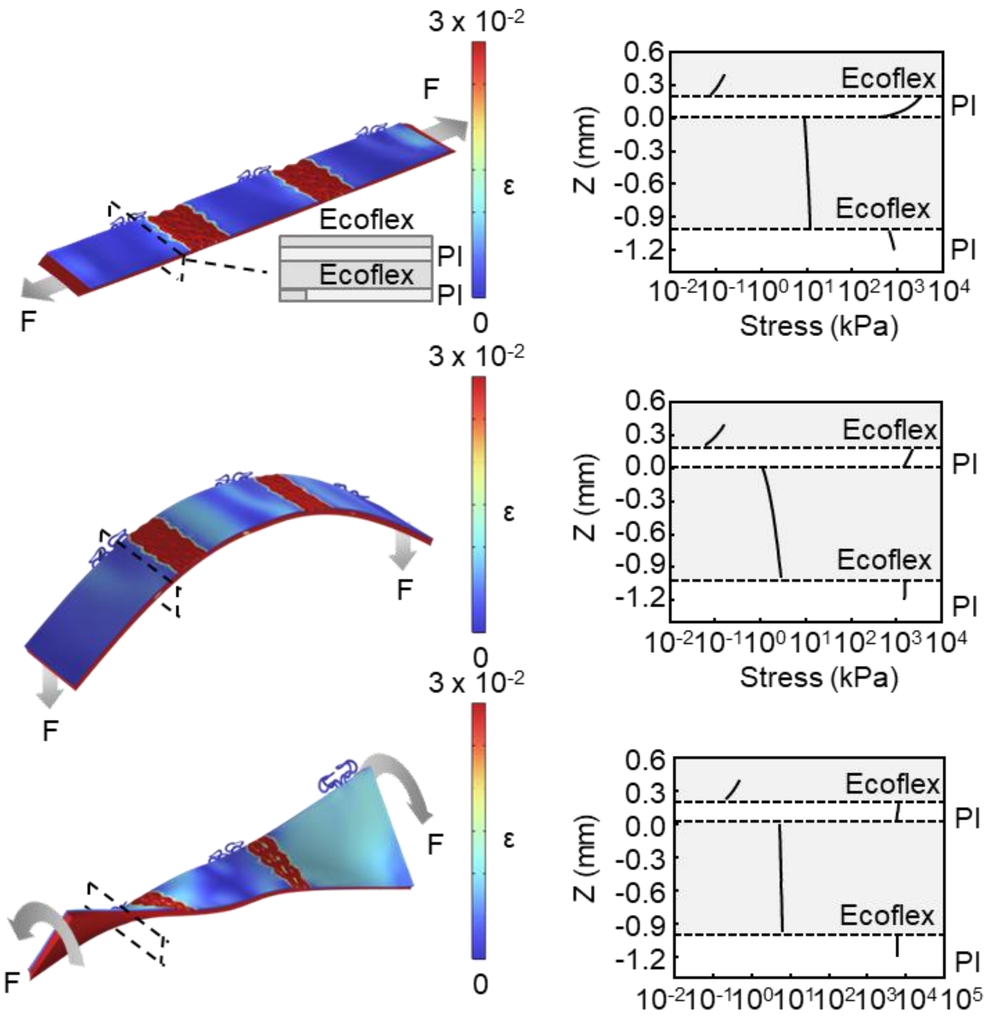

Supplementary Fig. 3 | Mechanical Properties of the fMMD with encapsulation layer. Strain and stress of fMMD with encapsulation layer under multi-deformation modes (stretching, bending, twisting). The strain-isolating effect is demonstrated for deformations applied to the skin-interfaced layer, as shown by stress analysis on a cross-sectional plane (indicated by the black dashed box).

In Page 9, we have updated the text about Supplementary Fig. 3 in Result section as follows:

The strain associated with multi-deformation modes is illustrated, and the stress can be observed in the graph. Similarly, strain and stress analysis for the encapsulated device was conducted under various deformation modes (stretching, bending, and twisting), confirming that the strain in the PI upper layer, where the actual chip is integrated, remains within 0.03 in both the presence and absence of the encapsulation layer(Fig. 2f and Supplementary Fig. 3).

In Page 25, we have updated the text about Fig 2f. and supplementary Fig. 3 in Method section as follows:

Strain was analyzed at each stage of folding process and various deformation modes. The devices with and without an encapsulation layer were laminated onto the skin and subjected to various deformations. The strain and stress of the devices were then analyzed under these skin deformations (stretching, bending, and twisting). After the analysis, the skin topography was post-processed to ensure it was not visible.

Comment #9. Since the whole device is encapsulated with a white color polymer, how can we determine when the drug has been used up? And how to top up the drug?

Our Response:

We sincerely appreciate the reviewer's thoughtful question regarding drug depletion and refilling in the encapsulated fMMD. We agree that determining the drug's usage status and simplifying the refilling process are important considerations. As the reviewer noted, the system is entirely encapsulated in a white-colored polymer, which prevents direct visual confirmation of drug depletion. Alternatively, the tracking the duration of the pumping actuation can be used as an indirect method for monitoring the drug status. The point at which the drug is nearly depleted can be inferred by correlating the pumping frequency, the flow rate of each drug delivery operation, and the total capacity of the drug storage chamber. This method provides an estimation based on the operational parameters of the micropump.

Regarding the drug loading process, the reservoir is manufactured by pouring PDMS into a mold and curing it, with a total capacity of 21 μL. To ensure that the pressure from the micropump is fully transferred to the microfluidic channel without pressure leakage, the reservoir is designed to have a completely sealed structure. Because of this, the drug is filled by inserting a 27-gauge syringe into the top of the PDMS reservoir. Due to the elasticity and self-healing properties of PDMS—with a thickness of 3 mm or more—the reservoir reseals itself completely when the syringe is removed after refilling.

However, these indirect monitoring methods and reservoir designs still have limitations in terms of accurate and convenient drug management. To enhance user interaction and improve drug monitoring, the inferred amount of drug used could also be displayed graphically on a user interface (UI). This approach would allow users to monitor the drug usage visually, offering an intuitive way to track the remaining drug amount. In addition, by attaching a septum-structured refill port to the area where the reservoir is located in the encapsulated device[1], convenient reloading can be achieved through an externally exposed refill port structure, while maintaining the device's sealed integrity.

Related reference

1. Forouzandeh, F. et al. A Wirelessly Controlled Scalable 3D-Printed Microsystem for Drug Delivery. *Pharmaceuticals.* **14**, 6 (2021)

<u>Comment #10.</u> The authors utilize a folded structure with serpentine interconnections to guarantee seamless integration of multiple sensing modalities for real multi-pathway biosensing and on-demand drug delivery. However, a great deal of real-time monitoring of biosignals and qualitative analysis is absent in practical applications. Possible solutions to achieve a better design of the sensing-delivery-feedback platform to tackle the point should be provided and discussed.

Our Response:

Thank you for your insightful comment. We agree that real-time monitoring and qualitative analysis of biosignals are crucial for improving the functionality of the biosensing-delivery-feedback platform. Currently, our system is focused on biosensing, which can analyze a combination of electrophysiological and optical-based pulse wave signals. Further approaches to advance the current fMMD can be summarized in three approaches: 1) Advanced electrophysiological analysis, 2) Integration of electrochemical sensing modality, and 3) Development of effective micropump for acute care.

Advanced electrophysiological analysis, including metrics like heart rate variability (HRV)[1], blood pressure[2], and arrhythmia detection through Extended Continuous Rhythm Monitors (ECRM)[3] such as Holter monitors, plays a crucial role in monitoring and diagnosing a variety of cardiovascular and autonomic nervous system disorders. HRV analysis, which evaluates the fluctuations in the time interval between heartbeats, provides valuable insights into the balance between the sympathetic and parasympathetic nervous systems. Low HRV is associated with conditions like cardiovascular disease, diabetes, and stress-related disorders, indicating impaired autonomic regulation. Blood pressure monitoring, particularly through continuous wearable devices, is beneficial for managing hypertension and detecting hypertensive episodes, which can prevent complications such as stroke, heart attack, and kidney damage. ECRM can enhance arrhythmia detection by recording heart rhythm over longer durations than traditional ECGs, which is especially useful for capturing infrequent or asymptomatic arrhythmic events like atrial fibrillation, premature ventricular contractions, and other irregular heart rhythms. Early detection of these arrhythmias can prevent severe complications such as stroke, heart failure, and sudden cardiac death. Advanced continuous rhythm monitoring is essential for personalized and preventive cardiac care, offering critical information that aids early intervention and supports effective chronic disease management.

In response, we have conducted two in-depth analysis to enhance the qualitative biosensing capabilities of the device, particularly for cardiovascular monitoring and autonomic nervous system monitoring. First, for real-time blood pressure-related monitoring, we analyzed pulse arrival time (PAT) before and after exercise. During desk work, the PAT is relatively high ranging from 280 ~ 320 ms. In contrast, following exercise, the PAT shows noticeable changes, with a shorter interval between the ECG and PPG peaks. This reduction in PAT reflects an increase in blood pressure, which is typical after physical exertion due to elevated heart rate and vascular responses. The significant difference in PAT between desk work and post-exercise conditions verifies the device's capability to detect real-time cardiovascular changes associated with varying physiological states. This capability suggests that fMMD can provide valuable

insights into cardiovascular health and potentially be used for continuous blood pressure monitoring in various everyday scenarios.

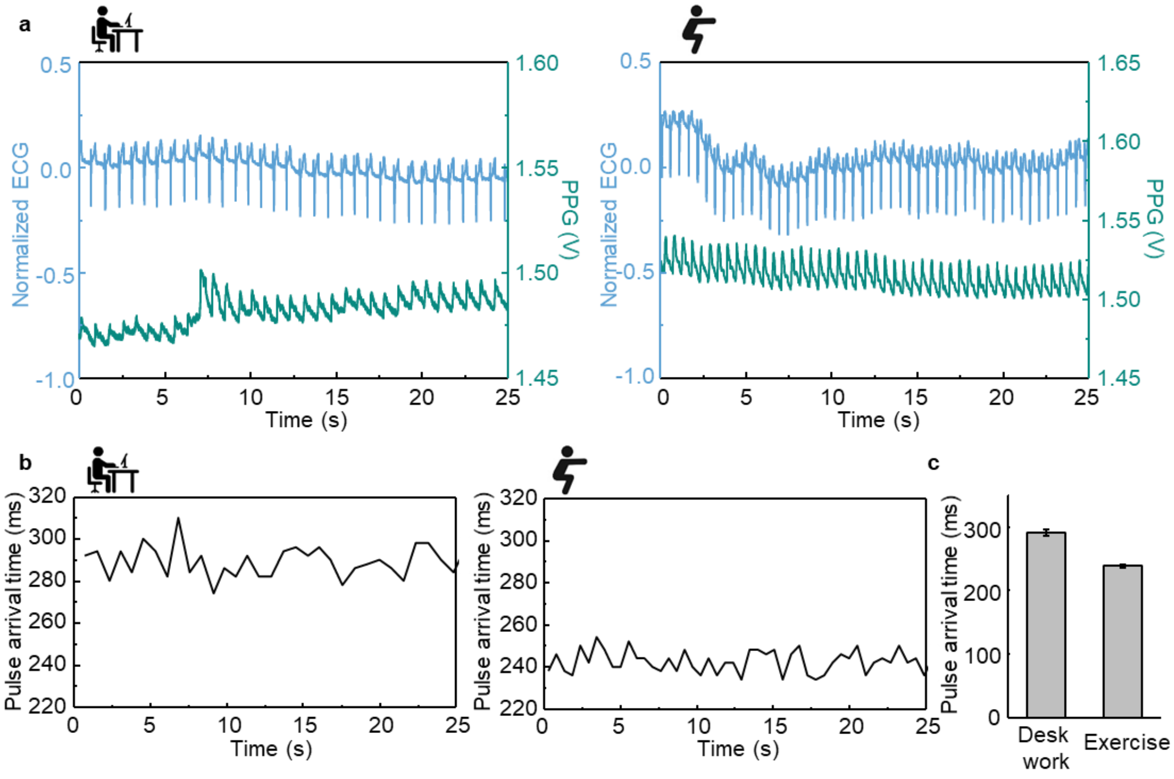

Second, we monitored heart rate variability (HRV) through ECG measurements taken immediately after exercise and continuing through the recovery period for several minutes. We demonstrated the body's recovery response after exercise, characterized by a gradual decrease in heart rate and a concurrent increase in HRV and the rate of HRV change. These trends provide valuable insights into the body’s autonomic nervous system balance and resilience, with HRV serving as a reliable indicator of relaxation and adaptive capacity. This approach allows for HRV monitoring as an indicator of autonomic nervous system function, which can provide valuable insights into stress levels and recovery states. Both measurements demonstrate the potential of this platform for continuous and qualitative cardiovascular and mental health monitoring.

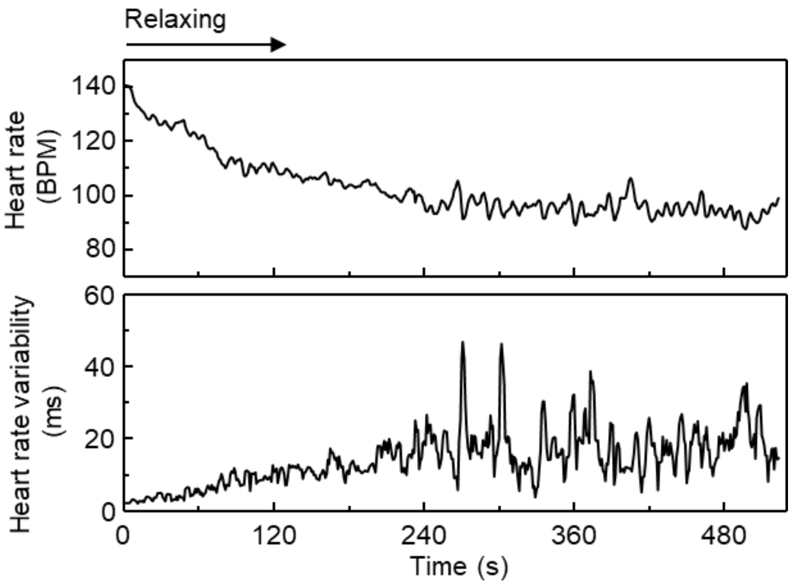

Also, skin-interfaced devices that combine electrochemical and physiological biomarkers enable comprehensive monitoring across various health domains by capturing biochemical and electrophysiological changes in real time[4]. Integrating lactate and ECG measurement, for example, allows for assessment of cardiovascular and metabolic health during physical exertion, supporting heart disease and respiratory disorder management. Hybrid systems that track glucose alongside heart rate, oxygen saturation, and activity levels offer targeted diabetes management, especially to prevent hypoglycemia post-exercise. For exercise physiology, tracking lactate, pH, and ECG enables real-time monitoring of metabolic acidosis and cardiac function, aiding in both performance assessment and recovery. Comprehensive wearable devices also monitor lifestyle and metabolic health, combining parameters like glucose, lactate, caffeine, alcohol, blood pressure, and heart rate during daily activities to provide actionable insights for managing diabetes, hypertension, and related conditions. Additionally, sensors for muscle contraction and strain can aid rehabilitation and prosthetic control by accurately tracking muscle activity and strain, demonstrating the utility of multiplexed biomarker analysis in both chronic disease and acute care contexts. As this technology advances, improvements in integration and precision will further enhance its applicability to personalized healthcare and disease management.

In terms of delivery platform, the development of a micropump suitable for acute care applications is an essential step in advancing wearable drug delivery technology. Currently, thermo-pneumatic actuation micropumps, which utilize controlled heating to generate pressure and facilitate drug release, are effective for continuous, low-flow-rate delivery suitable for chronic care. This type of steady, gradual administration is ideal for managing conditions that require long-term maintenance, such as diabetes (for insulin), hypertension (with medications like beta-blockers or calcium channel blockers)[5], and chronic pain (using analgesics)[6]. Such diseases benefit from stable, controlled drug release that maintains therapeutic levels over extended periods without necessitating immediate response. However, in acute care, rapid and

responsive drug delivery is critical to effectively manage sudden medical emergencies like cardiac arrest, asthma attacks, or severe pain, where immediate administration of medications such as epinephrine, bronchodilators, or opioids is essential[7]. Unlike chronic applications, acute care requires the micropump to deliver a higher volume of medication in a shorter timeframe to promptly stabilize the patient. Therefore, transitioning from chronic to acute applications demands several modifications to the micropump's design: enhancing the pumping rate and pressure for higher, faster dosing and ensuring it remains compact and portable to support wearable use. Developing an advanced pumping mechanism with faster response times and precise control can meet these high-delivery demands, enabling wearable devices to handle both chronic and acute care seamlessly, thereby broadening their clinical applications and utility in diverse healthcare settings.

In summary, we are pleased to report the successful further verification of cardiovascular and stress-related biomarker monitoring using this platform. By analyzing biomarkers such as pulse arrival time (PAT) for real-time blood pressure monitoring and heart rate variability (HRV) for stress assessment, we demonstrate the platform's capability for comprehensive biosignal monitoring. In response to the reviewer's comments, we have incorporated a discussion of possible solutions for optimizing the sensing-delivery-feedback design in the revised manuscript. This discussion further clarifies how real-time biosignal monitoring and qualitative analysis can be achieved, enhancing the platform's application in personalized healthcare.

Related references

1. Rajendra A. U. et al. Heart rate variability: a review. *Medical and biological engineering and computing*, **44**, 1031-1051 (2006)
2. Meidert, A. S. ., and Saugel B., Techniques for non-invasive monitoring of arterial blood pressure. *Frontiers in medicine*, **4**, 231 (2018)
3. Schultz K. et al. The days of the Holter monitor are numbered: Extended continuous rhythm monitoring detects more clinically significant arrhythmias in adults with congenital heart disease. *Circulation*, **134**, A13416-A13416 (2016).
4. Jung, H.H., et al. Wearable electrochemical sensors for real-time monitoring in diabetes mellitus and associated complications. **4**, 15, (2024)
5. Opie L. H. et al, Current Status of Safety and Efficacy of Calcium Channel Blockers in Cardiovascular Diseases: A Critical Analysis Based on 100 Studies. *Progress in cardiovascular diseases*, **43**, 171-96 (2000)
6. Lynch M. E. et al, The Pharmacotherapy of Chronic Pain: A Review. *Pain Resear ch and Management*, **11**, 11-38 (2006)
7. Zhu F. et al, Integrated Multifunctional Flexible Electronics Based on Tough Supra-molecular Hydrogels with Patterned Silver Nanowires. *Journal of Materials Chemistry C*, **8**, 7688-97 (2020)

Our modification to the manuscript:

We have added the supplementary Fig. 14, 15:

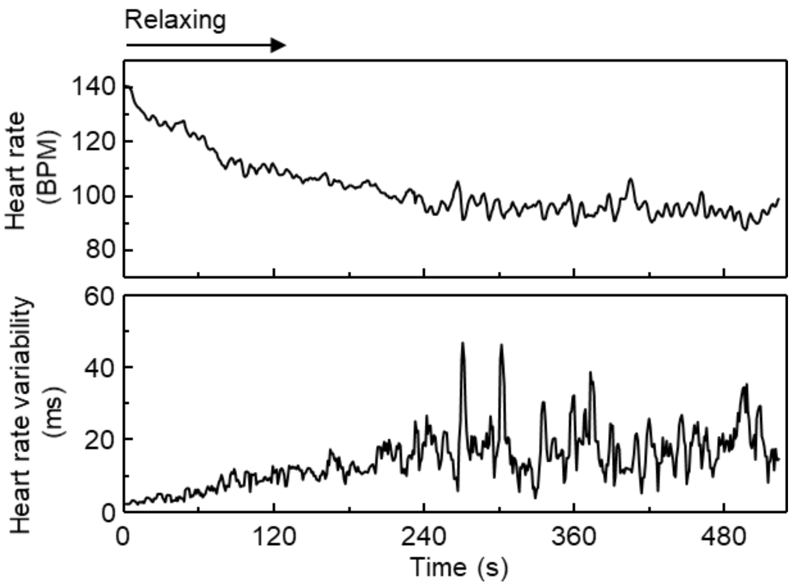

Supplementary Fig. 14 | Real-time measurement of heart rate and resulting heart rate variability during post-workout recovery.

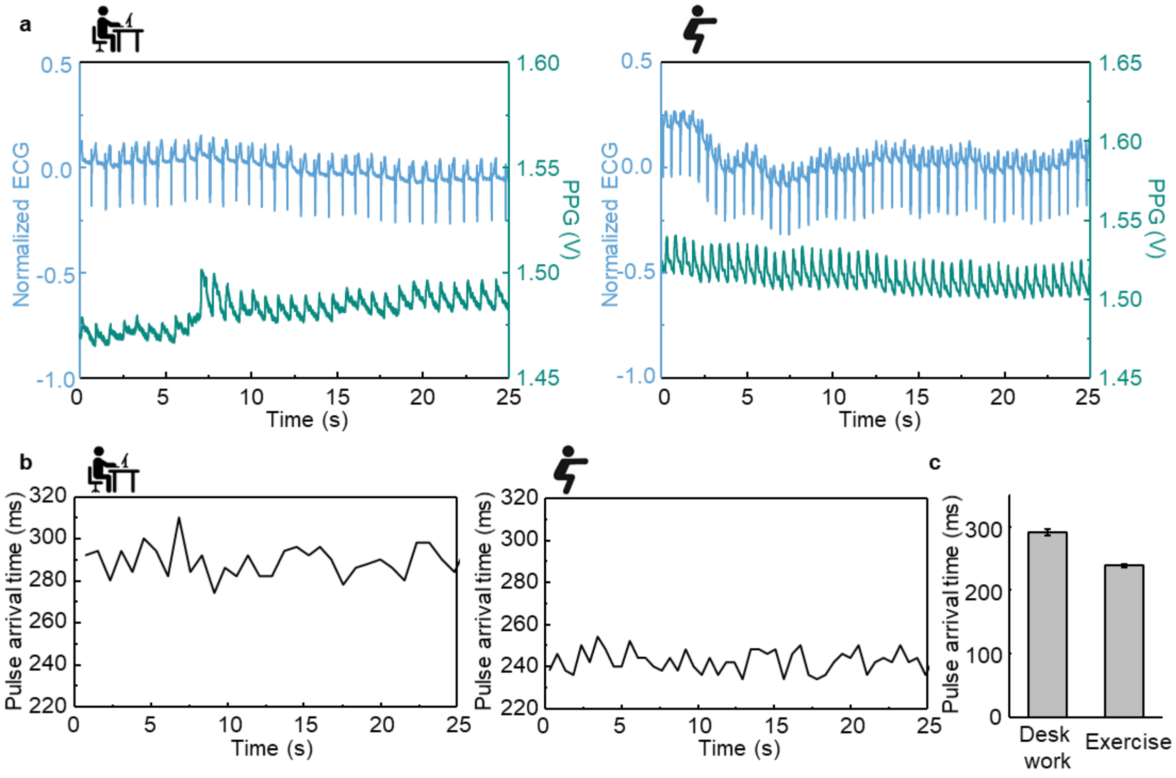

Supplementary Fig. 15 | Comparison of changes in pulse arrival time over time before

and after exercise. (a) Real-time measurement with optical and electrical modalities during desk work and after squat workout. **(b)** Calculation of PAT over time for two activities (desk work and exercise). **(c)** Comparison of average PAT for measuring time (3 trials).

In Page 17, we have updated the text about Supplementary Fig. 14 in Result section as follows:

Figure 5c shows the various electrophysiological signals, including ECG, skin temperature, and body movement, through an electrical modality of the chest mounted fMMD during the desk working and walking. The folded structure and strain-isolating elastomer allow the fMMD to reliable measurements by ensuring intimate contact with the skin and reducing mechanical stress, allowing stable acquisition of signals such as ECG and 3-axis accelerometer data and tracking three-dimensional reconfigured motion even during activities like sitting-to-standing transitions, and walking (Supplementary Fig. 13). Supplementary Fig. 14 demonstrates the capability of Case 1 measurement to perform heart rate and heart rate variability (HRV) analysis during post-workout recovery. The gradual decrease in heart rate and the corresponding increase in HRV and the rate of HRV changes reflect the body's shift to a relaxed state, indicating effective monitoring of autonomic function. This setup can be applied to cardiovascular health assessment and stress recovery monitoring in daily life.

In Page 18, we have updated the text about Supplementary Fig. 15 in Result section as follows:

The synchronized ECG and PPG from the fMMDs on the chest and wrist enable the monitoring of pulse arrival time (PAT) as shown in Fig. 5d (Case 2, single modal biosensing at multi-spots), which can be used as a medical indicator for sleep quality, arterial stiffness, and myocardial ischemia[46-48]. Calculation of the time interval between peaks of ECG and PPG enable to acquire the PAT as shown in Fig. 5e, f. Supplementary Fig. 15a, b show the capability of Case 2 measurement to monitor PAT changes before and after exercise, using synchronized ECG and PPG signals from the fMMDs on the chest and wrist. The average of PAT decreases following exercise (239 ms ± 2.9 ms) compared to desk work (291 ms ± 4.7 ms), which aligns with increased cardiovascular activity and elevated blood pressure after physical exertion. This real-time monitoring of PAT highlights the device's potential for blood pressure-related assessments and applications.

In the Discussion section, Page 20, we have revised the text as follows:

Additionally, the proposed fMMD has proven its capability to derive a multiplexed factor, such as real-time PAT, and HRV in diverse physiological states and trigger the self-directed drug delivery based on the collected signal as a closed-loop system.

In Method section, Page 27, we have updated the signal processing and calculation of heart rate variability.

Data analysis and algorithms for multiplexed measurement and closed loop operation

For real-time measurement, the fMMDs were attached on human subject's chest and wrist, respectively. Chest unit measured the ECG, skin temperature, and acceleration at a sampling rate of 500 Hz and wrist unit operated the PWM to trigger LD and measured PPG signal through a series of analog signal processing including analog filters, inverting amplifiers. The collected signals were digitized with 32-bit ADC and transmitted to the personal laptop. Signal processing used a detrend algorithm to remove baseline wander using analysis software (MATLAB R2023b). Peak detection algorithm acquired peak-to-peak time interval for ECG and PPG signals. PAT was calculated from the time intervals between simultaneously obtained ECG and PPG signal waveforms. In addition, the collected ECG signal from chest unit was used to calculate heart rate in real time, utilizing a window size of 5 s. For HRV calculation, a sliding window with a size of 5 s and an overlap of 1 s was applied to the ECG signal, using the intervals between peaks to compute HRV. The Root Mean Square of Successive Differences (RMSSD) was employed as the HRV metric, as it is particularly sensitive to high-frequency variations and provides an effective measure of parasympathetic nervous system activity, making it well-suited for assessing short-term changes in autonomic function. The data packet to initiate drug delivery was transmitted wirelessly from the MATLAB to the fMMD when the calculated heart rate exceeds 120 bpm as a way to show a closed-loop system.

Reviewer# 3

In this manuscript, the authors describe a multilayered, flexible, skin-mounted device made by folding an electronic circuit board. In addition to electrical circuits, they show that folding can be applied to chemical and optical modalities, and demonstrate a functional device by acquiring PPG, ECG, and temperature on a single subject. While the idea of folding multilayered circuit boards is simple and has been used in prior work, the extension to other modalities in a functional device is interesting and useful. I found the manuscript to be written and presented clearly. My specific comments are below:

Our response:

We sincerely appreciate the reviewer's helpful and detailed feedback on our manuscript. We are pleased that the extension of the folding process to integrate multiple modalities in a functional, skin-mounted device was found to be both interesting and useful. The reviewer's specific comments, particularly regarding the use of via-hole technology versus the folding process, have been extremely valuable in improving the logical flow and coherence of the manuscript. These insights helped us clarify our approach and present it more effectively. Additionally, the reviewer's suggestions to improve certain expressions and explanations have greatly contributed to making the manuscript more understandable for readers. We have carefully addressed each specific comment and revised the manuscript accordingly, ensuring that these improvements reflect the reviewer's valuable input. Thank you once again for your thorough review, which has played a significant role in enhancing the quality and clarity of our work.

Comment #1. Introduction: "However, this method requires complex procedures which are individually fabricating each single layer with via-holes, precisely aligning them, and connecting with conductive tape or paste."

a. Vias are a well developed technology in the PCB industry and can be reliably mass produced even on very thin PCBs. While I understand that standard industry processes cannot be used to produce stacked circuit layers that retain the flexibility of a single layer, it seems that they could be used to produce the double-sided circuit boards considered here. Could the authors clarify this statement, or focus more on the need for multimodal connectivity between layers rather than on the disadvantages of vias?

b. Following the above, the relative advantages and disadvantages of folding and vias should be discussed in a more nuanced manner in the Discussion section. I do not see folding as the superior choice to vias in many situations, including when many inter-layer connections are needed, the total thickness needs to be small, and there are limited placement options near the edges.

Our Response:

We appreciate the reviewer's constructive comments. In response to your suggestion, we have expanded the discussion by focusing on the necessity of folding. As mentioned in the first suggestion, double-sided and multi-layered structures can be fabricated using both via and folding methods [1]. Via structures are typically fabricated through sequential layer formation using manual drilling or etching. In contrast, folding structures are created through the folding process after the fabrication is complete. As mentioned in the Introduction, certain considerations must be taken into account when applying these structures to wearable devices with multimodal functionality. Specifically, rigid components must be integrated at the system level for multimodal functionality while maintaining flexibility to ensure comfortable adherence to the skin. Via structures, by enabling vertical connections, can be used to create thin double-sided structures, which can improve comfort when applied to wearable devices. However, if power management, communication, or signal processing modules are attached to one side of a thin, double-sided structure, it may lead to delamination from the skin. On the other hand, while folding-based structures may result in a thicker design, the inclusion of a strain-isolating layer can prevent delamination by placing rigid components on the side opposite to the skin. Therefore, this paper aims to develop a system-level multimodal device using a via-less folding structure.

Additionally, as suggested in the second suggestion, we have conducted a more thorough comparison of the advantages and disadvantages between folding and via-hole structures. Via-hole processes enable vertical stacking of chips, which reduces interconnection lengths and thus significantly improves both signal delay and power loss, contributing to enhanced system integration and miniaturization [2,3]. This process is particularly effective for devices using metal oxide semiconductors or chemically stable 2D semiconductors, as these materials are well-suited to lithography and etching-based fabrication techniques[4-6]. However, challenges arise when organic materials such as polyimide, Ecoflex, and PDMS are involved. These materials may suffer from alignment mismatches due to the differences in coefficients of

thermal expansion (CTE) between different substrates, exacerbating potential misalignments during processing. In particular, flexible materials may not maintain flat surfaces during drilling, leading to laser focus issues, while laser drilling itself can elevate substrate temperatures, risking damage to heat-sensitive materials. Etching-based via-hole processes can also introduce damage from solvent-based developers or plasma exposure, further complicating the integration of organic or flexible substrates. The complexity of via-hole fabrication varies significantly depending on the application. For instance, TSV-based interconnects for electrical, optical, and fluidic functions require different fabrication techniques for each modality, increasing the overall process complexity. Electrical vias are typically filled with conductive metals, optical vias utilize transparent materials such as SU-8, and fluidic vias remain hollow to allow the flow of fluids. [6-8] The need to employ distinct fabrication methods for each modality can add considerable complexity to the manufacturing process, impacting overall scalability and efficiency.

In contrast, the folding process offers a simplified approach that is highly advantageous for integrating multiple modalities into a single device. Since the structure is implemented by physically folding the device, there is no need for complex processes such as drilling, etching, or filling via-holes, resulting in a lower overall process complexity. This also eliminates the risk of alignment mismatches, which are common in more intricate fabrication methods. Consequently, the folding process can significantly reduce manufacturing time and costs, making it an attractive option for device fabrication. One of the main benefits of this simplified approach is the ability to easily integrate different functionalities (electrical, optical, fluidic), even when each modality is produced using different fabrication techniques. For instance, in the case of the discussed device, the drug delivery module was fabricated using a soft lithography-based molding technique, while the electrical connections were created using photolithography. Despite the differences in these manufacturing processes, the folding process allows for these components to be smoothly integrated into a cohesive system that operates efficiently. Furthermore, the folding structure enhances space efficiency by enabling the integration of multiple functions in a compact horizontal area. While several modalities could result in an increased device footprint, the folding technique reduces this impact by utilizing vertical space effectively. However, as the folding process builds upon the vertical dimension, there is a trade-off in terms of device thickness. Compared to traditional via-hole structures, the overall thickness of the device may increase slightly due to the stacked nature of the folded components. Nonetheless, the benefits of easier integration, reduced complexity, and cost-effective production make the folding process a compelling option for advanced multimodal devices.

In summary, both via-hole and folding processes have distinct advantages and disadvantages. Via-hole processes are ideal for creating vertical interconnections, significantly reducing signal delay and power loss, and facilitating miniaturization. However, they pose challenges with flexible materials and are complex, particularly when integrating multiple modalities. On the other hand, folding provides a simplified process, allowing for easier integration of various modalities and reduced complexity, but can result in slightly thicker devices. Given the specific requirements of the multimodal device proposed in this paper, the folding method is more

appropriate. Its flexibility, combined with manageable complexity, makes it the best option for achieving the desired functionality.

In accordance with the reviewer's suggestions, we have incorporated the points raised in a into the Introduction, and the feedback from b has been addressed in the Discussion section as outlined below. Through these revisions, we have aimed to deepen the analysis of the paper, providing a more comprehensive and well-rounded exploration of the subject matter.

Related references

1. Kim, S. et al. Vertically integrated electronics: new opportunities from emerging materials and devices. *Nano-Micro Lett.* **14**, 201 (2022).
2. Savidis, I. et al. Electrical modeling and characterization of through-silicon vias (TSVs) for 3-D integrated circuits. *Microelectron. J.* **41**, 9–16 (2010).
3. Jang, Y. J. et al. Advanced 3D Through-Si-Via and Solder Bumping Technology: A Review. *Materials* **16**, 7652 (2023).
4. Joo, H. J. et al. Oxide thin-film transistor-based vertically stacked complementary inverter for logic and photo-sensor operations. *Materials* **12**, 3815 (2019).
5. Zhao, C. et al. Evaporated tellurium thin films for p-type field-effect transistors and circuits. *Nat. Nanotechnol.* **15**, 53–58 (2020).
6. Ramesham, R. et al. Challenges in interconnection and packaging of microelectromechanical systems (MEMS). In *50th Electronic Components and Technology Conference*, 666–675 (IEEE, 2000).
7. Priyabadini, S. et al. 3-D stacking of ultrathin chip packages: An innovative packaging and interconnection technology. *IEEE Trans. Compon. Packag. Manuf. Technol.* **3**, 1114–1122 (2013).
8. Yoo, H. et al. Highly stacked 3D organic integrated circuits with via-hole-less multilevel metal interconnects. *Nat. Commun.* **10**, 2424 (2019).

Our modification to the manuscript:

In the Introduction section, Page 4-5, we have revised the text as follows:

As one of the integration methods, stacking through via-hole processes have been predominantly used[29-32]. Reliable methods for via-hole fabrication and filling have been well-established for conventional electronics; however, these approaches present limitations when applied to flexible and stretchable printed electronics[33]. Via-hole structures vary in design based on their application (e.g., electrical connections, optical pathways, or fluidic channels),[34-36] requiring precise alignment and integration with different materials, such as conductive pastes or SU-8, which increases process complexity. Furthermore, flexible materials commonly used in wearable devices, such as polyimide, Ecoflex, and PDMS, exhibit different coefficients

of thermal expansion (CTE), increasing the risk of alignment mismatches[37]. During the drilling process, these materials are also prone to damage, raising concerns about fluid leakage and device integrity.

As an alternative to via-hole process, a method has been developed that fabricates overall systems in a single layer and then folds them at once[38-40]. Despite these advantages of folding approach in terms of integrity and functionality, the folded structure of wearable device has so far been applied only to implementing electrical connections in a folded structure. This approach simplifies the fabrication process by removing via holes, facilitating the integration of multiple modalities. Additionally, it allows for conformal contact with the skin, even with the inclusion of rigid components, thereby enabling effective multimodal biosensing and drug delivery.

In the Discussion section, Page 19, we have revised the text as follows:

However, challenges exist from various aspects, including a system-level configuration, drug dose management, and integration into a single device without compromising flexibility or stretchability[9]. In terms of device fabrication, both via and folding methods can be considered for implementing double-sided and multilayer structures. The via method facilitates vertical stacking, which can be advantageous for system integration and miniaturization; however, the structural variation required for different applications increases process complexity and can lead to alignment mismatches and potential damage when integrated with flexible components.[37,60] In contrast, the folding method may result in a slightly thicker device but is advantageous for integrating multiple modalities due to its lower process complexity. Additionally, by incorporating a strain-isolating layer, the folding approach can prevent delamination from the skin caused by rigid components, making it more suitable for system-level multimodal devices. Accordingly, in this study, we introduce a unified platform by incorporating multiple modalities – electrical, optical, and chemical – with a folded structure, which enables multiplexed biosensing and on-demand drug delivery. We have designed functionally sectioned islands on a FPCB, connected by serpentine interconnects.

In Page 29-30, we have added the references:

33. Jansson, E. et al. Development of a full roll-to-roll manufacturing process of through-substrate vias with stretchable substrates enabling double-sided wearable electronics. Int. J. Adv. Manuf. Technol. 111, 3017–3027 (2020).
34. Khan, N. et al. 3-D packaging with through-silicon via (TSV) for electrical and fluidic interconnections. *IEEE Trans. Compon. Packag. Manuf. Technol.* **3**, 221–228 (2013).
35. Parekh, M. S. et al. High-performance interconnects for 3D ICs using through-silicon vias. In *2011 IEEE 61st Electronic Components and Technology Conference* (ECTC) 1992–1998 (IEEE, 2011).
36. Zheng, L. et al. Novel electrical and fluidic microbumps for silicon interposer and 3-D ICs. *IEEE Trans. Compon. Packag. Manuf. Technol.* **4**, 777–785 (2014).

37. Ramesham, R. et al. Challenges in interconnection and packaging of microelectromechanical systems (MEMS). In *2000 Proceedings. 50th Electronic Components and Technology Conference (Cat. No. 00CH37070)* 666–675 (IEEE, 2000).
60. Savidis, I. et al. Electrical modeling and characterization of through-silicon vias (TSVs) for 3-D integrated circuits. *Microelectron. J.* **41**, 9–16 (2010).

Comment #2. Can the authors provide actual images of the folded regions in Fig. 1 and 2 in addition to the illustrations?

Our Response:

Thank you for your helpful suggestion. In response to the reviewer's comment, we have updated Figure 2 to include actual images of the folded regions for the optical, chemical, and electrical modules**.** In Figure 1, we have further emphasized the explanation of the entire folded system through detailed illustrations. As the actual images of both the chip-integrated side and the skin-interfaced side (before and after folding) are presented in the Supplementary Fig. 1, we have kept Figure 1 focused on illustrations and a view of the fully integrated device.

Our modification to the manuscript:

We have changed the Fig. 2b to actual images of the folded regions showing electrical, optical, and chemical modalities, and modified the associated caption:

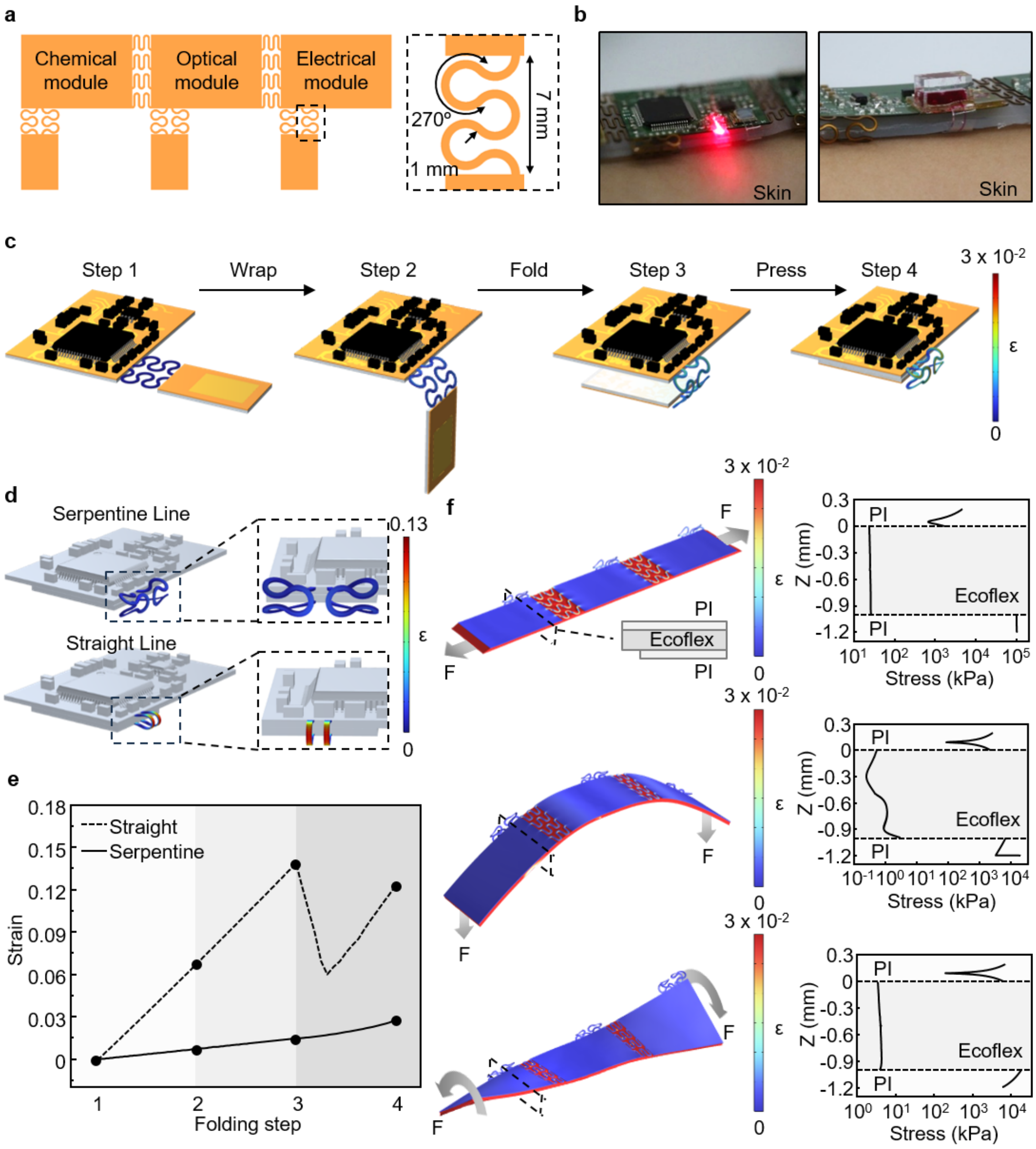

Figure 2. Design and Mechanical Properties of the fMMD. (a) Schematic design of the island-bridge substrate. Chemical, optical, and electrical modules are attached on the substrate, which ensures the structural stability through serpentine interconnects (black dashed box). **(b)** Photos of the fMMD attached on the skin representing different modalities. **(c)** Folding process of the substrate with a module and FEA simulation results of strain on the serpentine interconnects for each step. **(d)** Mechanical analysis of the principal strain in folded state for interconnects designed with serpentine lines versus straight lines. **(e)** Comparison of maximum strain on serpentine and straight interconnects during the folding process. **(f)** Strain and stress of fMMD under multi-deformation modes (stretching, bending, twisting). Strain-isolating effect observed for the deformations applied to the skin-interfaced layer through stress analysis at a cross-sectional plane (black dashed box).

Also, we have updated the text in Page 8:

The overall layout implementing the island-bridge structure with serpentine interconnects is illustrated in Fig. 2a. The chip-integrated substrate is designed to attach sub-systems, including biosensing modules, drug delivery module, and BLE communication modules. The optical image of the entire fMMD with these sub-systems in folded state is shown in Fig. 2b. As previously mentioned, the layout with the sub-systems attached integrates strain-isolating layers in both the chip-integrated layer (non-skin-contact side) and the skin-interfaced layer (skin-contact side).

Comment #3. It is unclear from Fig. 1 how the device is powered as the block diagram simply indicates "power" and the LiPo battery is not shown in the illustration.

Our Response:

We would like to thank the reviewer for this comment regarding to the power supply and management. The fMMD operates using a rechargeable LiPo battery. The LiPo battery and the power management IC units for charging, regulation, and switching are integrated and mounted on a single island within the device. In response to the reviewer's comment, we have revised Figure 1a and 1c accordingly. In Figure 1a, we add the LiPo battery in the illustration, and in Figure 1c, the term "power" has been updated to "power module" to more accurately represent the integration of the various components responsible for power supply and management. Additionally, we have provided a detailed explanation of the power module in the manuscript to clarify its role in the device's operation.

Our modification to the manuscript:

In Figure 1a, we add the LiPo battery in the illustration as follows:

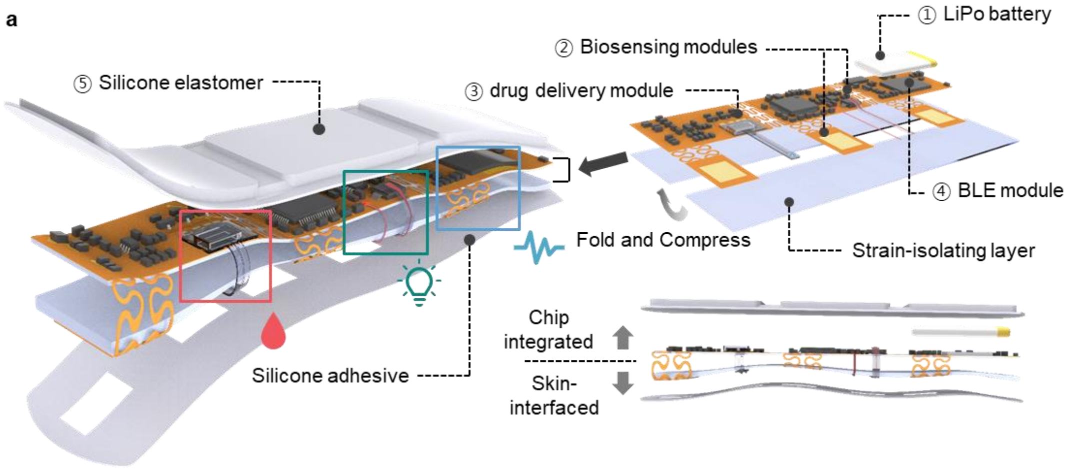

In Figure 1c, we modify the expression of "power" to "power module"

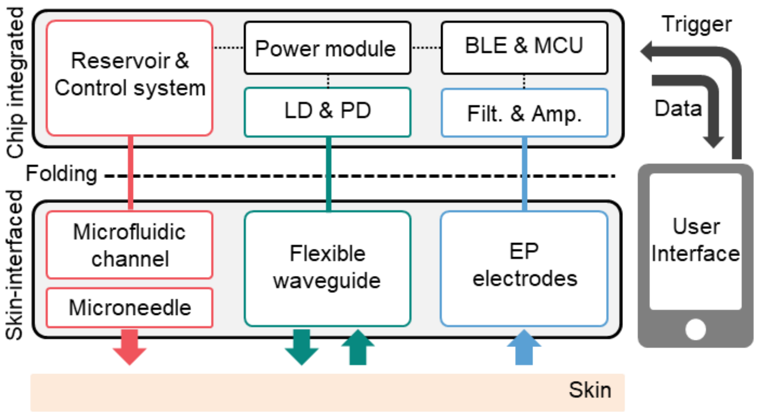

In Page 7, we add the detailed explanations for the power module.

The rigid, thick components are placed on chip-integrated side and the soft, thin components are placed on skin-interfaced side as shown in Fig. 1c. Each modality is integrated with a single platform (Supplementary Fig. 1a), and the folded configuration serves to define sections separating the elements that are in contact with the skin from those that are not (Supplementary Fig. 1b, c). Each functionality can be powered by a power module, which consists of a rechargeable LiPo battery, power switching circuitry, a voltage regulator, and a battery charger IC. The battery can be recharged through a USB port, ensuring continuous operation of the device.

Comment #4. Could the authors add references at the end of this sentence? "As an alternative to via-hole process, a method has been developed that 89 fabricates overall systems in a single layer and then folds them at once." (Line 88)

Our Response:

Thank you for your valuable feedback and the recommendation to include recent references in the folded system. We sincerely appreciate your insight into enhancing the comprehensiveness and relevance of our manuscript. In response to your suggestion, we have added the following references to our manuscript.

Related references

1. Yang, S. M., et al. Soft, wireless electronic dressing system for wound analysis and biophysical therapy. *Nano Today*. **47**, 101685. (2022)
2. Jeong, H., et al. Differential cardiopulmonary monitoring system for artifact-canceled physiological tracking of athletes, workers, and COVID-19 patients. *Science advances*. **7**, eabg3092. (2021)
3. Chung, H. U., et al. Skin-interfaced biosensors for advanced wireless physiological monitoring in neonatal and pediatric intensive-care units. *Nature medicine*. **26**, 418-429. (2020)

Our modification to the manuscript:

In Page 5, we have added the references:

As an alternative to via-hole process, a method has been developed that fabricates overall systems in a single layer and then folds them at once[38-40].

In Page 29, we have added the references:

38. Yang, S. M., et al. Soft, wireless electronic dressing system for wound analysis and biophysical therapy. *Nano Today*. **47**, 101685. (2022)
39. Jeong, H., et al. Differential cardiopulmonary monitoring system for artifact-canceled physiological tracking of athletes, workers, and COVID-19 patients. *Science advances*. **7**, eabg3092. (2021)

<u>Comment #5.</u> For the PPG sensor design in Fig. 4, there is potential concern that movement changes in the bending radius and introduces artifacts in the amplitude profile of the signal.

Our Response:

We sincerely appreciate the reviewer's constructive feedback. Following the reviewer's suggestion, FEA was conducted to examine transmittance when the device undergoes deformation due to movement while attached to the skin. Potential shape changes resulting from movement include variations in bending radius and shearing displacement. Given the device design, the bending radius is expected to remain relatively stable in the free-standing form once adhered to the skin. Therefore, variations of ±4 mm from the current bending radius were analyzed. Also, for potential misalignment due to shearing deformation between the laser-coupled and skin-attached portions of the waveguide, lateral displacements of 0.5 mm, 1.0 mm, and 1.5 mm were evaluated, which is the shearing deformation conditions of 100 %, 200 %, and 300 %, respectively. The results indicate that, even under these mechanical deformations, the normalized transmittance decreases by less than 15% compared to the undeformed state. These FEA results confirm that the amplitude remains sufficient for reliable heart rate and pulse arrival time (PAT) measurement using the PPG sensor.

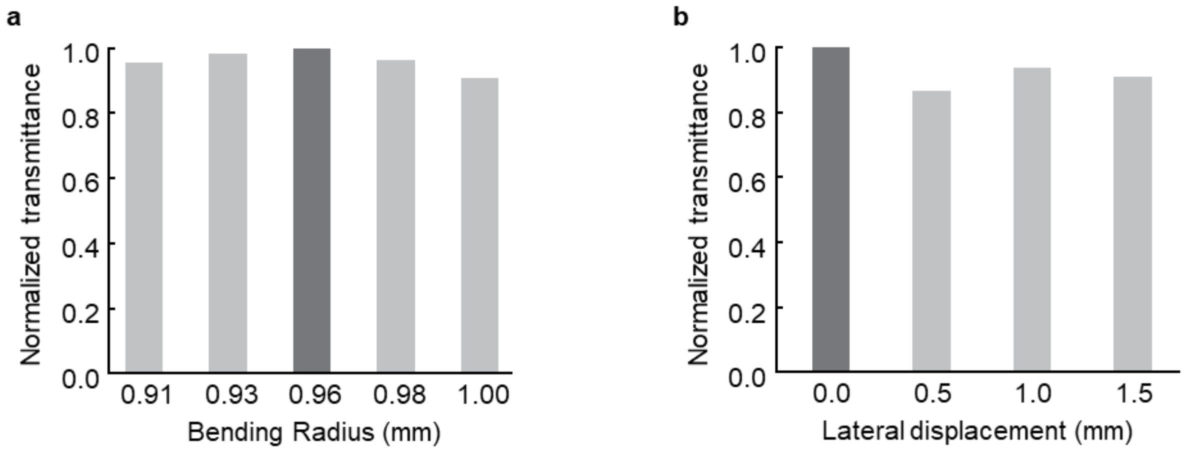

Additionally, we conducted further experimental analysis on PAT changes before and after exercise. Specifically, the real-time blood pressure monitoring function was analyzed by observing increases in heart rate and blood pressure due to vascular responses under both desk work and post-exercise conditions. These additional experiments confirmed the feasibility of PPG measurements. However, for SpO_2 measurements that do not rely on peak detection, amplitude changes may affect accuracy. Therefore, additional post-processing or modifications at the system level may be necessary to address this issue.

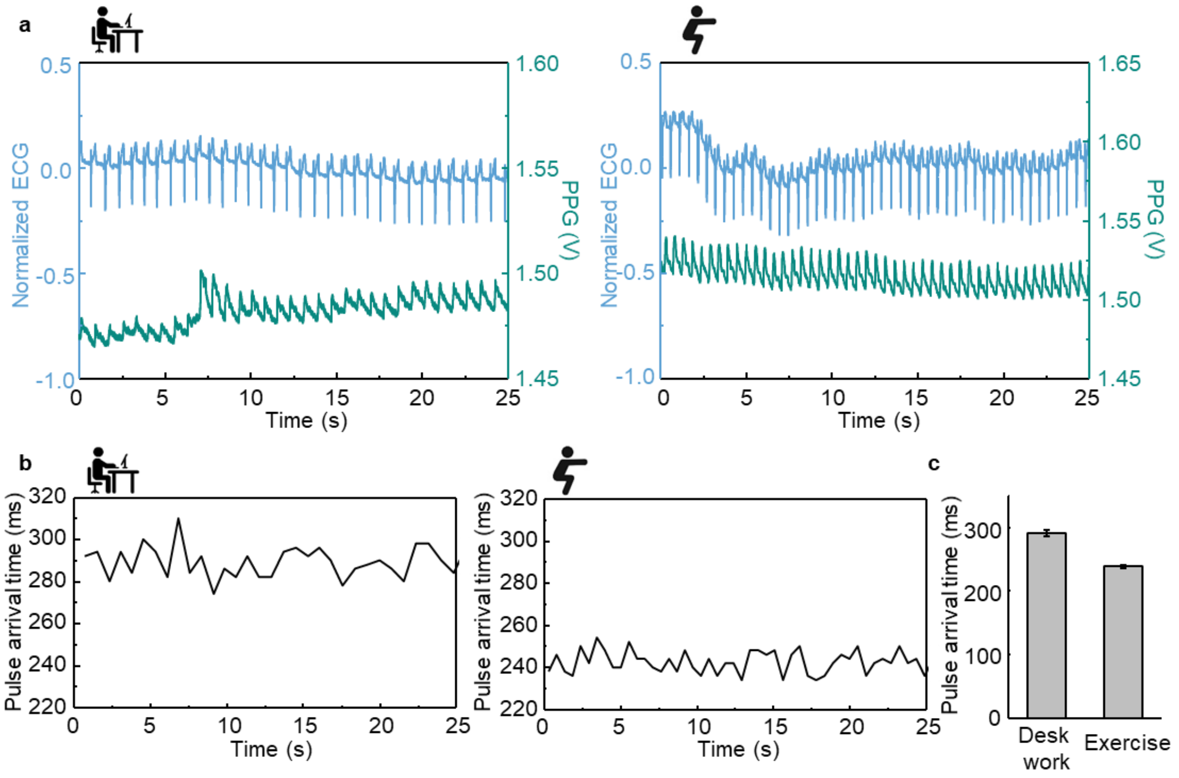

Based on these results, we anticipate that our designed PPG sensor will maintain reliable performance in measuring heart rate and PAT, despite potential deformations caused by movement. This suggests practical applications for continuous health monitoring in daily life settings.

Our modification to the manuscript:

We have added the supplementary Fig.11, 15:

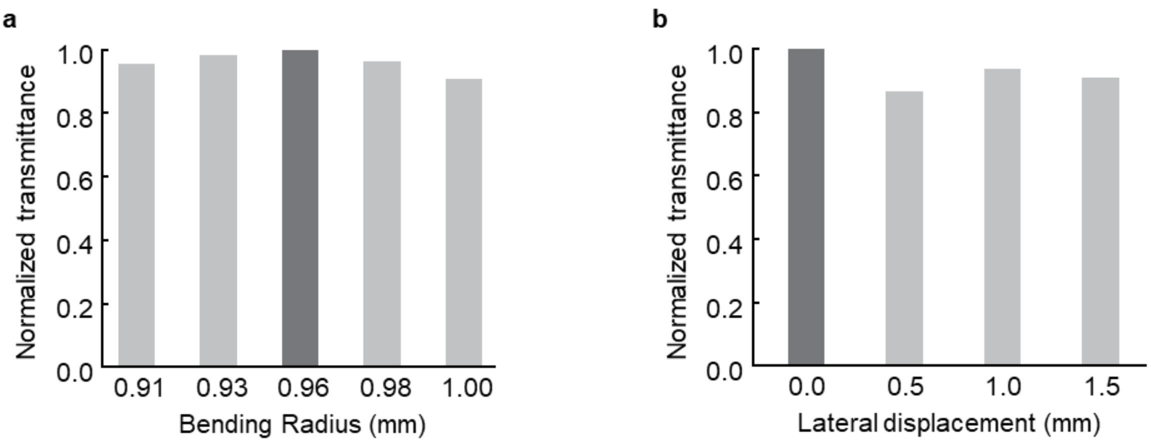

Supplementary Fig. 11 | Transmittance comparison of flexible PDMS waveguide under mechanical deformation using finite element analysis (FEA). (a) Normalized transmittance versus bending radius of flexible PDMS waveguide. A dark gray box represents the

undeformed state of the folded device. **(b)** Normalized transmittance versus lateral displacement of flexible PDMS waveguide due to shearing deformation. The waveguide attached to the skin is laterally displaced relative to the waveguide connected to the laser. A dark gray box represents the undeformed state of the folded device.

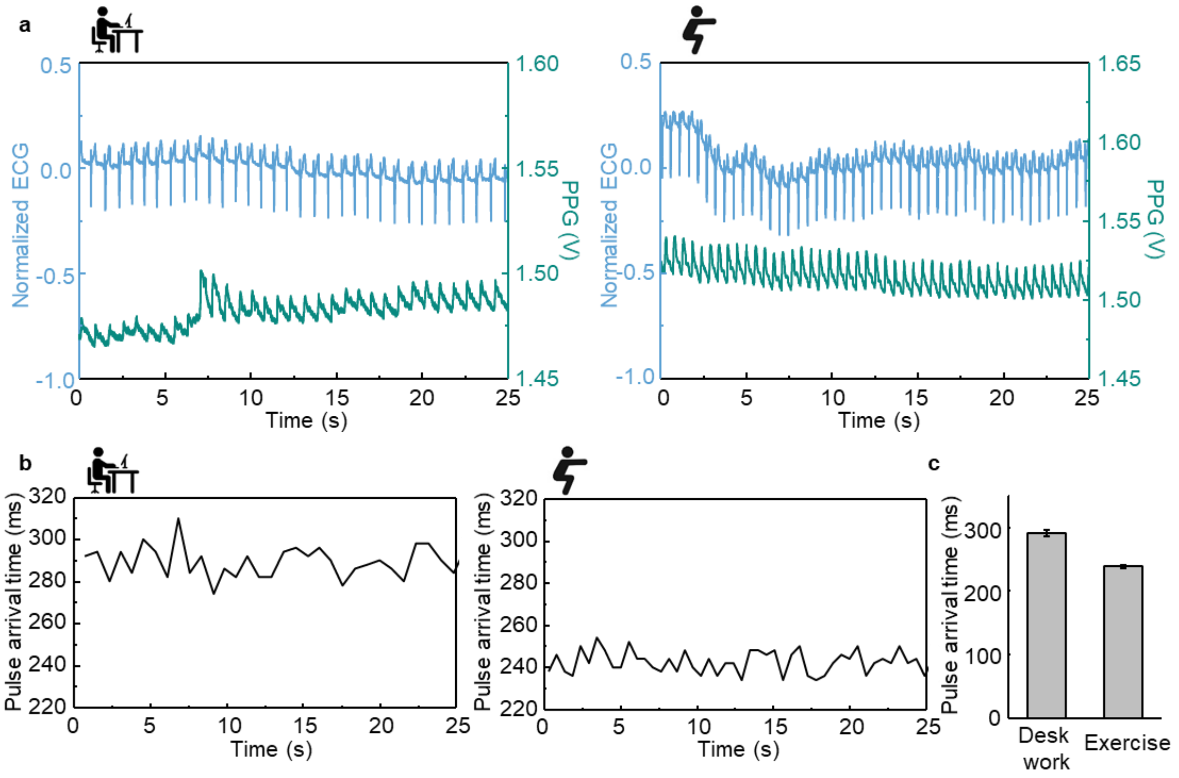

Supplementary Fig. 15 | Comparison of changes in pulse arrival time over time before and after exercise. (a) Real-time measurement with optical and electrical modalities during desk work and after squat workout. (b) Calculation of PAT over time for two activities (desk work and exercise). (c) Comparison of average PAT for measuring time (3 trials).

In Page 15, we have updated the text about Supplementary Fig. 11 in Result section as follows:

Although there is some loss of guiding light due to folding, the transmittance remains above 70 %, which is sufficient to monitor the PPG signal (Fig. 5e). Additionally, supplementary Fig. 11 examined the effects of potential shape deformations on transmittance when the device is attached to the skin during movement. The FEA analyzed transmittance variations resulting from changes in bending radius (±4 mm from the initial radius) and lateral displacements (0.5 mm, 1.0 mm, and 1.5 mm) between the skin-adhered lower waveguide channel and the laser-coupled upper channel. Under these mechanical deformations, the normalized transmittance decreases by less than 15% compared to the undeformed state when the device is attached to the skin.

<u>Comment #6.</u> Fig. 5. Was a pharmaceutically active drug actually delivered to the subject? If so, is there a measured effect? If not, it should be clear in the caption/Methods section that this is a sham drug.

Our Response:

Thank you for the insightful comment. We confirm that a pharmaceutically active drug was not delivered in this study; instead, a sham drug was used to validate the delivery mechanism showing the feedback operation. We have clearly specified this as a sham drug in the Figure 5 caption and the Methods section to avoid any confusion.

Additionally, regarding the potential applications of the drug delivery platform, we have expanded on this in the Discussion section. Based on the device's demonstrated ability to detect cardiovascular biomarkers, including real-time pulse arrival time (PAT) for blood pressure estimation and heart rate variability (HRV) for autonomic function assessment, we believe it could be adapted for applications involving blood pressure regulation through targeted drug delivery. For instance, the platform could be used to deliver antihypertensive agents such as sodium nitroprusside (SNP), which is commonly used for rapid blood pressure reduction in hypertensive crises. This capability would make the device a valuable tool for managing cardiovascular diseases by enabling on-demand drug administration based on real-time biosensing feedback.

Our modification to the manuscript:

In Page 27, we have updated the methods section as follows:

Data analysis and algorithms for multiplexed measurement and closed loop operation

For real-time measurement, the fMMDs were attached on human subject's chest and wrist, respectively. Chest unit measured the ECG, skin temperature, and acceleration at a sampling rate of 500 Hz and wrist unit operated the PWM to trigger LD and measured PPG signal through a series of analog signal processing including analog filters, inverting amplifiers. The collected signals were digitized with 32-bit ADC and transmitted to the personal laptop. Signal processing used a detrend algorithm to remove baseline wander using analysis software (MATLAB R2023b). Peak detection algorithm acquired peak-to-peak time interval for ECG and PPG signals. PAT was calculated from the time intervals between simultaneously obtained ECG and PPG signal waveforms. In addition, the collected ECG signal from chest unit was used to calculate heart rate in real time, utilizing a window size of 5 s. For HRV calculation, a sliding window with a size of 5 s and an overlap of 1 s was applied to the ECG signal, using the intervals between peaks to compute HRV. The Root Mean Square of Successive Differences (RMSSD) was employed as the HRV metric, as it is particularly sensitive to high-frequency variations and provides an effective measure of parasympathetic nervous system activity, making it well-suited for assessing short-term changes in autonomic function. The data packet to initiate drug delivery was transmitted wirelessly from the MATLAB to the fMMD when the calculated heart rate exceeds 120 bpm as a way to show a closed-loop system. The feedback-

actuated drug delivery module delivered a sham drug in a reservoir with a switching frequency of 1 Hz, and the micropump behaviour was filmed using an IR thermal camera.

In Page 20, the discussion section has been modified as follows:

In conclusion, our study represents a significant advancement in wearable healthcare technology, offering a closed-loop platform capable of intelligent monitoring and intervention. Leveraging multiplexed biosensing feedback, the drug delivery platform could be adapted for targeted blood pressure regulation through the administration of antihypertensive agents, such as sodium nitroprusside (SNP), enhancing its utility in managing cardiovascular conditions. Its potential applications extend beyond cardiovascular monitoring to include the management of blood glucose, pain, and various chronic diseases.

We have modified the Fig.5i caption as follows:

(i) IR thermal images of the fMMD before and after sham drug delivery trigger.

Reviewer# 2

This authors present a method for multimodal skin patch integration, which is folding to connect the rigid electronic components and the soft skin. The method is simple but effective, and the study of the effect of folding is comprehensive from both simulation and experiment on mechanical, electrical, optical, and microfluidic aspects. The sensing-stimulated drug delivery device is well-designed and has large application potential. The manuscript is well organized and the expression in both text and figures are clear. However, some of the application demonstrations still require more in-depth and detailed discussion and explanation, and the following points should be addressed before being considered for publication.

Our response:

We sincerely appreciate the reviewer's careful evaluation of our manuscript. Your recognition of the significance of our research and your willingness to provide constructive feedback are highly valued. We have thought to do our best to address the questions you have raised and make the necessary revisions to ensure the quality and completeness of our work before resubmitting the manuscript. Your insights will undoubtedly contribute to further improving and strengthening our research. Thank you for your time and attention to our work, and the technical questions have been addressed in the following discussions.

Comment #1. Page 4 line 83, the authors stated that the via-hole process has been predominantly used. A few references on the via-hole connected biomedical devices should be provided.

Our Response:

We are grateful for the suggestion you have provided to enhance the quality of the manuscript. The via-hole process has been widely used not only in single-module devices, such as displays and epidermal sensors, but also in system-level circuits to create double-sided or multilayered structures. This process involves several techniques, including manually drilling holes and printing conductive materials around them, or layering dielectric materials or substrates, covering conductive traces while leaving small holes, and then printing additional conductive layers around the via-holes. In particular, the stacking of system-level circuits through via-holes or vertical interconnect access (VIA) has been extensively reported in the past five years. In response to your suggestion, we have added the recent works as references related with via-hole process for double-sided or multilayered structure as follows:

Related references

1. Huang, Z. et al. Three-dimensional integrated stretchable electronics. *Nat. Electron.* **1**, 473-480 (2018)
2. Song, H. et al. Highly-integrated, miniaturized, stretchable electronic systems based on stacked multilayer network materials. *Sci. Adv.* **8**, eabm3785 (2022)
3. Li, J. et al. Vertically stacked skin-like active-matrix display with ultrahigh aperture ratio. *Light. Sci. Appl.* **13**, 177 (2024)
4. Zhang, T. et al. A new printed electronics approach eliminating redundant fabrication process of vertical interconnect accesses: building multilayered circuits in porous materials. *Adv. Mater. Technol.* **3**, 4 (2018)

Our modification to the manuscript:

In Page 4, we have updated the text:

As one of the integration methods, stacking through via-hole processes have been predominantly used[29-32]. Reliable methods for via-hole fabrication and filling have been well-established for conventional electronics;

In Page 29, we have added the references:

29. Huang, Z. et al. Three-dimensional integrated stretchable electronics. *Nat. Electron.* **1**, 473-480 (2018)

30. Song, H. et al. Highly-integrated, miniaturized, stretchable electronic systems based on stacked multilayer network materials. *Sci. Adv.* **8**, eabm3785 (2022)
31. Li, J. et al. Vertically stacked skin-like active-matrix display with ultrahigh aperture ratio. *Light. Sci. Appl.* **13**, 177 (2024)
32. Zhang, T. et al. A new printed electronics approach eliminating redundant fabrication process of vertical interconnect accesses: building multilayered circuits in porous materials. *Adv. Mater. Technol.* **3**, 4 (2018)

Comment #2. What is the necessity of the folding? A single layer with a soft substrate without folding can also realize the functions. It is important to clarify this point in the manuscript to strengthen the creative point.

Our Response:

We sincerely appreciate the reviewer's insightful comment on the necessity of the folding structure, and we agree that clarifying this aspect will strengthen the creative point of our research. As the reviewer pointed out, it is possible to design a system-level multimodal device by placing rigid components, such as IC chips and battery on an FPCB, directly onto a soft substrate, and positioning skin-interfaced sensors or microneedle alongside them. Due to the necessity of the skin attachment of sensing electrodes or transdermal drug delivery modules, entire system components can't mount on a soft substrate if it has a single layer configuration. Without folding process, these two separate components can be electrically connected using methods such as anisotropic conductive films, magnets, or conductive epoxy, and many studies using single-layer approaches have been reported[1-3]. These approaches may address some of the issues related to mechanical strain on the rigid components, but it would still have some limitations compared to a folded structure.

First, stress distribution is key challenges in a single-layer design. Placing soft substrate under rigid IC chips can reduce the direct impact of skin movements on these components, offering some mechanical cushioning. However, this approach does not entirely mitigate the stress concentration problem, especially at the interfaces between the FPCB and the flexible parts of the device (e.g., sensors or drug delivery modules). The transition region where the rigid and flexible sections meet induces strain concentrations, which can lead to cracking, delamination, or failure in those areas. This remains a concern because the entire device will still undergo uniform bending or stretching, and the soft substrate alone cannot fully distribute the stresses across the system, thereby increasing the likelihood of potential electrical disconnection or performance degradation. In contrast, the folded structure allows for better distribution of mechanical stress across different functional layers, with the folds acting as buffers to absorb strain and protect the rigid components.

Second, mechanical adaptation to skin movement is limited in a single-layer design. Due to the non-uniform stress distribution, rigid components may resist skin deformations, causing discomfort and reducing the device's mechanical reliability over time. A folded structure, however, isolates the rigid components within the folds, allowing only the flexible components to interact with the skin. This results in improved adaptability to dynamic movements and better long-term wearability.

Lastly, the spatial efficiency provided by the folding structure is another advantage. the area required for each additional modality increases linearly within the same plane. As more functions—such as different sensor modalities or drug delivery mechanisms—are added, the device grows larger, making it less compact and more challenging to wear comfortably. In contrast, the folding structure offers a significant advantage by enabling the integration of multiple components within a smaller, more compact area. By utilizing vertical space, the

folding structure allows the various sensor and drug delivery modules to be distributed across different folds, effectively minimizing the device's footprint without sacrificing functionality. This design ensures that all necessary functions can be implemented efficiently while maintaining a small form factor.

In summary, while single-layer approaches are feasible, the folded structure offers superior stress management, mechanical adaptability, electrical reliability, and spatial efficiency, all of which are crucial for the wearable performance and multifunctionality of the device. Thus, based on the reviewer's comment, we have revised the manuscript to clarify these points and emphasize the creative significance of the folding structure.

Related references

1. Ciui, B. et al. Wearable wireless tyrosinase bandage and microneedle sensors: toward melanoma screening. *Adv. Healthc. Mater*. **7**, 1701264 (2018)
2. Kim, J. et al. Simultaneous monitoring of sweat and interstitial fluid using a single wearable biosensor platform. *Adv. Sci.* **5**, 1800880 (2018)
3. WANG, Y. et al. Wearable plasmonic-metasurface sensor for noninvasive and universal molecular fingerprint detection on biointerfaces. *Sci. Adv.*, **7**, eabe4553 (2021)

Our modification to the manuscript:

In Page 4, we have added the text:

Although many ultrathin and flexible devices have been developed to enable conformal contact[27,28], expanding those to a system-level entails incorporating various components such as power management, communication modules, signal processing modules, and drug loading chambers. Attempting to incorporate these bulky and rigid components alongside sensors and drug delivery units into a single-layer structure on a soft substrate can lead to significant challenges. For instance, due to skin deformation, such a configuration can create stress concentration at the interfaces between rigid and flexible components, increasing the risk of device delamination. This instability negatively impacts the quality of sensed data and the effectiveness of drug delivery by disrupting the device's intimate contact with the skin. To address these issues, a multi-layer approach is essential, as it allows for more effective integration and stress management across different layers. Multilayered structures can distribute mechanical stress more evenly, improve mechanical adaptation to dynamic skin movements, and enhance spatial efficiency rather than spreading them in a single plane. As one of the integration methods, stacking through via-hole processes have been predominantly used[29-32].

Comment #3. This work demonstrates the superiority of folded structures for integrating multifunctional components from a mechanical stability perspective using finite element analysis, but does not discuss the electrical reliability of biosensing and signal feedback under diverse motion states, more electrical characterization (sensitivity and operation stability for practical applications, etc.) should be added.

Our Response:

Thank you for this valuable comment. In response, we conducted additional electrical characterization to evaluate the biosensing reliability and signal feedback under diverse motion states. Specifically, we collected ECG signals and 3-axis accelerometer data while the wearer performed various movements, including sitting-to-standing transitions and walking. As shown in the attached figure, the data confirms successful acquisition of ECG signals and distinguishable 3D reconfigured motion tracking across different activities. This demonstrates that the device maintains stable electrical performance and reliable biosensing even under dynamic conditions. Thus, based on the reviewer's comment, we have revised the manuscript and added the experimental results in supplementary figure.

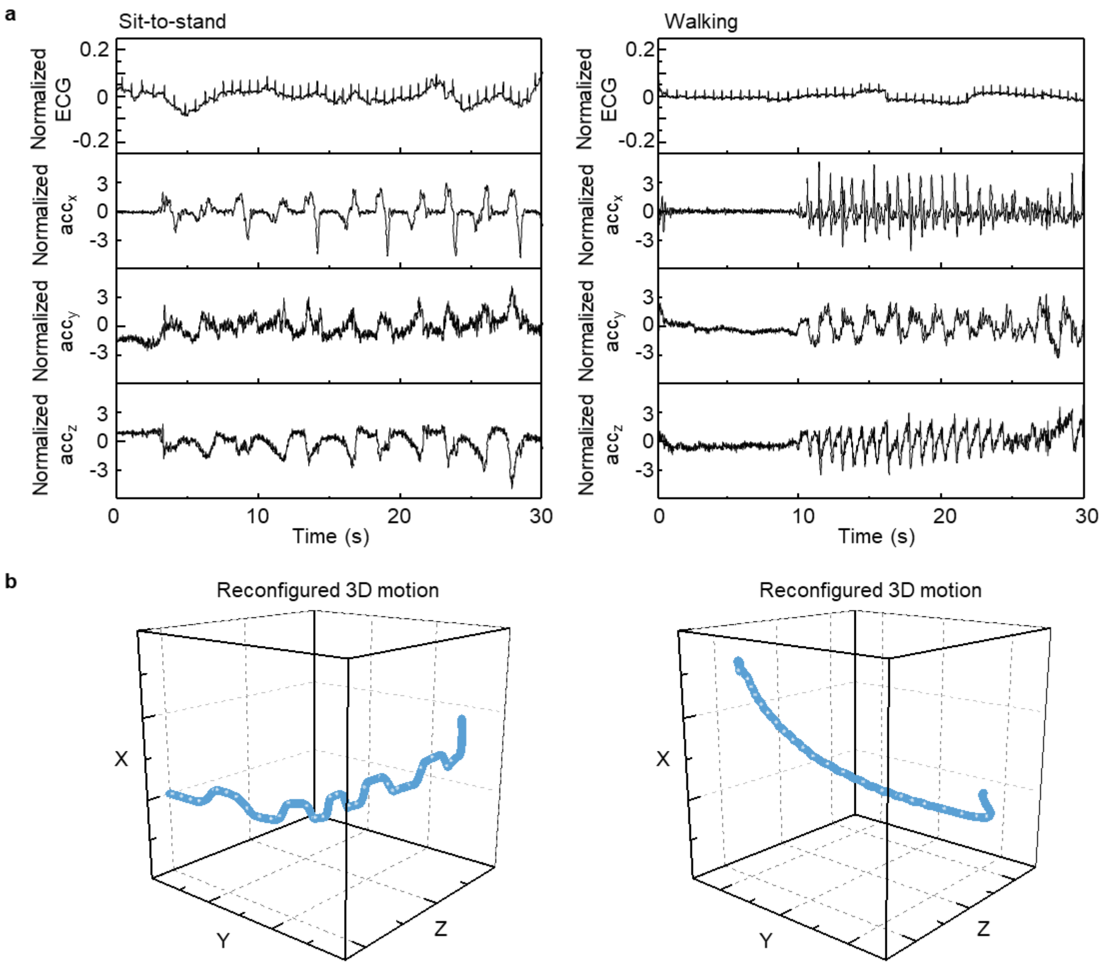

Our modification to the manuscript:

In Page 17, we have revised the manuscript as follows:

Figure 5c shows the various electrophysiological signals, including ECG, skin temperature, and body movement, through an electrical modality of the chest mounted fMMD during the desk working and walking. The folded structure and strain-isolating elastomer allow the fMMD to reliable measurements by ensuring intimate contact with the skin and reducing mechanical stress, allowing stable acquisition of signals such as ECG and 3-axis accelerometer data and tracking three-dimensional reconfigured motion even during activities like sitting-to-standing transitions, and walking (Supplementary Fig. 13).

We have added the supplementary Fig. 13:

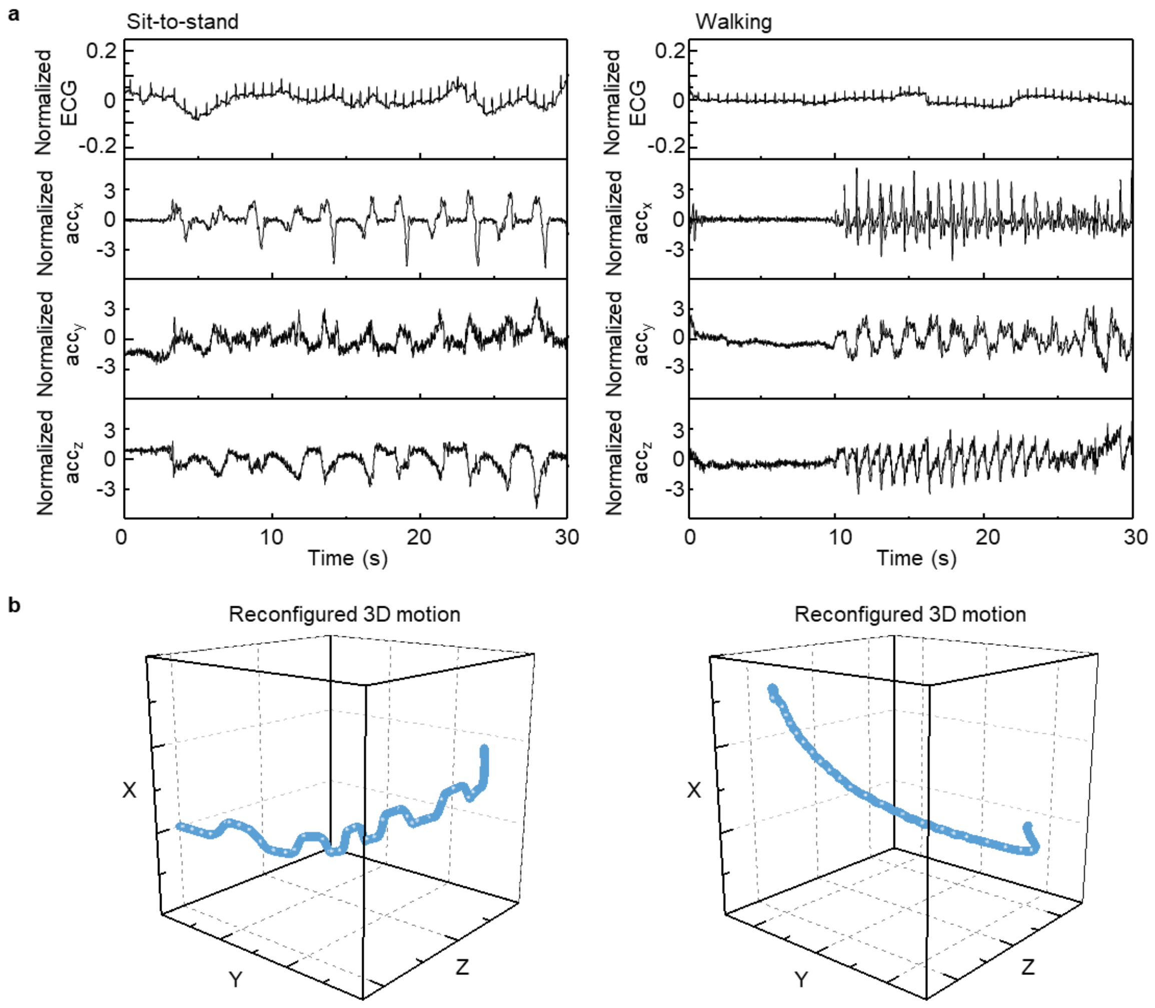

Supplementary Fig. 13 | Electrical reliability of biosensing under diverse motion states. (a) Representative recording of ECG and three-axis acceleration from fMMD attached on the wearer's chest for cases of sit-to-stand and walking. **(b)** Inferred 3D patterns of motion according to measured accelerations.

Comment #4. It is suggested to briefly discuss whether the via-hole process is applied for electrical connection, mechanical alignment, optical pathway, or fluid channel. It is confusing to merge all these functions, since in Line 86, the conductive paste is applied in the via-hole, and line 87 states the leakage of drug fluid due to misalignment.

Our Response:

We sincerely appreciate the reviewer's constructive feedback. Following your suggestion, we have expanded the discussion to further explore research involving the application of via-hole structures. Currently, via-hole structures in wearable devices are primarily focused on electrical connections[1-3]. While conventional electronic devices have established reliable methods for via-hole fabrication and filling, such methods are limited when applied to flexible and stretchable printed electronics[2]. This limitation arises from the increased process complexity, as well as the challenges posed by alignment mismatches and material damage during fabrication[4]. The conductive paste mentioned in line 86 refers to the high complexity of electrical interconnection processes, while line 87 discusses issues related to fluid leakage that can occur due to alignment mismatches and material damage.

Via holes vary structurally depending on their application, which significantly increases process complexity. For example, Through Silicon Via (TSV) is used for electrical connections[5-7], optical pathways[8, 9], and fluid channels[10, 11]. In one instance, TSV-based stacked SiP (System-in-Package) technology was utilized to fabricate a 10-layer stacked chip[6], demonstrating a liquid cooling solution incorporating both Cu TSVs and fluidic TSVs[7]. Furthermore, a 3D packaging solution integrating electrical, optical, and fluidic interconnects has been demonstrated to address challenges in power delivery, high-bandwidth communication, and cooling [8]. As these studies illustrate, vias for electrical, optical, and fluidic interconnects require different fabrication methods. Electrical vias are filled with metal, optical vias use transparent materials such as SU-8, and fluidic vias are left hollow to enable fluid circulation. This diverse approach across modalities contributes to the increased complexity of the fabrication process.

Additionally, via-hole fabrication processes face significant challenges related to alignment mismatches and material damage. Alignment issues are particularly likely when materials with different coefficients of thermal expansion (CTE) are used. Materials commonly employed in wearable devices, such as polyimide, Ecoflex, and PDMS, exhibit differing CTE values, leading to varying degrees of expansion or contraction with temperature changes, which complicates precise alignment during fabrication. These flexible materials may not remain flat during drilling, causing the laser focus to shift, potentially resulting in material damage. In laser drilling-based via-hole processes, the elevated substrate temperature may damage heat-sensitive materials, while etching-based via-hole processes may cause material damage during exposure to solvent-based developers or plasma. Such misalignment and material damage can lead to performance degradation or even fluid leakage in drug delivery systems.

Considering these factors, folding may be a more suitable option than via-hole processes for the production of multimodal devices. The folding process is less complex, allowing for easier

integration of multiple modalities while reducing the risks of alignment errors and material damage. Based on these discussions, we have revised the text to clarify concerns about the via-hole process and avoid confusion regarding its various applications in electrical connections, optical pathways, and fluid channels.

Related references

1. Joo, H. et al. Soft implantable drug delivery device integrated wirelessly with wearable devices to treat fatal seizures. *Sci. Adv.* **7**, eabd4639 (2021).
2. Jansson, E. et al. Development of a full roll-to-roll manufacturing process of through-substrate vias with stretchable substrates enabling double-sided wearable electronics. *Int. J. Adv. Manuf. Technol.* **111**, 3017–3027 (2020).
3. Poliks, M. et al. A wearable flexible hybrid electronics ECG monitor. In *2016 IEEE 66th Electronic Components and Technology Conference (ECTC)* 1623–1631 (IEEE, 2016).
4. Ramesham, R. et al. Challenges in interconnection and packaging of microelectromechanical systems (MEMS). In *2000 Proceedings. 50th Electronic Components and Technology Conference (Cat. No. 00CH37070)* 666–675 (IEEE, 2000).
5. Andry, P. et al. A CMOS-compatible process for fabricating electrical through-vias in silicon. In *56th Electronic Components and Technology Conference* (ECTC) (IEEE, 2006).
6. Jang, D. M. et al. Development and evaluation of 3-D SiP with vertically interconnected through silicon vias (TSV). In *57th Electronic Components and Technology Conference* (ECTC) (IEEE, 2007).
7. Khan, N. et al. 3-D packaging with through-silicon via (TSV) for electrical and fluidic interconnections. *IEEE Trans. Compon. Packag. Manuf. Technol.* **3**, 221–228 (2013).
8. Parekh, M. S. et al. High-performance interconnects for 3D ICs using through-silicon vias. In *2011 IEEE 61st Electronic Components and Technology Conference* (ECTC) 1992–1998 (IEEE, 2011).
9. Karppinen, M. et al. Fabrication and characterization of electrical through-vias in flexible silicon substrates. In *56th Electronic Components and Technology Conference* (ECTC) 7 pp. (IEEE, 2006).
10. Zheng, L. et al. Novel electrical and fluidic microbumps for silicon interposer and 3-D ICs. *IEEE Trans. Compon. Packag. Manuf. Technol.* **4**, 777–785 (2014).
11. Kartalov, E. P. et al. Microfluidic vias enable nested bioarrays and autoregulatory devices in Newtonian fluids. *Proc. Natl Acad. Sci. USA* **103**, 12280–12284 (2006).

Our modification to the manuscript:

In Page 4, we have updated the text:

As one of the integration methods, stacking through via-hole processes have been predominantly used[29-32]. Reliable methods for via-hole fabrication and filling have been well-established for conventional electronics; however, these approaches present limitations when applied to flexible and stretchable printed electronics[33]. Via-hole structures vary in design based on their application (e.g., electrical connections, optical pathways, or fluidic channels), [34-36] requiring precise alignment and integration with different materials, such as conductive pastes or SU-8, which increases process complexity. Furthermore, flexible materials commonly used in wearable devices, such as polyimide, Ecoflex, and PDMS, exhibit different coefficients of thermal expansion (CTE), increasing the risk of alignment mismatches[37]. During the drilling process, these materials are also prone to damage, raising concerns about fluid leakage and device integrity.

In Page 29, we have added the references:

33. Jansson, E. et al. Development of a full roll-to-roll manufacturing process of through-substrate vias with stretchable substrates enabling double-sided wearable electronics. Int. J. Adv. Manuf. Technol. 111, 3017–3027 (2020).
34. Khan, N. et al. 3-D packaging with through-silicon via (TSV) for electrical and fluidic interconnections. *IEEE Trans. Compon. Packag. Manuf. Technol.* **3**, 221–228 (2013).
35. Parekh, M. S. et al. High-performance interconnects for 3D ICs using through-silicon vias. In *2011 IEEE 61st Electronic Components and Technology Conference* (ECTC) 1992–1998 (IEEE, 2011).
36. Zheng, L. et al. Novel electrical and fluidic microbumps for silicon interposer and 3-D ICs. *IEEE Trans. Compon. Packag. Manuf. Technol.* **4**, 777–785 (2014).
37. Ramesham, R. et al. Challenges in interconnection and packaging of microelectromechanical systems (MEMS). In *2000 Proceedings. 50th Electronic Components and Technology Conference (Cat. No. 00CH37070)* 666–675 (IEEE, 2000).

<u>Comment #5.</u> In Figure 1a, the electrical modules cannot match the 5 modules in the main content description. For example, we cannot spot the battery and BLE module in the figure. Similar problem is observed in Figure 1b, where the position LD, waveguide, and photodetector are not clear. Maybe the author can label ① ② ③ according to the description in the main content if the space not enough to label the full names in the figure.

Our Response:

We sincerely appreciate the reviewer's constructive comment to improve the clarity and understanding of the structure of the fMMD. Since the fMMD is a standalone system capable of multimodal biosensing and drug delivery feedback, it contains a wide variety of components. Additionally, as many sub-components are integrated within the major modules, we recognize the importance of providing detailed labels and explanations for each of these elements. Matching the components described in the manuscript with corresponding numbers in Fig. 1a, b will significantly enhance the reader's understanding of the device structure. Therefore, we have labeled the relevant components in Figure 1a with matching numbers and full names, allowing users to easily identify each element and comprehend the overall structure through the figure itself. In conclusion, in response to your helpful comments, we have revised the manuscript and figures accordingly to improve clarity and ensure the correct labeling of all components.

Our modification to the manuscript:

In Page 6-7, we have updated the text:

Figure 1b represents the three modalities for closed-loop system based on simultaneous biosensing and drug delivery. In terms of biosensing, ECG, body movement, and skin temperature are acquired through folded serpentine interconnects (Fig. 1b, right). In addition to electrical signals, PDMS-based flexible optical waveguides are used to measure PPG signal. The rigid and thick components, such as (1) laser diode (LD, 650nm wavelength) and (2) photodetector (PD), are mounted on chip-integrated side, and (3) the two flexible waveguides are well aligned with the light source spot of LD and the light receiving spot of PD using optical epoxy, respectively (Fig. 1b, center). The folded waveguides aligned with the LD and PD serve as guiding the light to the skin and the reflected light from the skin. In terms of controlled transdermal drug delivery, drug delivery system is composed of three functional components (Fig. 1b, left): (1) injection trigger and pumping system with the performance of steady flow rate, (2) drug storage and microfluidic channel for delivery from reservoir to skin, and (3) microneedle array for penetration into the skin.

And, we have modified Figure 1a as follows:

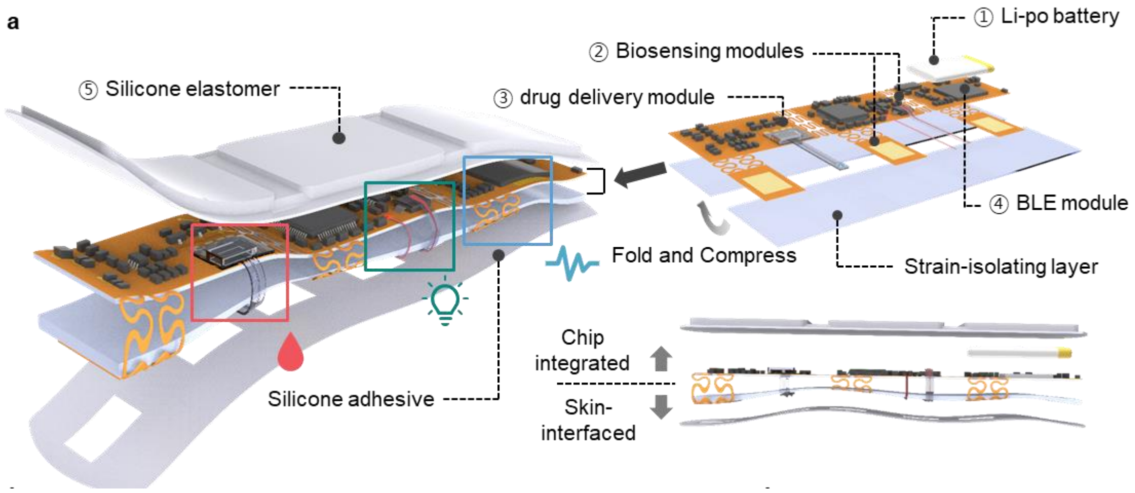

We have modified Figure 1b as follows:

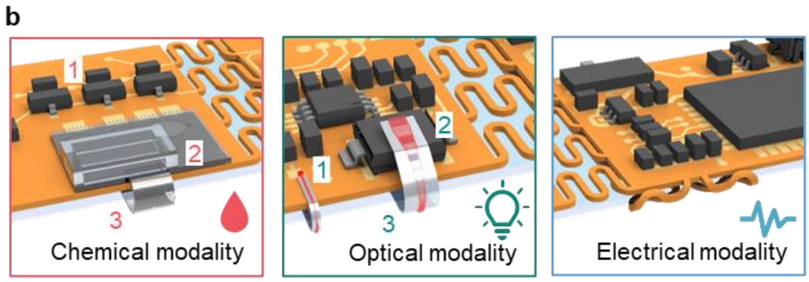

Comment #6. In Figure S2a, the unit seems to be wrong. Curvature radius equals to the reciprocal of curvature, where the unit for curvature radius is mm, and the unit for curvature is mm-1.

Our Response:

We would like to thank the reviewer for this observation regarding to the unit in Supplementary Fig. 2a. The relevant expression has been corrected to mm in Supplementary Fig. 2a.

Our modification to the manuscript:

In Supplementary Fig. 2a, we modify the figure and caption in terms of the expression of unit as follows:

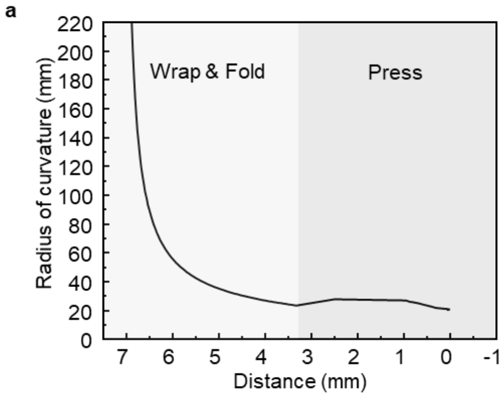

Supplementary Fig. 2 | Mechanical analysis for design optimization of the folded MMD. (a) Graph of radius of curvature versus distance during the folding process of the substrate for the straight line interconnect design. The x-axis represents the distance (mm) between the skin contact side and the non-contact side, while the y-axis indicates the radius of curvature (mm) of the straight interconnect.

<u>Comment #7.</u> The word "polymer gel" is not correct to describe the ecoFlex in the strain-isolating layer. Polymer gel usually indicates a semisolid system in a liquid medium (such as swollen by a solvent).

Our Response:

Thank you for your constructive comment. As the reviewer mentions, we recognize that "polymer gel' is not appropriate, as it typically refers to a semisolid system in a liquid medium. To represent the properties of Ecoflex, the term "polymer gel" has been replaced with "silicone elastomer" in the manuscript.

Our modification to the manuscript:

In Page 6, we modify the term as follows:

A silicone elastomer (500 μm thick) with low Young's modulus (50 kPa) provides strain-isolating effect between skin-contact side and non-contact side to mitigate the stresses at the non-contact side islands for uniaxial stretching of up to 20 %[29].

In Page 9, we modify the term of Ecoflex as follows:

To verify the overall deformability provided by the serpentine interconnect design and the strain isolation effect of the silicone elastomer, FEA was performed.

Also, in Page 10, we modify the term of Ecoflex as follows:

These results confirm that the fMMD's serpentine interconnect design and silicone elastomer layer effectively enhance deformability and provide strain isolation, ensuring stable performance during use.

Comment #8. The heterogeneous integration is important, in which the bonding between soft and rigid surfaces should be better studied. The FEA study in Figure 2f shows the function of the strain isolating layer, but I feel it is not enough. Could the author add a FEA study to show the strain in the heterogeneous device when attached to the skin? e.g. including rigid layer, strain isolating layer, skin, and probably the encapsulation layer. Displacement might be assigned from the skin side, and study the stress and strain on each layer of the device.

Our Response:

We sincerely appreciate the reviewer's insightful feedback. The FEA results presented in Figure 2f analyze the strain and stress on the device when attached to the skin and subjected to deformation, without the inclusion of an encapsulation layer. To focus on the specific area of interest, we post-processed the skin geometry to exclude it from the figure. During analysis, the device was attached to the skin, and then post-processing was applied to display the device as separated from the skin layer in the final visualization.

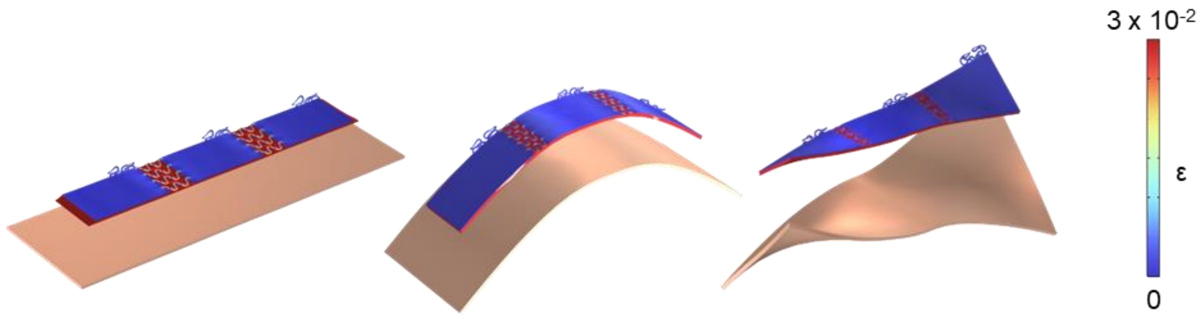

Following the reviewer's suggestion, we conducted additional analysis by adding an encapsulation layer as it would be applied to the actual device, attaching it to the skin, and assessing the strain and stress under these conditions.

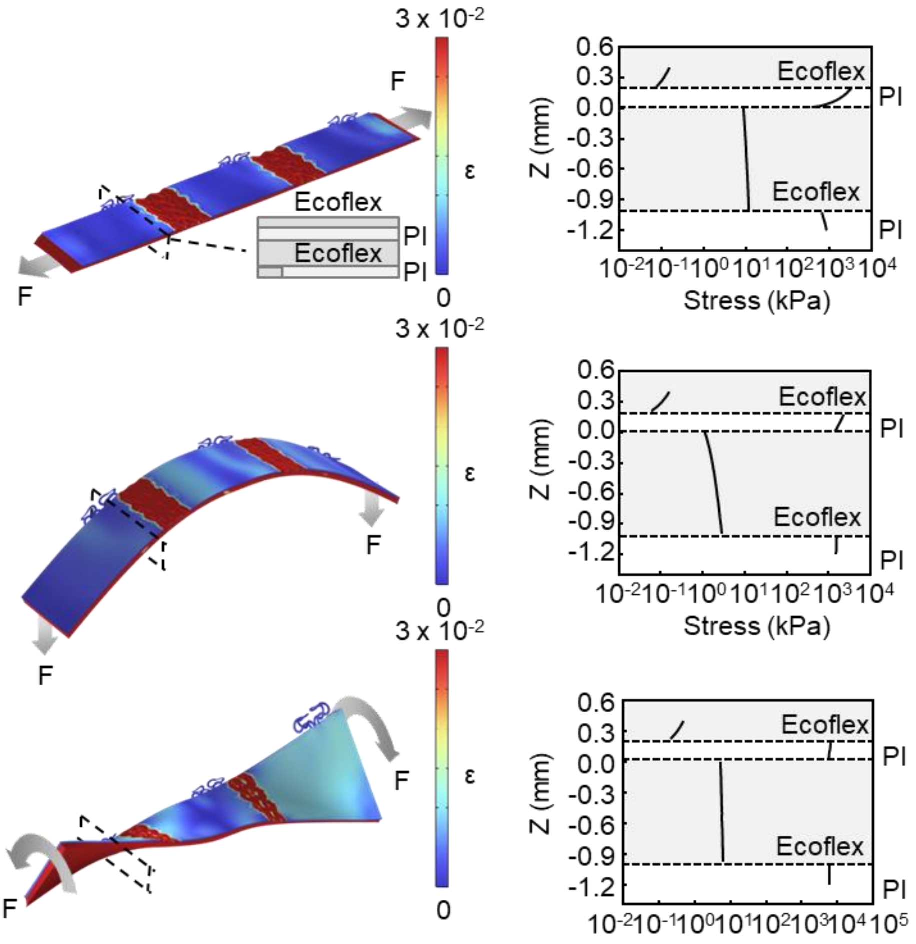

Additionally, we included the FEA analysis without the encapsulation layer in Figure 2 to illustrate that the area where the rigid component is attached experiences no strain when deformation occurs, confirming that there is no performance degradation. The analysis results with the encapsulation layer have been provided as Supplementary Fig. 3. Also, the text has been refined to clarify the analysis method for deformation applied to the skin.

Our modification to the manuscript:

We have added the Supplementary Fig. 3:

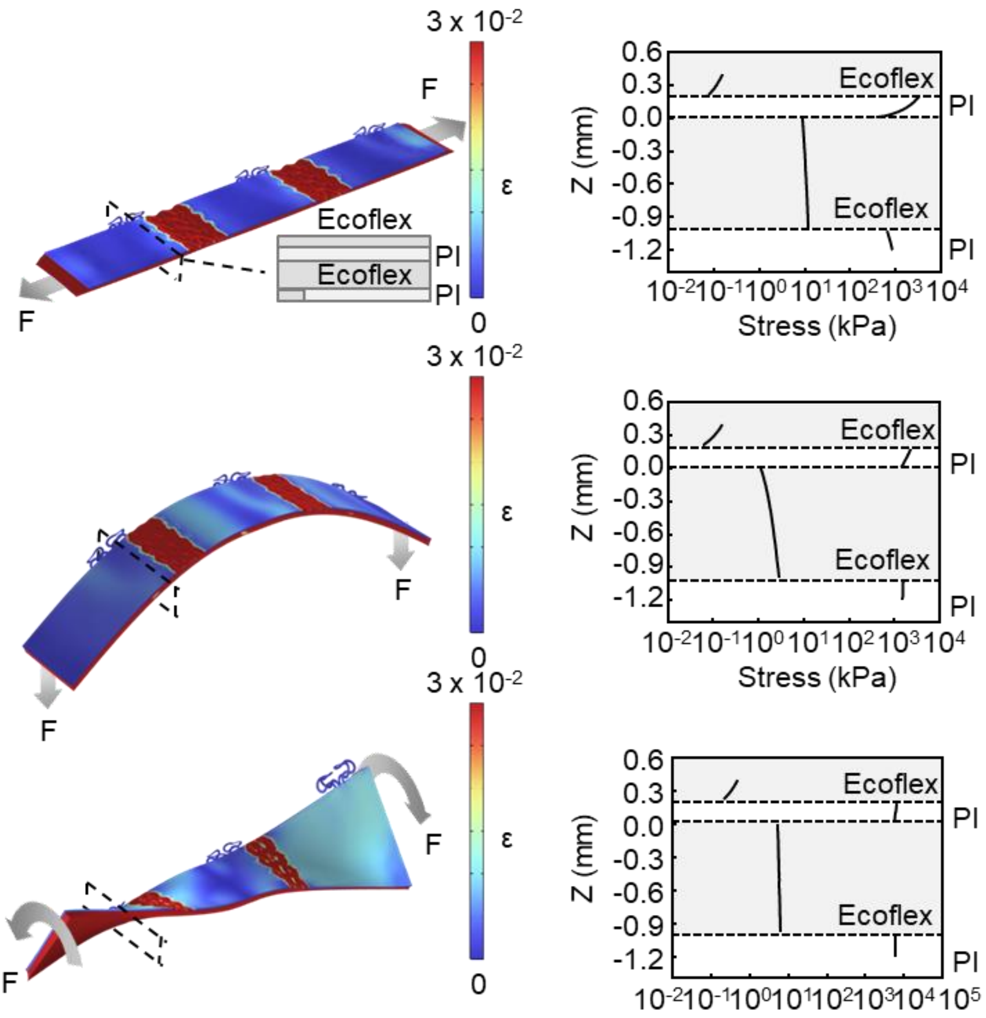

Supplementary Fig. 3 | Mechanical Properties of the fMMD with encapsulation layer. Strain and stress of fMMD with encapsulation layer under multi-deformation modes (stretching, bending, twisting). The strain-isolating effect is demonstrated for deformations applied to the skin-interfaced layer, as shown by stress analysis on a cross-sectional plane (indicated by the black dashed box).

In Page 9, we have updated the text about Supplementary Fig. 3 in Result section as follows:

The strain associated with multi-deformation modes is illustrated, and the stress can be observed in the graph. Similarly, strain and stress analysis for the encapsulated device was conducted under various deformation modes (stretching, bending, and twisting), confirming that the strain in the PI upper layer, where the actual chip is integrated, remains within 0.03 in both the presence and absence of the encapsulation layer(Fig. 2f and Supplementary Fig. 3).

In Page 25, we have updated the text about Fig 2f. and supplementary Fig. 3 in Method section as follows:

Strain was analyzed at each stage of folding process and various deformation modes. The devices with and without an encapsulation layer were laminated onto the skin and subjected to various deformations. The strain and stress of the devices were then analyzed under these skin deformations (stretching, bending, and twisting). After the analysis, the skin topography was post-processed to ensure it was not visible.

Comment #9. Since the whole device is encapsulated with a white color polymer, how can we determine when the drug has been used up? And how to top up the drug?

Our Response:

We sincerely appreciate the reviewer's thoughtful question regarding drug depletion and refilling in the encapsulated fMMD. We agree that determining the drug's usage status and simplifying the refilling process are important considerations. As the reviewer noted, the system is entirely encapsulated in a white-colored polymer, which prevents direct visual confirmation of drug depletion. Alternatively, the tracking the duration of the pumping actuation can be used as an indirect method for monitoring the drug status. The point at which the drug is nearly depleted can be inferred by correlating the pumping frequency, the flow rate of each drug delivery operation, and the total capacity of the drug storage chamber. This method provides an estimation based on the operational parameters of the micropump.

Regarding the drug loading process, the reservoir is manufactured by pouring PDMS into a mold and curing it, with a total capacity of 21 μL. To ensure that the pressure from the micropump is fully transferred to the microfluidic channel without pressure leakage, the reservoir is designed to have a completely sealed structure. Because of this, the drug is filled by inserting a 27-gauge syringe into the top of the PDMS reservoir. Due to the elasticity and self-healing properties of PDMS—with a thickness of 3 mm or more—the reservoir reseals itself completely when the syringe is removed after refilling.

However, these indirect monitoring methods and reservoir designs still have limitations in terms of accurate and convenient drug management. To enhance user interaction and improve drug monitoring, the inferred amount of drug used could also be displayed graphically on a user interface (UI). This approach would allow users to monitor the drug usage visually, offering an intuitive way to track the remaining drug amount. In addition, by attaching a septum-structured refill port to the area where the reservoir is located in the encapsulated device[1], convenient reloading can be achieved through an externally exposed refill port structure, while maintaining the device's sealed integrity.

Related reference

1. Forouzandeh, F. et al. A Wirelessly Controlled Scalable 3D-Printed Microsystem for Drug Delivery. *Pharmaceuticals.* **14**, 6 (2021)

Comment #10. The authors utilize a folded structure with serpentine interconnections to guarantee seamless integration of multiple sensing modalities for real multi-pathway biosensing and on-demand drug delivery. However, a great deal of real-time monitoring of biosignals and qualitative analysis is absent in practical applications. Possible solutions to achieve a better design of the sensing-delivery-feedback platform to tackle the point should be provided and discussed.

Our Response:

Thank you for your insightful comment. We agree that real-time monitoring and qualitative analysis of biosignals are crucial for improving the functionality of the biosensing-delivery-feedback platform. Currently, our system is focused on biosensing, which can analyze a combination of electrophysiological and optical-based pulse wave signals. Further approaches to advance the current fMMD can be summarized in three approaches: 1) Advanced electrophysiological analysis, 2) Integration of electrochemical sensing modality, and 3) Development of effective micropump for acute care.

Advanced electrophysiological analysis, including metrics like heart rate variability (HRV)[1], blood pressure[2], and arrhythmia detection through Extended Continuous Rhythm Monitors (ECRM)[3] such as Holter monitors, plays a crucial role in monitoring and diagnosing a variety of cardiovascular and autonomic nervous system disorders. HRV analysis, which evaluates the fluctuations in the time interval between heartbeats, provides valuable insights into the balance between the sympathetic and parasympathetic nervous systems. Low HRV is associated with conditions like cardiovascular disease, diabetes, and stress-related disorders, indicating impaired autonomic regulation. Blood pressure monitoring, particularly through continuous wearable devices, is beneficial for managing hypertension and detecting hypertensive episodes, which can prevent complications such as stroke, heart attack, and kidney damage. ECRM can enhance arrhythmia detection by recording heart rhythm over longer durations than traditional ECGs, which is especially useful for capturing infrequent or asymptomatic arrhythmic events like atrial fibrillation, premature ventricular contractions, and other irregular heart rhythms. Early detection of these arrhythmias can prevent severe complications such as stroke, heart failure, and sudden cardiac death. Advanced continuous rhythm monitoring is essential for personalized and preventive cardiac care, offering critical information that aids early intervention and supports effective chronic disease management.

In response, we have conducted two in-depth analysis to enhance the qualitative biosensing capabilities of the device, particularly for cardiovascular monitoring and autonomic nervous system monitoring. First, for real-time blood pressure-related monitoring, we analyzed pulse arrival time (PAT) before and after exercise. During desk work, the PAT is relatively high ranging from 280 ~ 320 ms. In contrast, following exercise, the PAT shows noticeable changes, with a shorter interval between the ECG and PPG peaks. This reduction in PAT reflects an increase in blood pressure, which is typical after physical exertion due to elevated heart rate and vascular responses. The significant difference in PAT between desk work and post-exercise conditions verifies the device's capability to detect real-time cardiovascular changes associated with varying physiological states. This capability suggests that fMMD can provide valuable

insights into cardiovascular health and potentially be used for continuous blood pressure monitoring in various everyday scenarios.

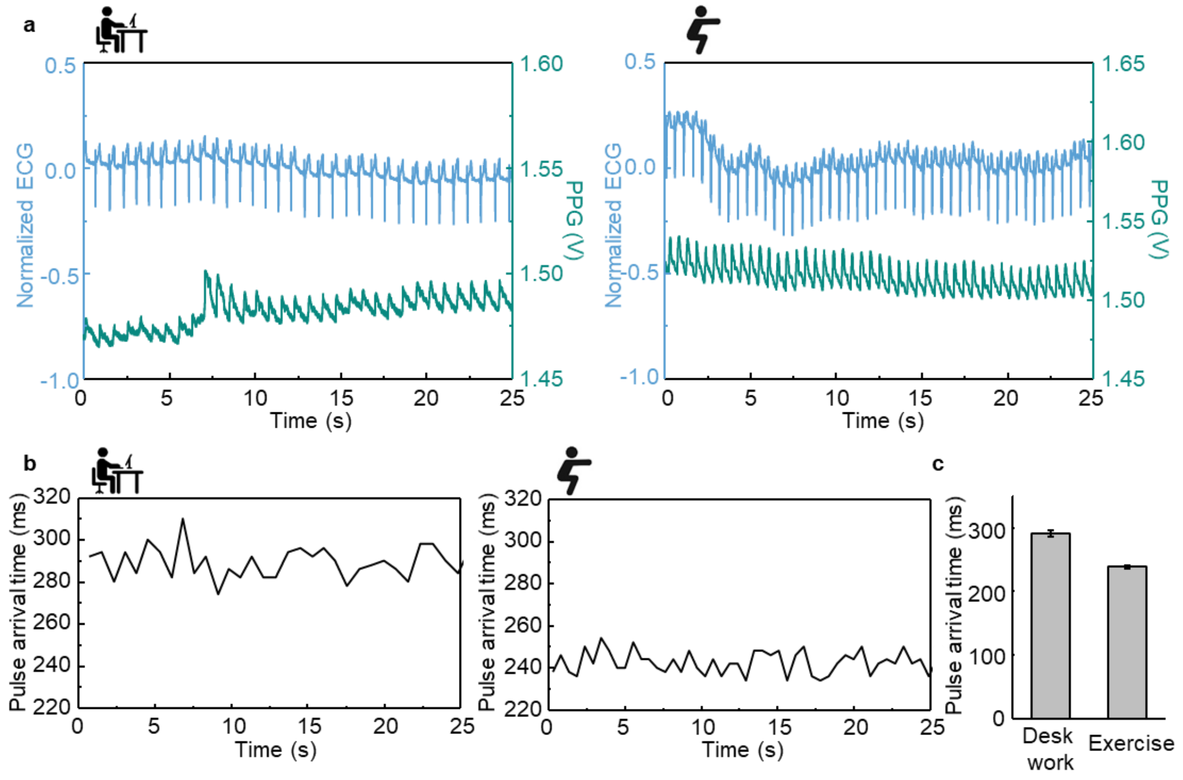

Second, we monitored heart rate variability (HRV) through ECG measurements taken immediately after exercise and continuing through the recovery period for several minutes. We demonstrated the body's recovery response after exercise, characterized by a gradual decrease in heart rate and a concurrent increase in HRV and the rate of HRV change. These trends provide valuable insights into the body’s autonomic nervous system balance and resilience, with HRV serving as a reliable indicator of relaxation and adaptive capacity. This approach allows for HRV monitoring as an indicator of autonomic nervous system function, which can provide valuable insights into stress levels and recovery states. Both measurements demonstrate the potential of this platform for continuous and qualitative cardiovascular and mental health monitoring.

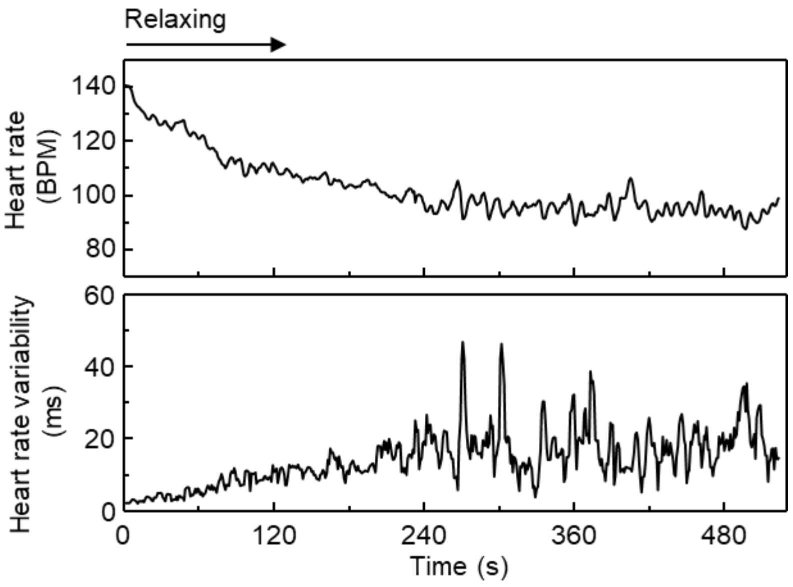

Also, skin-interfaced devices that combine electrochemical and physiological biomarkers enable comprehensive monitoring across various health domains by capturing biochemical and electrophysiological changes in real time[4]. Integrating lactate and ECG measurement, for example, allows for assessment of cardiovascular and metabolic health during physical exertion, supporting heart disease and respiratory disorder management. Hybrid systems that track glucose alongside heart rate, oxygen saturation, and activity levels offer targeted diabetes management, especially to prevent hypoglycemia post-exercise. For exercise physiology, tracking lactate, pH, and ECG enables real-time monitoring of metabolic acidosis and cardiac function, aiding in both performance assessment and recovery. Comprehensive wearable devices also monitor lifestyle and metabolic health, combining parameters like glucose, lactate, caffeine, alcohol, blood pressure, and heart rate during daily activities to provide actionable insights for managing diabetes, hypertension, and related conditions. Additionally, sensors for muscle contraction and strain can aid rehabilitation and prosthetic control by accurately tracking muscle activity and strain, demonstrating the utility of multiplexed biomarker analysis in both chronic disease and acute care contexts. As this technology advances, improvements in integration and precision will further enhance its applicability to personalized healthcare and disease management.

In terms of delivery platform, the development of a micropump suitable for acute care applications is an essential step in advancing wearable drug delivery technology. Currently, thermo-pneumatic actuation micropumps, which utilize controlled heating to generate pressure and facilitate drug release, are effective for continuous, low-flow-rate delivery suitable for chronic care. This type of steady, gradual administration is ideal for managing conditions that require long-term maintenance, such as diabetes (for insulin), hypertension (with medications like beta-blockers or calcium channel blockers)[5], and chronic pain (using analgesics)[6]. Such diseases benefit from stable, controlled drug release that maintains therapeutic levels over extended periods without necessitating immediate response. However, in acute care, rapid and

responsive drug delivery is critical to effectively manage sudden medical emergencies like cardiac arrest, asthma attacks, or severe pain, where immediate administration of medications such as epinephrine, bronchodilators, or opioids is essential[7]. Unlike chronic applications, acute care requires the micropump to deliver a higher volume of medication in a shorter timeframe to promptly stabilize the patient. Therefore, transitioning from chronic to acute applications demands several modifications to the micropump's design: enhancing the pumping rate and pressure for higher, faster dosing and ensuring it remains compact and portable to support wearable use. Developing an advanced pumping mechanism with faster response times and precise control can meet these high-delivery demands, enabling wearable devices to handle both chronic and acute care seamlessly, thereby broadening their clinical applications and utility in diverse healthcare settings.

In summary, we are pleased to report the successful further verification of cardiovascular and stress-related biomarker monitoring using this platform. By analyzing biomarkers such as pulse arrival time (PAT) for real-time blood pressure monitoring and heart rate variability (HRV) for stress assessment, we demonstrate the platform's capability for comprehensive biosignal monitoring. In response to the reviewer's comments, we have incorporated a discussion of possible solutions for optimizing the sensing-delivery-feedback design in the revised manuscript. This discussion further clarifies how real-time biosignal monitoring and qualitative analysis can be achieved, enhancing the platform's application in personalized healthcare.

Related references

1. Rajendra A. U. et al. Heart rate variability: a review. *Medical and biological engineering and computing*, **44**, 1031-1051 (2006)
2. Meidert, A. S. ., and Saugel B., Techniques for non-invasive monitoring of arterial blood pressure. *Frontiers in medicine*, **4**, 231 (2018)
3. Schultz K. et al. The days of the Holter monitor are numbered: Extended continuous rhythm monitoring detects more clinically significant arrhythmias in adults with congenital heart disease. *Circulation*, **134**, A13416-A13416 (2016).
4. Jung, H.H., et al. Wearable electrochemical sensors for real-time monitoring in diabetes mellitus and associated complications. **4**, 15, (2024)
5. Opie L. H. et al, Current Status of Safety and Efficacy of Calcium Channel Blockers in Cardiovascular Diseases: A Critical Analysis Based on 100 Studies. *Progress in cardiovascular diseases*, **43**, 171-96 (2000)
6. Lynch M. E. et al, The Pharmacotherapy of Chronic Pain: A Review. *Pain Resear ch and Management*, **11**, 11-38 (2006)
7. Zhu F. et al, Integrated Multifunctional Flexible Electronics Based on Tough Supra-molecular Hydrogels with Patterned Silver Nanowires. *Journal of Materials Chemistry C*, **8**, 7688-97 (2020)

Our modification to the manuscript:

We have added the supplementary Fig. 14, 15:

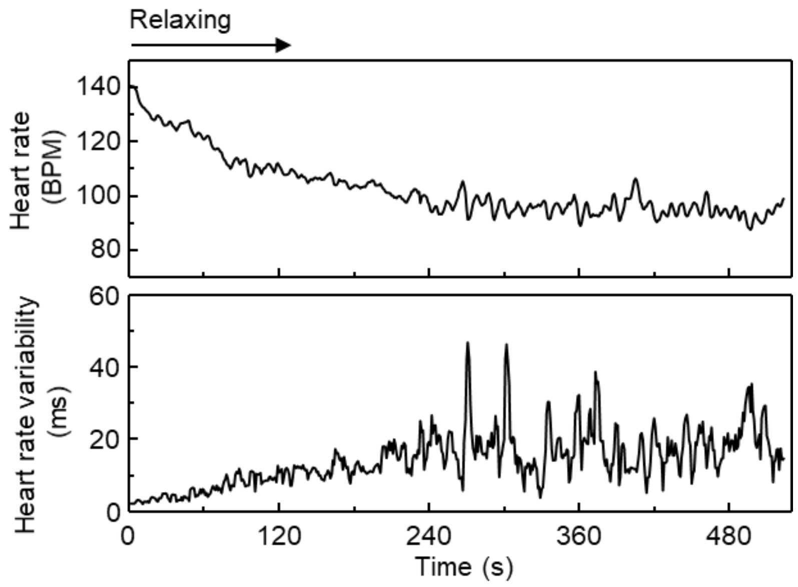

Supplementary Fig. 14 | Real-time measurement of heart rate and resulting heart rate variability during post-workout recovery.

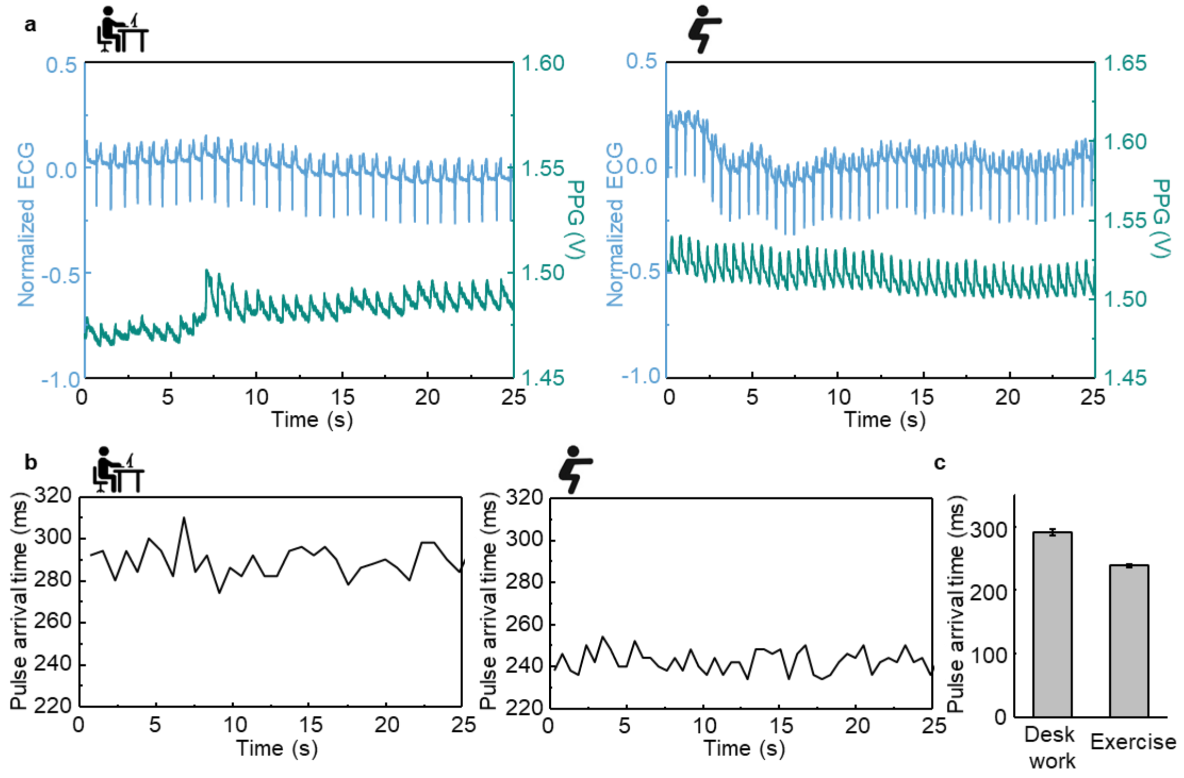

Supplementary Fig. 15 | Comparison of changes in pulse arrival time over time before

and after exercise. (a) Real-time measurement with optical and electrical modalities during desk work and after squat workout. **(b)** Calculation of PAT over time for two activities (desk work and exercise). **(c)** Comparison of average PAT for measuring time (3 trials).

In Page 17, we have updated the text about Supplementary Fig. 14 in Result section as follows:

Figure 5c shows the various electrophysiological signals, including ECG, skin temperature, and body movement, through an electrical modality of the chest mounted fMMD during the desk working and walking. The folded structure and strain-isolating elastomer allow the fMMD to reliable measurements by ensuring intimate contact with the skin and reducing mechanical stress, allowing stable acquisition of signals such as ECG and 3-axis accelerometer data and tracking three-dimensional reconfigured motion even during activities like sitting-to-standing transitions, and walking (Supplementary Fig. 13). Supplementary Fig. 14 demonstrates the capability of Case 1 measurement to perform heart rate and heart rate variability (HRV) analysis during post-workout recovery. The gradual decrease in heart rate and the corresponding increase in HRV and the rate of HRV changes reflect the body's shift to a relaxed state, indicating effective monitoring of autonomic function. This setup can be applied to cardiovascular health assessment and stress recovery monitoring in daily life.

In Page 18, we have updated the text about Supplementary Fig. 15 in Result section as follows:

The synchronized ECG and PPG from the fMMDs on the chest and wrist enable the monitoring of pulse arrival time (PAT) as shown in Fig. 5d (Case 2, single modal biosensing at multi-spots), which can be used as a medical indicator for sleep quality, arterial stiffness, and myocardial ischemia[46-48]. Calculation of the time interval between peaks of ECG and PPG enable to acquire the PAT as shown in Fig. 5e, f. Supplementary Fig. 15a, b show the capability of Case 2 measurement to monitor PAT changes before and after exercise, using synchronized ECG and PPG signals from the fMMDs on the chest and wrist. The average of PAT decreases following exercise (239 ms ± 2.9 ms) compared to desk work (291 ms ± 4.7 ms), which aligns with increased cardiovascular activity and elevated blood pressure after physical exertion. This real-time monitoring of PAT highlights the device's potential for blood pressure-related assessments and applications.

In the Discussion section, Page 20, we have revised the text as follows:

Additionally, the proposed fMMD has proven its capability to derive a multiplexed factor, such as real-time PAT, and HRV in diverse physiological states and trigger the self-directed drug delivery based on the collected signal as a closed-loop system.

In Method section, Page 27, we have updated the signal processing and calculation of heart rate variability.

Data analysis and algorithms for multiplexed measurement and closed loop operation

For real-time measurement, the fMMDs were attached on human subject's chest and wrist, respectively. Chest unit measured the ECG, skin temperature, and acceleration at a sampling rate of 500 Hz and wrist unit operated the PWM to trigger LD and measured PPG signal through a series of analog signal processing including analog filters, inverting amplifiers. The collected signals were digitized with 32-bit ADC and transmitted to the personal laptop. Signal processing used a detrend algorithm to remove baseline wander using analysis software (MATLAB R2023b). Peak detection algorithm acquired peak-to-peak time interval for ECG and PPG signals. PAT was calculated from the time intervals between simultaneously obtained ECG and PPG signal waveforms. In addition, the collected ECG signal from chest unit was used to calculate heart rate in real time, utilizing a window size of 5 s. For HRV calculation, a sliding window with a size of 5 s and an overlap of 1 s was applied to the ECG signal, using the intervals between peaks to compute HRV. The Root Mean Square of Successive Differences (RMSSD) was employed as the HRV metric, as it is particularly sensitive to high-frequency variations and provides an effective measure of parasympathetic nervous system activity, making it well-suited for assessing short-term changes in autonomic function. The data packet to initiate drug delivery was transmitted wirelessly from the MATLAB to the fMMD when the calculated heart rate exceeds 120 bpm as a way to show a closed-loop system.

Reviewer# 3

In this manuscript, the authors describe a multilayered, flexible, skin-mounted device made by folding an electronic circuit board. In addition to electrical circuits, they show that folding can be applied to chemical and optical modalities, and demonstrate a functional device by acquiring PPG, ECG, and temperature on a single subject. While the idea of folding multilayered circuit boards is simple and has been used in prior work, the extension to other modalities in a functional device is interesting and useful. I found the manuscript to be written and presented clearly. My specific comments are below:

Our response:

We sincerely appreciate the reviewer's helpful and detailed feedback on our manuscript. We are pleased that the extension of the folding process to integrate multiple modalities in a functional, skin-mounted device was found to be both interesting and useful. The reviewer's specific comments, particularly regarding the use of via-hole technology versus the folding process, have been extremely valuable in improving the logical flow and coherence of the manuscript. These insights helped us clarify our approach and present it more effectively. Additionally, the reviewer's suggestions to improve certain expressions and explanations have greatly contributed to making the manuscript more understandable for readers. We have carefully addressed each specific comment and revised the manuscript accordingly, ensuring that these improvements reflect the reviewer's valuable input. Thank you once again for your thorough review, which has played a significant role in enhancing the quality and clarity of our work.

Comment #1. Introduction: "However, this method requires complex procedures which are individually fabricating each single layer with via-holes, precisely aligning them, and connecting with conductive tape or paste."

a. Vias are a well developed technology in the PCB industry and can be reliably mass produced even on very thin PCBs. While I understand that standard industry processes cannot be used to produce stacked circuit layers that retain the flexibility of a single layer, it seems that they could be used to produce the double-sided circuit boards considered here. Could the authors clarify this statement, or focus more on the need for multimodal connectivity between layers rather than on the disadvantages of vias?

b. Following the above, the relative advantages and disadvantages of folding and vias should be discussed in a more nuanced manner in the Discussion section. I do not see folding as the superior choice to vias in many situations, including when many inter-layer connections are needed, the total thickness needs to be small, and there are limited placement options near the edges.

Our Response:

We appreciate the reviewer's constructive comments. In response to your suggestion, we have expanded the discussion by focusing on the necessity of folding. As mentioned in the first suggestion, double-sided and multi-layered structures can be fabricated using both via and folding methods [1]. Via structures are typically fabricated through sequential layer formation using manual drilling or etching. In contrast, folding structures are created through the folding process after the fabrication is complete. As mentioned in the Introduction, certain considerations must be taken into account when applying these structures to wearable devices with multimodal functionality. Specifically, rigid components must be integrated at the system level for multimodal functionality while maintaining flexibility to ensure comfortable adherence to the skin. Via structures, by enabling vertical connections, can be used to create thin double-sided structures, which can improve comfort when applied to wearable devices. However, if power management, communication, or signal processing modules are attached to one side of a thin, double-sided structure, it may lead to delamination from the skin. On the other hand, while folding-based structures may result in a thicker design, the inclusion of a strain-isolating layer can prevent delamination by placing rigid components on the side opposite to the skin. Therefore, this paper aims to develop a system-level multimodal device using a via-less folding structure.

Additionally, as suggested in the second suggestion, we have conducted a more thorough comparison of the advantages and disadvantages between folding and via-hole structures. Via-hole processes enable vertical stacking of chips, which reduces interconnection lengths and thus significantly improves both signal delay and power loss, contributing to enhanced system integration and miniaturization [2,3]. This process is particularly effective for devices using metal oxide semiconductors or chemically stable 2D semiconductors, as these materials are well-suited to lithography and etching-based fabrication techniques[4-6]. However, challenges arise when organic materials such as polyimide, Ecoflex, and PDMS are involved. These materials may suffer from alignment mismatches due to the differences in coefficients of

thermal expansion (CTE) between different substrates, exacerbating potential misalignments during processing. In particular, flexible materials may not maintain flat surfaces during drilling, leading to laser focus issues, while laser drilling itself can elevate substrate temperatures, risking damage to heat-sensitive materials. Etching-based via-hole processes can also introduce damage from solvent-based developers or plasma exposure, further complicating the integration of organic or flexible substrates. The complexity of via-hole fabrication varies significantly depending on the application. For instance, TSV-based interconnects for electrical, optical, and fluidic functions require different fabrication techniques for each modality, increasing the overall process complexity. Electrical vias are typically filled with conductive metals, optical vias utilize transparent materials such as SU-8, and fluidic vias remain hollow to allow the flow of fluids. [6-8] The need to employ distinct fabrication methods for each modality can add considerable complexity to the manufacturing process, impacting overall scalability and efficiency.

In contrast, the folding process offers a simplified approach that is highly advantageous for integrating multiple modalities into a single device. Since the structure is implemented by physically folding the device, there is no need for complex processes such as drilling, etching, or filling via-holes, resulting in a lower overall process complexity. This also eliminates the risk of alignment mismatches, which are common in more intricate fabrication methods. Consequently, the folding process can significantly reduce manufacturing time and costs, making it an attractive option for device fabrication. One of the main benefits of this simplified approach is the ability to easily integrate different functionalities (electrical, optical, fluidic), even when each modality is produced using different fabrication techniques. For instance, in the case of the discussed device, the drug delivery module was fabricated using a soft lithography-based molding technique, while the electrical connections were created using photolithography. Despite the differences in these manufacturing processes, the folding process allows for these components to be smoothly integrated into a cohesive system that operates efficiently. Furthermore, the folding structure enhances space efficiency by enabling the integration of multiple functions in a compact horizontal area. While several modalities could result in an increased device footprint, the folding technique reduces this impact by utilizing vertical space effectively. However, as the folding process builds upon the vertical dimension, there is a trade-off in terms of device thickness. Compared to traditional via-hole structures, the overall thickness of the device may increase slightly due to the stacked nature of the folded components. Nonetheless, the benefits of easier integration, reduced complexity, and cost-effective production make the folding process a compelling option for advanced multimodal devices.

In summary, both via-hole and folding processes have distinct advantages and disadvantages. Via-hole processes are ideal for creating vertical interconnections, significantly reducing signal delay and power loss, and facilitating miniaturization. However, they pose challenges with flexible materials and are complex, particularly when integrating multiple modalities. On the other hand, folding provides a simplified process, allowing for easier integration of various modalities and reduced complexity, but can result in slightly thicker devices. Given the specific requirements of the multimodal device proposed in this paper, the folding method is more

appropriate. Its flexibility, combined with manageable complexity, makes it the best option for achieving the desired functionality.

In accordance with the reviewer's suggestions, we have incorporated the points raised in a into the Introduction, and the feedback from b has been addressed in the Discussion section as outlined below. Through these revisions, we have aimed to deepen the analysis of the paper, providing a more comprehensive and well-rounded exploration of the subject matter.

Related references

1. Kim, S. et al. Vertically integrated electronics: new opportunities from emerging materials and devices. *Nano-Micro Lett.* **14**, 201 (2022).
2. Savidis, I. et al. Electrical modeling and characterization of through-silicon vias (TSVs) for 3-D integrated circuits. *Microelectron. J.* **41**, 9–16 (2010).
3. Jang, Y. J. et al. Advanced 3D Through-Si-Via and Solder Bumping Technology: A Review. *Materials* **16**, 7652 (2023).
4. Joo, H. J. et al. Oxide thin-film transistor-based vertically stacked complementary inverter for logic and photo-sensor operations. *Materials* **12**, 3815 (2019).
5. Zhao, C. et al. Evaporated tellurium thin films for p-type field-effect transistors and circuits. *Nat. Nanotechnol.* **15**, 53–58 (2020).
6. Ramesham, R. et al. Challenges in interconnection and packaging of microelectromechanical systems (MEMS). In *50th Electronic Components and Technology Conference*, 666–675 (IEEE, 2000).
7. Priyabadini, S. et al. 3-D stacking of ultrathin chip packages: An innovative packaging and interconnection technology. *IEEE Trans. Compon. Packag. Manuf. Technol.* **3**, 1114–1122 (2013).
8. Yoo, H. et al. Highly stacked 3D organic integrated circuits with via-hole-less multilevel metal interconnects. *Nat. Commun.* **10**, 2424 (2019).

Our modification to the manuscript:

In the Introduction section, Page 4-5, we have revised the text as follows:

As one of the integration methods, stacking through via-hole processes have been predominantly used[29-32]. Reliable methods for via-hole fabrication and filling have been well-established for conventional electronics; however, these approaches present limitations when applied to flexible and stretchable printed electronics[33]. Via-hole structures vary in design based on their application (e.g., electrical connections, optical pathways, or fluidic channels),[34-36] requiring precise alignment and integration with different materials, such as conductive pastes or SU-8, which increases process complexity. Furthermore, flexible materials commonly used in wearable devices, such as polyimide, Ecoflex, and PDMS, exhibit different coefficients

of thermal expansion (CTE), increasing the risk of alignment mismatches[37]. During the drilling process, these materials are also prone to damage, raising concerns about fluid leakage and device integrity.

As an alternative to via-hole process, a method has been developed that fabricates overall systems in a single layer and then folds them at once[38-40]. Despite these advantages of folding approach in terms of integrity and functionality, the folded structure of wearable device has so far been applied only to implementing electrical connections in a folded structure. This approach simplifies the fabrication process by removing via holes, facilitating the integration of multiple modalities. Additionally, it allows for conformal contact with the skin, even with the inclusion of rigid components, thereby enabling effective multimodal biosensing and drug delivery.

In the Discussion section, Page 19, we have revised the text as follows:

However, challenges exist from various aspects, including a system-level configuration, drug dose management, and integration into a single device without compromising flexibility or stretchability[9]. In terms of device fabrication, both via and folding methods can be considered for implementing double-sided and multilayer structures. The via method facilitates vertical stacking, which can be advantageous for system integration and miniaturization; however, the structural variation required for different applications increases process complexity and can lead to alignment mismatches and potential damage when integrated with flexible components.[37,60] In contrast, the folding method may result in a slightly thicker device but is advantageous for integrating multiple modalities due to its lower process complexity. Additionally, by incorporating a strain-isolating layer, the folding approach can prevent delamination from the skin caused by rigid components, making it more suitable for system-level multimodal devices. Accordingly, in this study, we introduce a unified platform by incorporating multiple modalities – electrical, optical, and chemical – with a folded structure, which enables multiplexed biosensing and on-demand drug delivery. We have designed functionally sectioned islands on a FPCB, connected by serpentine interconnects.

In Page 29-30, we have added the references:

33. Jansson, E. et al. Development of a full roll-to-roll manufacturing process of through-substrate vias with stretchable substrates enabling double-sided wearable electronics. Int. J. Adv. Manuf. Technol. 111, 3017–3027 (2020).
34. Khan, N. et al. 3-D packaging with through-silicon via (TSV) for electrical and fluidic interconnections. *IEEE Trans. Compon. Packag. Manuf. Technol.* **3**, 221–228 (2013).
35. Parekh, M. S. et al. High-performance interconnects for 3D ICs using through-silicon vias. In *2011 IEEE 61st Electronic Components and Technology Conference* (ECTC) 1992–1998 (IEEE, 2011).
36. Zheng, L. et al. Novel electrical and fluidic microbumps for silicon interposer and 3-D ICs. *IEEE Trans. Compon. Packag. Manuf. Technol.* **4**, 777–785 (2014).

37. Ramesham, R. et al. Challenges in interconnection and packaging of microelectromechanical systems (MEMS). In *2000 Proceedings. 50th Electronic Components and Technology Conference (Cat. No. 00CH37070)* 666–675 (IEEE, 2000).
60. Savidis, I. et al. Electrical modeling and characterization of through-silicon vias (TSVs) for 3-D integrated circuits. *Microelectron. J.* **41**, 9–16 (2010).

<u>Comment #2.</u> Can the authors provide actual images of the folded regions in Fig. 1 and 2 in addition to the illustrations?

Our Response:

Thank you for your helpful suggestion. In response to the reviewer's comment, we have updated Figure 2 to include actual images of the folded regions for the optical, chemical, and electrical modules**.** In Figure 1, we have further emphasized the explanation of the entire folded system through detailed illustrations. As the actual images of both the chip-integrated side and the skin-interfaced side (before and after folding) are presented in the Supplementary Fig. 1, we have kept Figure 1 focused on illustrations and a view of the fully integrated device.

Our modification to the manuscript:

We have changed the Fig. 2b to actual images of the folded regions showing electrical, optical, and chemical modalities, and modified the associated caption:

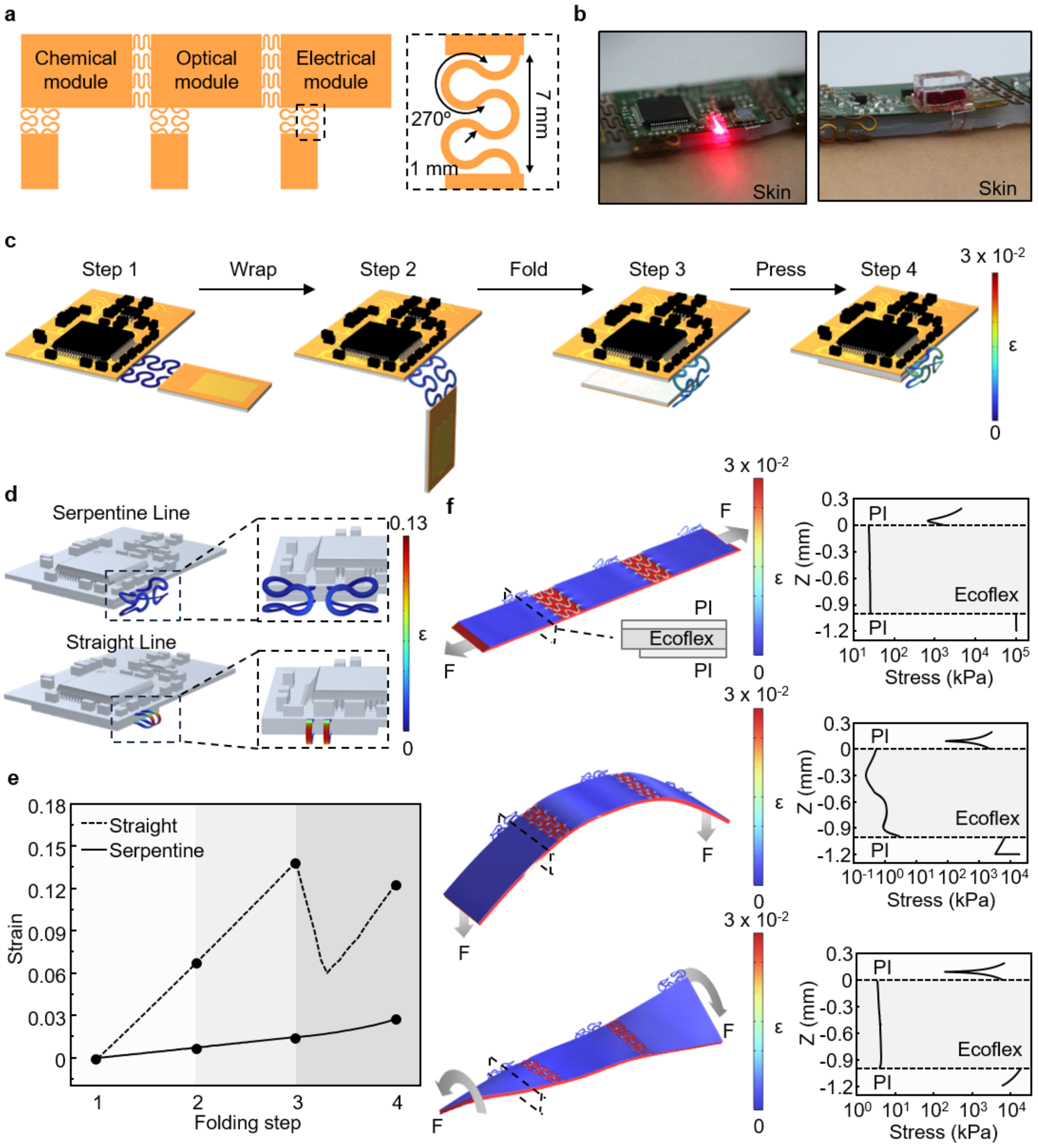

Figure 2. Design and Mechanical Properties of the fMMD. (a) Schematic design of the island-bridge substrate. Chemical, optical, and electrical modules are attached on the substrate, which ensures the structural stability through serpentine interconnects (black dashed box). **(b)** Photos of the fMMD attached on the skin representing different modalities. **(c)** Folding process of the substrate with a module and FEA simulation results of strain on the serpentine interconnects for each step. **(d)** Mechanical analysis of the principal strain in folded state for interconnects designed with serpentine lines versus straight lines. **(e)** Comparison of maximum strain on serpentine and straight interconnects during the folding process. **(f)** Strain and stress of fMMD under multi-deformation modes (stretching, bending, twisting). Strain-isolating effect observed for the deformations applied to the skin-interfaced layer through stress analysis at a cross-sectional plane (black dashed box).

Also, we have updated the text in Page 8:

The overall layout implementing the island-bridge structure with serpentine interconnects is illustrated in Fig. 2a. The chip-integrated substrate is designed to attach sub-systems, including biosensing modules, drug delivery module, and BLE communication modules. The optical image of the entire fMMD with these sub-systems in folded state is shown in Fig. 2b. As previously mentioned, the layout with the sub-systems attached integrates strain-isolating layers in both the chip-integrated layer (non-skin-contact side) and the skin-interfaced layer (skin-contact side).

Comment #3. It is unclear from Fig. 1 how the device is powered as the block diagram simply indicates "power" and the LiPo battery is not shown in the illustration.

Our Response:

We would like to thank the reviewer for this comment regarding to the power supply and management. The fMMD operates using a rechargeable LiPo battery. The LiPo battery and the power management IC units for charging, regulation, and switching are integrated and mounted on a single island within the device. In response to the reviewer's comment, we have revised Figure 1a and 1c accordingly. In Figure 1a, we add the LiPo battery in the illustration, and in Figure 1c, the term "power" has been updated to "power module" to more accurately represent the integration of the various components responsible for power supply and management. Additionally, we have provided a detailed explanation of the power module in the manuscript to clarify its role in the device's operation.

Our modification to the manuscript:

In Figure 1a, we add the LiPo battery in the illustration as follows:

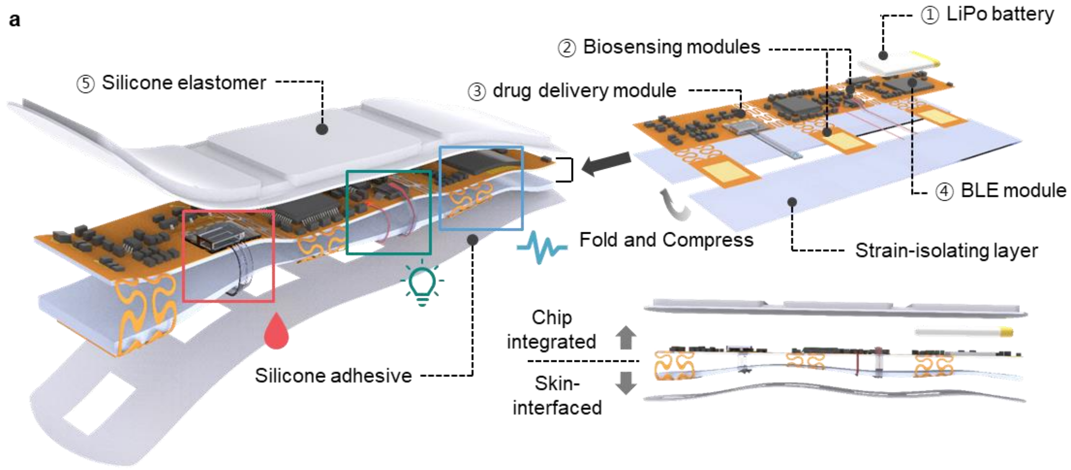

In Figure 1c, we modify the expression of "power" to "power module"

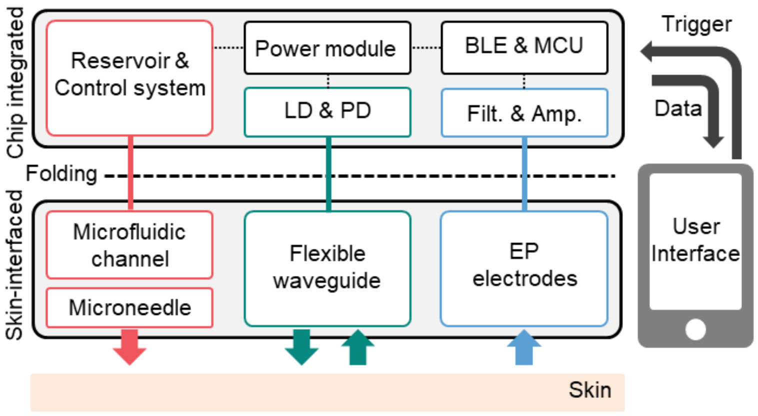

In Page 7, we add the detailed explanations for the power module.

The rigid, thick components are placed on chip-integrated side and the soft, thin components are placed on skin-interfaced side as shown in Fig. 1c. Each modality is integrated with a single platform (Supplementary Fig. 1a), and the folded configuration serves to define sections separating the elements that are in contact with the skin from those that are not (Supplementary Fig. 1b, c). Each functionality can be powered by a power module, which consists of a rechargeable LiPo battery, power switching circuitry, a voltage regulator, and a battery charger IC. The battery can be recharged through a USB port, ensuring continuous operation of the device.

<u>Comment #4.</u> Could the authors add references at the end of this sentence? "As an alternative to via-hole process, a method has been developed that 89 fabricates overall systems in a single layer and then folds them at once." (Line 88)

Our Response:

Thank you for your valuable feedback and the recommendation to include recent references in the folded system. We sincerely appreciate your insight into enhancing the comprehensiveness and relevance of our manuscript. In response to your suggestion, we have added the following references to our manuscript.

Related references

1. Yang, S. M., et al. Soft, wireless electronic dressing system for wound analysis and biophysical therapy. *Nano Today*. **47**, 101685. (2022)
2. Jeong, H., et al. Differential cardiopulmonary monitoring system for artifact-canceled physiological tracking of athletes, workers, and COVID-19 patients. *Science advances*. **7**, eabg3092. (2021)
3. Chung, H. U., et al. Skin-interfaced biosensors for advanced wireless physiological monitoring in neonatal and pediatric intensive-care units. *Nature medicine*. **26**, 418-429. (2020)

Our modification to the manuscript:

In Page 5, we have added the references:

As an alternative to via-hole process, a method has been developed that fabricates overall systems in a single layer and then folds them at once[38-40].

In Page 29, we have added the references:

38. Yang, S. M., et al. Soft, wireless electronic dressing system for wound analysis and biophysical therapy. *Nano Today*. **47**, 101685. (2022)
39. Jeong, H., et al. Differential cardiopulmonary monitoring system for artifact-canceled physiological tracking of athletes, workers, and COVID-19 patients. *Science advances*. **7**, eabg3092. (2021)

Comment #5. For the PPG sensor design in Fig. 4, there is potential concern that movement changes in the bending radius and introduces artifacts in the amplitude profile of the signal.

Our Response:

We sincerely appreciate the reviewer's constructive feedback. Following the reviewer's suggestion, FEA was conducted to examine transmittance when the device undergoes deformation due to movement while attached to the skin. Potential shape changes resulting from movement include variations in bending radius and shearing displacement. Given the device design, the bending radius is expected to remain relatively stable in the free-standing form once adhered to the skin. Therefore, variations of ±4 mm from the current bending radius were analyzed. Also, for potential misalignment due to shearing deformation between the laser-coupled and skin-attached portions of the waveguide, lateral displacements of 0.5 mm, 1.0 mm, and 1.5 mm were evaluated, which is the shearing deformation conditions of 100 %, 200 %, and 300 %, respectively. The results indicate that, even under these mechanical deformations, the normalized transmittance decreases by less than 15% compared to the undeformed state. These FEA results confirm that the amplitude remains sufficient for reliable heart rate and pulse arrival time (PAT) measurement using the PPG sensor.

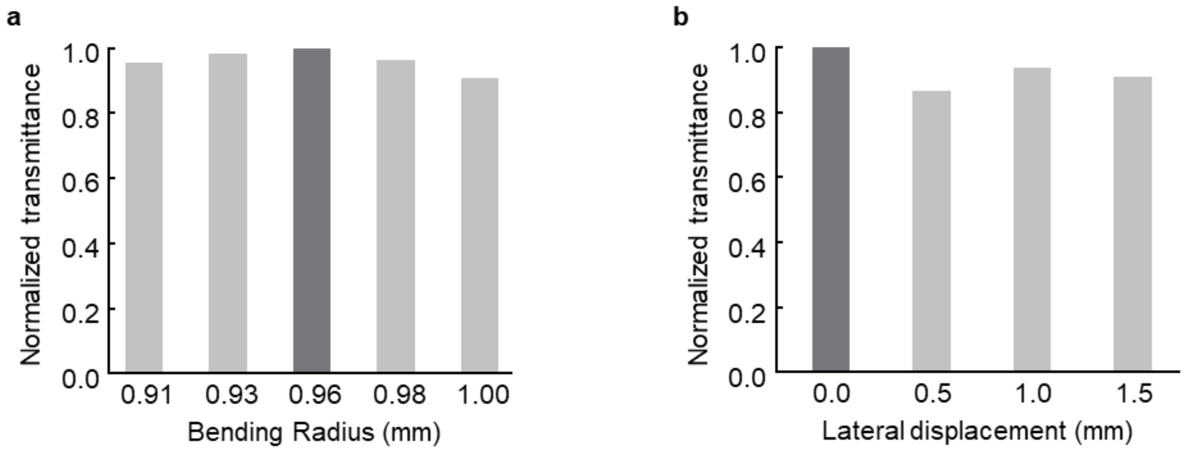

Additionally, we conducted further experimental analysis on PAT changes before and after exercise. Specifically, the real-time blood pressure monitoring function was analyzed by observing increases in heart rate and blood pressure due to vascular responses under both desk work and post-exercise conditions. These additional experiments confirmed the feasibility of PPG measurements. However, for SpO_2 measurements that do not rely on peak detection, amplitude changes may affect accuracy. Therefore, additional post-processing or modifications at the system level may be necessary to address this issue.

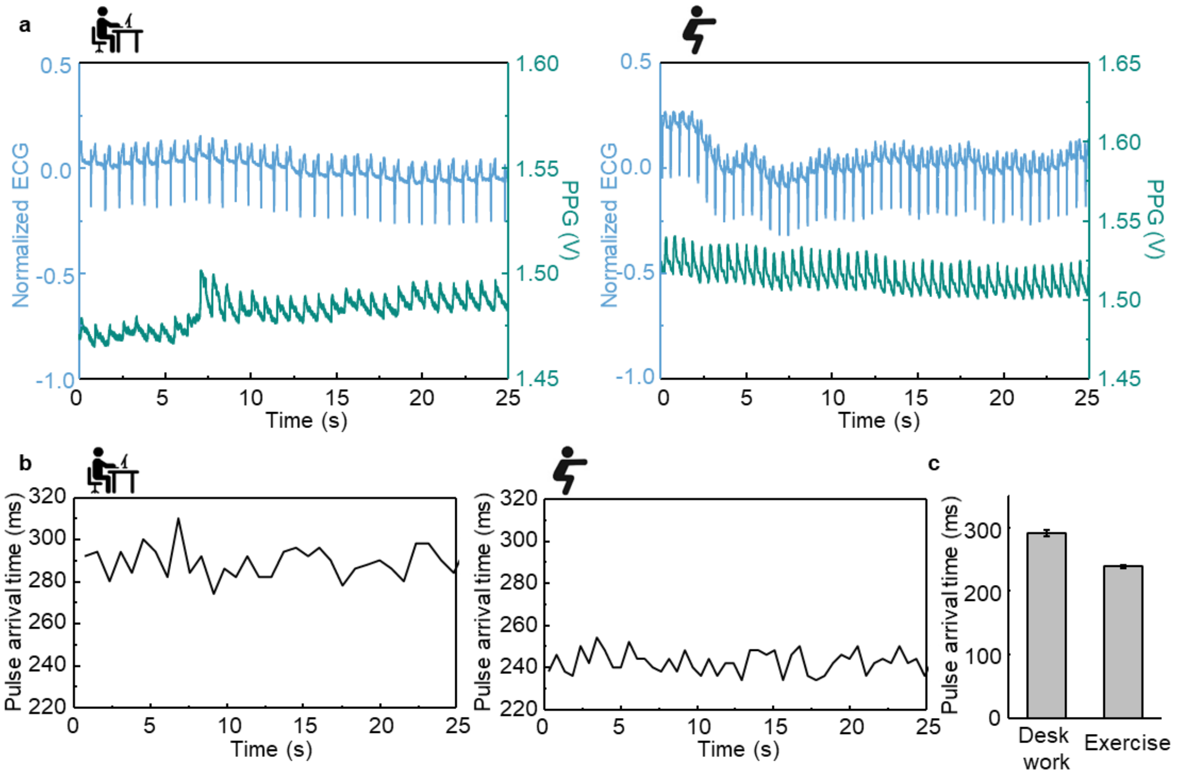

Based on these results, we anticipate that our designed PPG sensor will maintain reliable performance in measuring heart rate and PAT, despite potential deformations caused by movement. This suggests practical applications for continuous health monitoring in daily life settings.

Our modification to the manuscript:

We have added the supplementary Fig.11, 15:

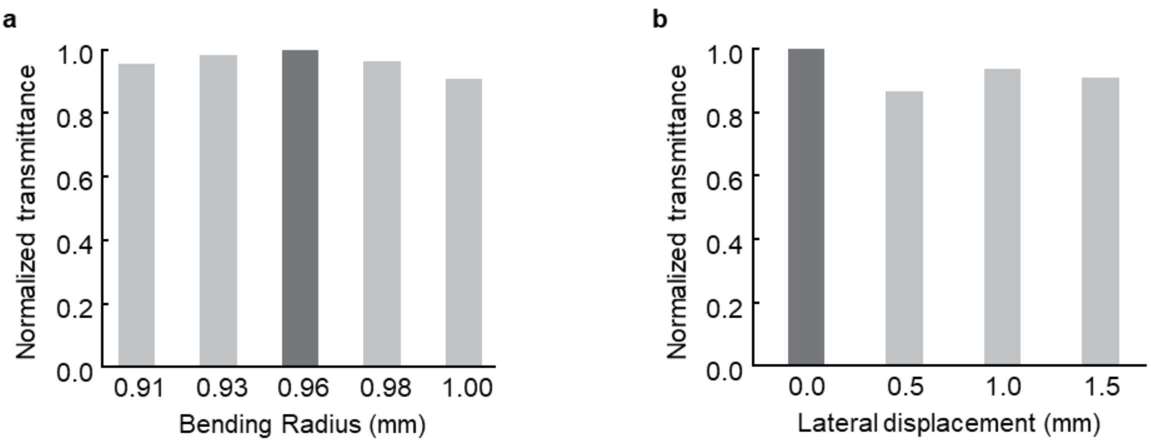

Supplementary Fig. 11 | Transmittance comparison of flexible PDMS waveguide under mechanical deformation using finite element analysis (FEA). (a) Normalized transmittance versus bending radius of flexible PDMS waveguide. A dark gray box represents the

undeformed state of the folded device. **(b)** Normalized transmittance versus lateral displacement of flexible PDMS waveguide due to shearing deformation. The waveguide attached to the skin is laterally displaced relative to the waveguide connected to the laser. A dark gray box represents the undeformed state of the folded device.

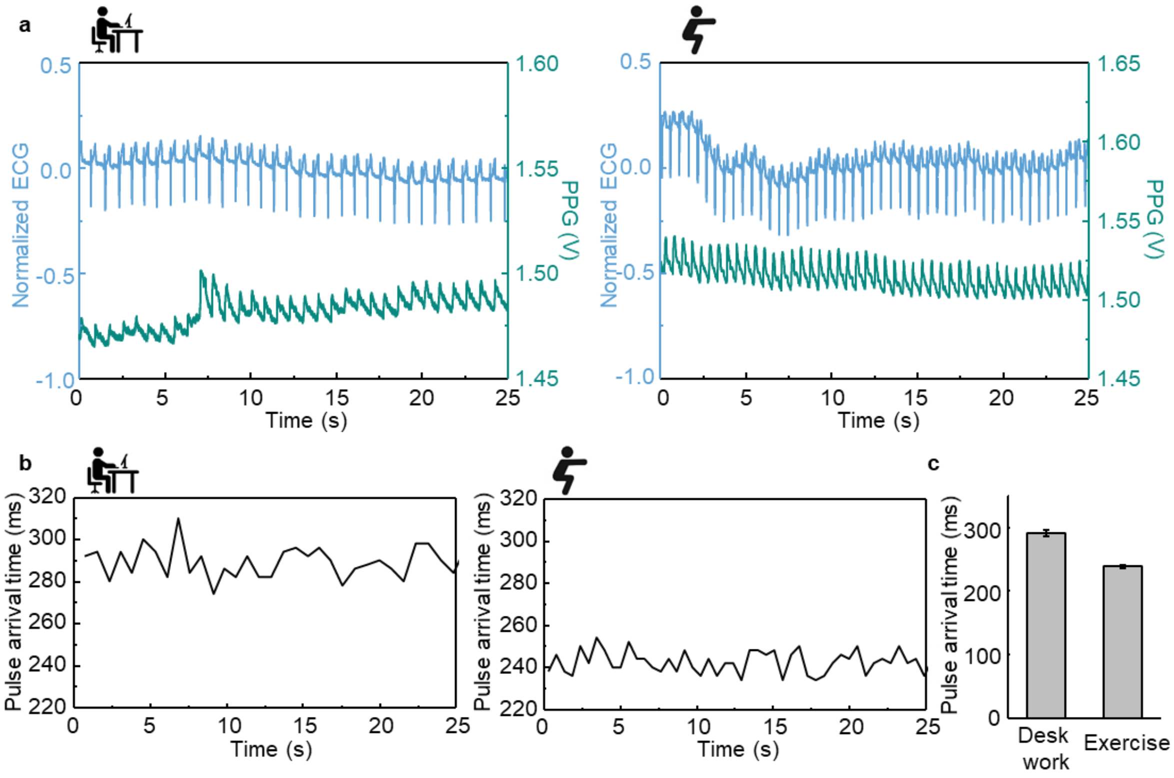

Supplementary Fig. 15 | Comparison of changes in pulse arrival time over time before and after exercise. (a) Real-time measurement with optical and electrical modalities during desk work and after squat workout. (b) Calculation of PAT over time for two activities (desk work and exercise). (c) Comparison of average PAT for measuring time (3 trials).

In Page 15, we have updated the text about Supplementary Fig. 11 in Result section as follows:

Although there is some loss of guiding light due to folding, the transmittance remains above 70 %, which is sufficient to monitor the PPG signal (Fig. 5e). Additionally, supplementary Fig. 11 examined the effects of potential shape deformations on transmittance when the device is attached to the skin during movement. The FEA analyzed transmittance variations resulting from changes in bending radius (±4 mm from the initial radius) and lateral displacements (0.5 mm, 1.0 mm, and 1.5 mm) between the skin-adhered lower waveguide channel and the laser-coupled upper channel. Under these mechanical deformations, the normalized transmittance decreases by less than 15% compared to the undeformed state when the device is attached to the skin.

<u>Comment #6.</u> Fig. 5. Was a pharmaceutically active drug actually delivered to the subject? If so, is there a measured effect? If not, it should be clear in the caption/Methods section that this is a sham drug.

Our Response:

Thank you for the insightful comment. We confirm that a pharmaceutically active drug was not delivered in this study; instead, a sham drug was used to validate the delivery mechanism showing the feedback operation. We have clearly specified this as a sham drug in the Figure 5 caption and the Methods section to avoid any confusion.

Additionally, regarding the potential applications of the drug delivery platform, we have expanded on this in the Discussion section. Based on the device's demonstrated ability to detect cardiovascular biomarkers, including real-time pulse arrival time (PAT) for blood pressure estimation and heart rate variability (HRV) for autonomic function assessment, we believe it could be adapted for applications involving blood pressure regulation through targeted drug delivery. For instance, the platform could be used to deliver antihypertensive agents such as sodium nitroprusside (SNP), which is commonly used for rapid blood pressure reduction in hypertensive crises. This capability would make the device a valuable tool for managing cardiovascular diseases by enabling on-demand drug administration based on real-time biosensing feedback.

Our modification to the manuscript:

In Page 27, we have updated the methods section as follows:

Data analysis and algorithms for multiplexed measurement and closed loop operation

For real-time measurement, the fMMDs were attached on human subject's chest and wrist, respectively. Chest unit measured the ECG, skin temperature, and acceleration at a sampling rate of 500 Hz and wrist unit operated the PWM to trigger LD and measured PPG signal through a series of analog signal processing including analog filters, inverting amplifiers. The collected signals were digitized with 32-bit ADC and transmitted to the personal laptop. Signal processing used a detrend algorithm to remove baseline wander using analysis software (MATLAB R2023b). Peak detection algorithm acquired peak-to-peak time interval for ECG and PPG signals. PAT was calculated from the time intervals between simultaneously obtained ECG and PPG signal waveforms. In addition, the collected ECG signal from chest unit was used to calculate heart rate in real time, utilizing a window size of 5 s. For HRV calculation, a sliding window with a size of 5 s and an overlap of 1 s was applied to the ECG signal, using the intervals between peaks to compute HRV. The Root Mean Square of Successive Differences (RMSSD) was employed as the HRV metric, as it is particularly sensitive to high-frequency variations and provides an effective measure of parasympathetic nervous system activity, making it well-suited for assessing short-term changes in autonomic function. The data packet to initiate drug delivery was transmitted wirelessly from the MATLAB to the fMMD when the calculated heart rate exceeds 120 bpm as a way to show a closed-loop system. The feedback-

actuated drug delivery module delivered a sham drug in a reservoir with a switching frequency of 1 Hz, and the micropump behaviour was filmed using an IR thermal camera.

In Page 20, the discussion section has been modified as follows:

In conclusion, our study represents a significant advancement in wearable healthcare technology, offering a closed-loop platform capable of intelligent monitoring and intervention. Leveraging multiplexed biosensing feedback, the drug delivery platform could be adapted for targeted blood pressure regulation through the administration of antihypertensive agents, such as sodium nitroprusside (SNP), enhancing its utility in managing cardiovascular conditions. Its potential applications extend beyond cardiovascular monitoring to include the management of blood glucose, pain, and various chronic diseases.

We have modified the Fig.5i caption as follows:

(i) IR thermal images of the fMMD before and after sham drug delivery trigger.
